# cRGD-Peptide Modified Covalent Organic Frameworks for Precision Chemotherapy in Triple-Negative Breast Cancer

**DOI:** 10.1021/acsami.4c10812

**Published:** 2024-09-13

**Authors:** Farah Benyettou, Mostafa Khair, Thirumurugan Prakasam, Sabu Varghese, Zineb Matouk, Maryam Alkaabi, Pilar Pena-Sánchez, Maylis Boitet, Rasha AbdulHalim, Sudhir Kumar Sharma, Rose Ghemrawi, Sneha Thomas, Jamie Whelan, Renu Pasricha, Ramesh Jagannathan, Felipe Gándara, Ali Trabolsi

**Affiliations:** † Chemistry Program, New York University Abu Dhabi (NYUAD), Abu Dhabi 129188, United Arab Emirates; ‡ Technology Innovative Institute, P.O. Box 9639, Abu Dhabi 9639, United Arab Emirates; § Instituto de Ciencia de Materiales de Madrid-CSIC, C. Sor Juana Inés de La Cruz 3, Madrid 28049, Spain; ∥ College of Pharmacy, Al Ain University, P.O. Box 112612, Abu Dhabi 112612, United Arab Emirates; ⊥ AAU Health and Biomedical Research Center, Al Ain University, P.O. Box 112612, Abu Dhabi 112612, United Arab Emirates; # Core Technology Platforms, New York University Abu Dhabi, 129188 Abu Dhabi, United Arab Emirates; ∇ Engineering Division, New York University Abu Dhabi, 129188 Abu Dhabi, United Arab Emirates

**Keywords:** covalent organic frameworks (COFs), targeted drug delivery, integrin-targeting RGD peptides, pH-responsive nanoparticles, triple-negative breast cancer (TNBC)

## Abstract

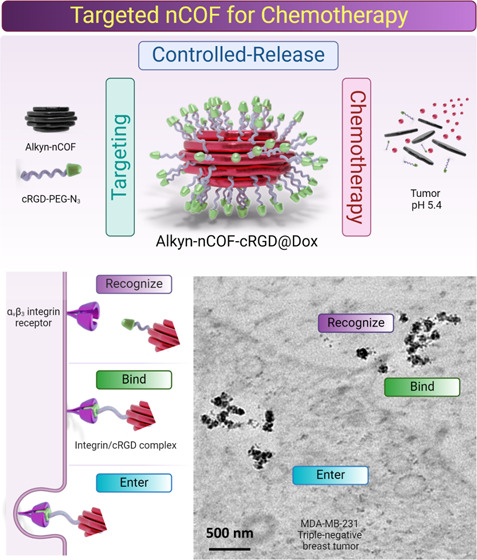

This study presents the use of nanoscale covalent organic frameworks (nCOFs) conjugated with tumor-targeting peptides for the targeted therapy of triple-negative breast cancer (TNBC). While peptides have previously been used for targeted delivery, their conjugation with COFs represents an innovative approach in this field. In particular, we have developed alkyne-functionalized nCOFs chemically modified with cyclic RGD peptides (Alkyn-nCOF-cRGD). This configuration is designed to specifically target α_v_β_3_ integrins that are overexpressed in TNBC cells. These nCOFs exhibit excellent biocompatibility and are engineered to selectively disintegrate under acidic conditions, allowing for precise and localized drug release in tumor environment. Doxorubicin, a chemotherapeutic agent, has been encapsulated in these nCOFs with high loading efficiency. The therapeutic potential of Alkyn-nCOF-cRGD has been demonstrated in vitro and in vivo models. It shows significantly improved drug uptake and targeted cell death in TNBC, highlighting the efficacy of receptor-mediated endocytosis and pH-controlled drug release. This strategy leverages the unique properties of nCOFs with targeted drug delivery to achieve significant advances in personalized cancer therapy and set a new standard for precision chemotherapeutic delivery.

## 1 Introduction

The search for improved drug delivery systems is a crucial step in the fight against cancer, especially aggressive forms such as triple-negative breast cancer (TNBC).^1−3^ Conventional drug delivery systems often have significant drawbacks such as nonspecific distribution, fluctuations in drug plasma levels, rapid clearance, and side effects on healthy tissue.^4−6^ These challenges underscore the urgent need for more targeted and efficient delivery methods.

Since their discovery in 2005 by Yaghi and co-workers,^7^ covalent organic frameworks (COFs) have significantly advanced the field of drug delivery systems, particularly in cancer therapy.^8−14^ These versatile materials are now being explored in various modalities of cancer therapy, from the traditional delivery of chemotherapeutic agents to alternative treatments like photodynamic therapy, photothermal therapy, and sonodynamic therapy (SDT), where their unique properties enhance the efficacy and specificity of treatment.^12,14−18^ Their substantial surface area and inherent porosity allow for high drug-loading capacities, which is critical for delivering effective therapeutic doses while minimizing the frequency of administration.^10,15,16,19^ The crystalline nature of COFs ensures uniform and predictable pore size throughout the framework, which is crucial for consistent drug loading and controlled release profiles, and increases the reliability of drug delivery systems.^9,10,19^ Additionally, this well-defined crystalline structure preserves structural integrity in the dynamic biological environments of the human body, which can enhance interactions with specific biomolecules or cellular targets.^12^ Moreover, the ability to customize the porosity and structural properties of COFs—thanks to a wide range of modifiable linkers—enables the development of innovative drug delivery systems that can release drugs in response to specific physiological stimuli such as pH changes.^10,11,20,21^ This pH-responsive behavior is particularly effective in targeting acidic tumor environments, optimizing treatment efficacy while minimizing the impact on healthy tissue.^12,14^ For example, we have recently reported the development of a nanoscale COF (nCOF) formed by the condensation of 2,6-diformylpyridine (DFP) with 1,3,5-tris(4-aminophenyl)-benzene (TAB) to form an imine-linked structure.^10^ This nCOF is stable under physiological conditions but decomposes in an acidic environment and enables site-specific drug release, demonstrating the potential of nCOFs in cancer therapy. These properties make COFs a versatile and powerful platform to advance cancer therapy and highlight their role in minimizing systemic toxicity while maximizing therapeutic outcomes.

Furthermore, most developments in the field of nanoscale COFs (nCOFs) have primarily exploited the effect of enhanced permeation and retention for tumor accumulation.^12,14^ This includes the modification of COF surfaces with molecules such as polyethylene glycol (PEG) to enhance the accumulation of nanoparticles (NPs) at tumor sites and facilitate the crossing of biological barriers.^22,23^ However, these methods are known to enhance the stability and circulation time of NPs without specifically targeting tumor cells. In contrast, recent advances in nCOF technology are shifting toward more active targeting techniques by precisely targeting specific membrane receptors to improve the delivery of NPs to tumor cells, such as the integration of specific targeting ligands like folic acid^24,25^ and hyaluronic acid^26^ on the surface of the nCOF structures for receptor-mediated targeting. Despite these advances, the integration of nCOFs with active targeting components is still rare. Challenges such as the complex chemical engineering required for precise functionalization, maintaining stability and compatibility in biological environments have slowed the development of targeted nCOFs and limited the volume of published research.^12,14^

**Figure 1:**
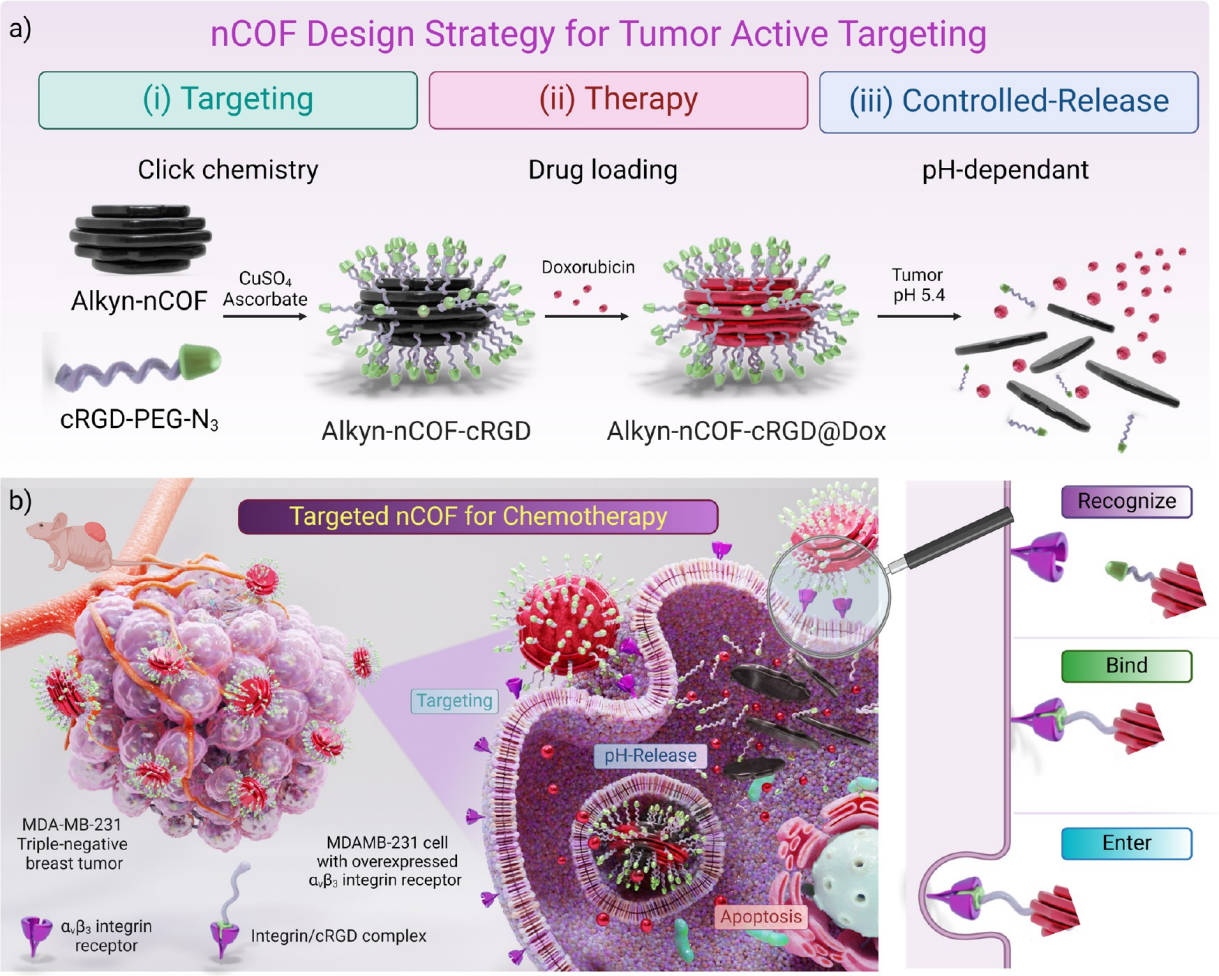
Targeted nCOF design for tumor active targeting and chemotherapy. (a) nCOF synthesis for tumor active targeting: (i) targeting: conjugation of cRGD-PEG-N_3_ peptides to Alkyn-nCOF via click chemistry, resulting in Alkyn-nCOF-cRGD. This specific modification targets α_v_β_3_ integrin receptors, which are overexpressed in TNBC cancer cells. (ii) Therapy: loading of the chemotherapeutic agent Doxorubicin (Dox) into Alkyn-nCOF-cRGD to form Alkyn-nCOF-cRGD@Dox. This process enhances the delivery of the therapeutic payload directly to tumor cells. (iii) Controlled-release: pH-dependent release of Dox, triggered by the acidic environment typically found in tumors, enabling targeted therapy with reduced systemic toxicity. (b) Mechanism of action within the tumor environment: targeting: specific binding of Alkyn-nCOF-cRGD to α_v_β_3_ integrin on the surface of tumor cells. pH-release: upon entering the tumor’s acidic environment, the nCOF triggers the release of Dox, enhancing the drug’s effectiveness right at the tumor site. Therapy: the released Dox acts directly on the cancer cells, promoting cell death and effectively reducing tumor size via apoptosis. This schematic illustrates the multifunctional approach of using engineered nCOFs for targeted chemotherapy, highlighting the synthesis, functionalization, and mechanism of action within a tumor environment.

Our strategy is based on nanoscale imine-linked COFs (nCOFs) conjugated with tumor-targeting peptides specifically designed for precision drug delivery in TNBC. We have prepared alkyne-functionalized nCOFs, (Alkyn-nCOF) modified with cyclic Arginyl-glycyl-aspartic acid (RGD) peptides (Figure 1a) to target the α_v_β_3_ integrins overexpressed in TNBC cells.^27,28^ This modification enhances receptor-mediated endocytosis, while the nCOF structure facilitates controlled release of the chemotherapeutic agent doxorubicin (Dox) in the acidic tumor microenvironment. Together, these features significantly improve its therapeutic efficacy (Figure 1b).

By providing a sophisticated, targeted approach to cancer therapy, this technology not only fills existing gaps in drug delivery research (Table S1 for comparison with existing RGD-conjugated NP systems) but also sets new standards in personalized medicine, highlighting the unique advantages of nCOFs in targeted therapeutic applications and advancing the field of nanomedicine toward more effective cancer treatments.

## 2 Experimental Section

### 2.1 Materials

All reagents and starting materials were sourced from Sigma-Aldrich and used without further purification. cRGD-PEG-N_3_ (MW 1k) was purchased from BOC Sciences and used without further purification. Deionized water was obtained from a Millipore Gradient Milli-Q water purification system. Solvents were purified as per standard protocols.

### 2.2 Synthesis of Alkyn-nCOF and Alkyn-nCOF-cRGD

The synthesis involved condensation of 4-ethynyl-2,6-diformyl pyridine (Alkyn-DFP, 12 mg, 0.06 mmol, 1 equiv.) and 1,3,5-tris(4-aminophenyl)benzene (TAB, 21 mg, 0.06 mmol, 1 equiv.) in anhydrous 1,4-dioxane using an acetic acid catalyst (0.5 mL, 3M) under microwave irradiation (110 °C, 2 h). Subsequent functionalization with cRGD-PEG-N_3_ was performed via copper-catalyzed azide–alkyne cycloaddition in aqueous conditions.

### 2.3 Drug Loading and Release

Dox was loaded into the COFs by impregnation at neutral pH, and its release was studied at various pH conditions via dialysis, tracking the release kinetics using fluorescence spectroscopy.

### 2.4 Characterization Techniques

Fourier transform infrared (FT-IR) spectra were recorded on a Agilent Technologies Cary 600 Series FTIR Spectrometer using the ATR technique. Powder X-ray diffraction (PXRD) patterns were obtained using a X-ray Panalytical Empyrean diffractometer with Cu Kα radiation. The samples were scanned from 5 to 40° (2θ) at a rate of 1° min^–1^. Transmission electron microscopy (TEM) images were obtained using a Thermo Fisher Scientific (TFS) Talos F200X scanning/transmission electron microscope (S/TEM) operating at 200 kV acceleration voltage. Samples for the TEM investigation were prepared by placing a 3 μL drop of the NP suspension on a carbon-coated copper grid (TED PELLA, Inc.) and allowing the solvent to evaporate. UV-visible absorption spectra were recorded with an Agilent Technologies Cary 5000 Series UV-Vis-NIR Spectrophotometer in water at room temperature (298 K). XPS experiments were carried out on a Kratos Axis Ultra DLD spectrometer under a base pressure of ∼2 × 10^−10^ mbar. Low-pressure gas adsorption measurements were performed on 3-Flex Surface Characterization Analyzer (Micromeritics) at relative pressures up to 1 atm. Thermogravimetric analysis (TGA) was performed on a TA Instruments SDT Q600 TGA from 25 to 800 °C at a heating rate of 10 °C min^–1^ under nitrogen. Dynamic light scattering (DLS) and Zeta Potential measurements were carried out on a Malvern Zetasizer NanoSeries at 25 °C. Flow cytometry analyses were performed on Attune NxT Flow cytometer.

### 2.5 Cell Culture

Human breast cancer cell lines adenocarcinoma MCF-7 (ATCC HTB-22) and MDA-MB-231 (ATCC HTB-26) cell lines were cultured in Dulbecco’s modified Eagle’s medium (DMEM) supplemented with 10% fetal bovine serum (FBS) and 1% penicillin/streptomycin at 5% CO_2_ and 37 °C.

### 2.6 In Vivo Biological Studies

All animal experiments were conducted in accordance with the policies of the New York University Institutional Animal Care and Use Committee (IACUC). Athymic NU/J nude mice (4–6 weeks old, approximately 20 g) were housed under standard conditions, including 12-h light/dark cycles, with ad libitum access to food and water. All animal procedures were approved by the IACUC of NYUAD.

### 2.7 Tumor Model Establishment

The MDA-MB-231 cancer model was employed as an example of aggressive TNBC to evaluate the therapeutic effect of the different Dox formulations. 5 × 10^6^ MDA-MB-231 cells in 200 μL of DMEM medium were injected subcutaneously into the right axillary region of nude mice. The mice were utilized in subsequent experiments after the tumor size had reached approximately 75–100 mm^3^.

### 2.8 In Vivo Antitumor Efficacy

To assess the antitumor effect, tumor-bearing mice were randomly divided into four groups (control *n* = 11, Dox *n* = 15, Alkyn-nCOF@Dox and Alkyn-nCOF-cRGD@Dox *n* = 14) and injected with 0.1 mL of saline, Dox alone ([Dox] = 5 mg kg^–1^), Alkyn-nCOF@Dox and Alkyn-nCOF-cRGD@Dox ([Dox] = 5 mg kg^–1^, [NP] = 10 mg kg^–1^, 200 μL) via intraperitoneal injection every 2 days for 20 days. Animal weight and tumor volume was measured before each injection. Tumor size was monitored via caliper measurement, and the tumor volume was estimated using: *V* = 0.5 × length × (width)^2^. On day 20, the mice were sacrificed, and the tumor mass and main organs were harvested, weighed, and photographed.

### 2.9 Statistical Analysis

All statistical analysis was performed with GraphPad PRISM 8. All data are expressed as mean ± SD. Data were analyzed using one-way ANOVA with post hoc Tukey tests SPSS (IBM, SPSS Statistics, version 23, USA). *p < 0.05; **p < 0.01; ***p < 0.001.

## 3 Results and Discussion

### 3.1 Synthesis and Characterization of Alkyn-nCOF and Alkyn-nCOF-cRGD NPs

**Figure 2:**
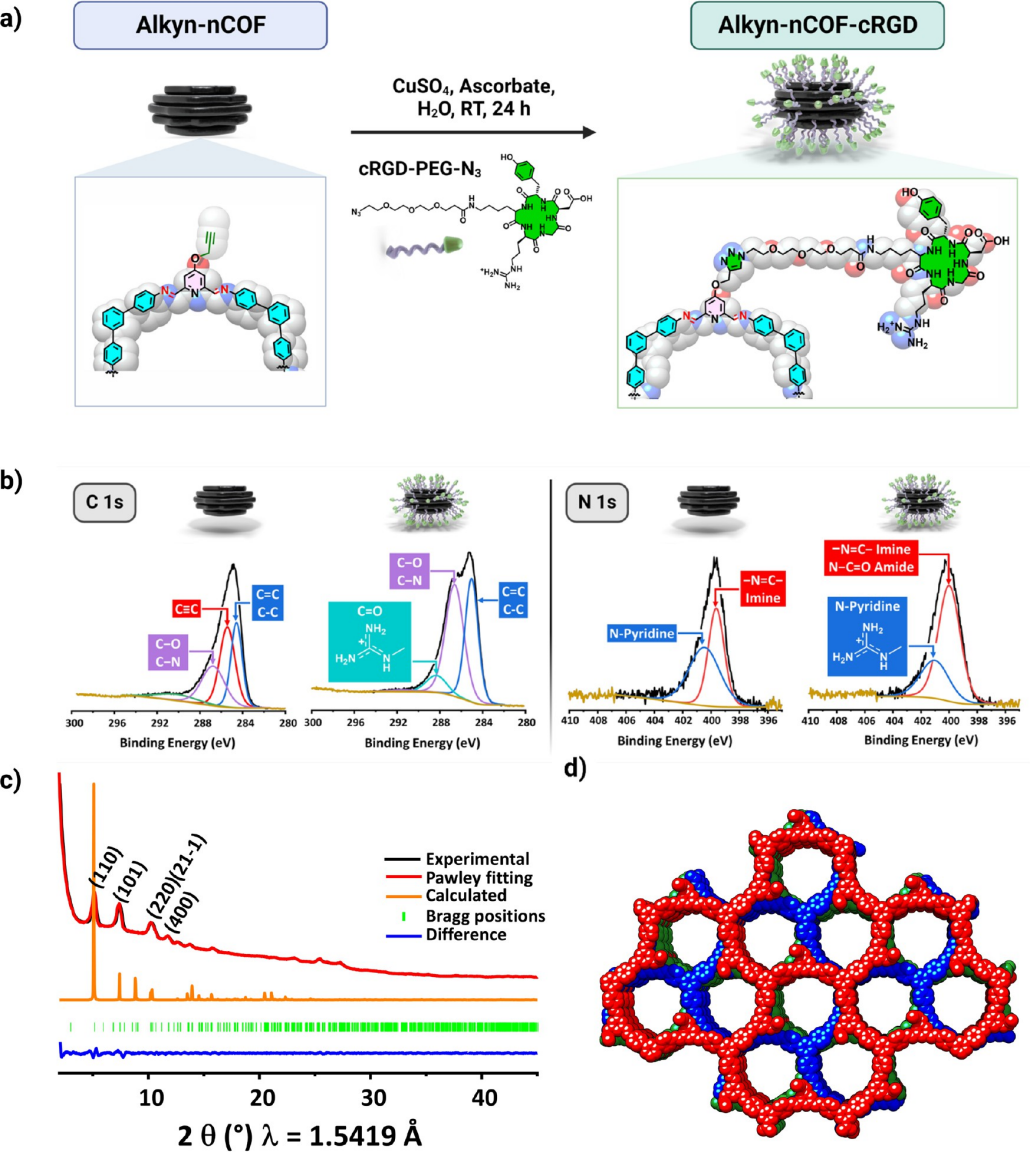
cRGD-PEG-N_3_ successful click cycloaddition on Alkyn-nCOF surface. (a) Chemical structure and synthetic route of Alkyn-nCOF-cRGD obtained by copper-mediated cycloaddition of cRGD-PEG-N_3_ on the Alkyn-nCOF NP surface. (b) XPS C 1s and N 1s spectra of Alkyn-nCOF and Alkyn-nCOF-cRGD. (c) Experimental and calculated PXRD patterns with Pawley fitting, Bragg positions, and differences of Alkyn-nCOF. (d) Space-filling representation of the stacked layers of the Alkyn-nCOF, where each color represents individual layers.

Porous imine-linked Alkyn-nCOF NPs with an average diameter of ∼60 nm were synthesized by co-condensation of 4-ethynyl-2,6-diformyl pyridine (Alkyn-DFP) and 1,3,5-tris(4-aminophenyl)benzene (TAB) in anhydrous 1,4-dioxane in the presence of acetic acid catalyst under microwave irradiation at 110 °C for 120 min (Figure S1). The as-synthesized brownish gel was collected by centrifugation and washed with 1,4-dioxane and ethanol, followed by water. The Alkyn-nCOFs are highly dispersible in water, facilitating their use as a drug delivery system. To incorporate the active targeting function into the Alkyn-nCOF nanocarriers, conjugation of the azide-pegylated cyclic RGD peptide (cRGD-PEG-N_3_, MW 1k) was performed in a one-step procedure by copper-mediated click chemistry in water (Figure 2a). Copper sulfate hexahydrate (3.2 mg, 0.02 mmol, 200 μL) and sodium ascorbate (12 mg, 0.06 mmol, 200 μL) were added to the Alkyn-nCOF solution (10 mg, 2 mL, pH 7).^29^ The resulting brown solution was stirred for 10 min at room temperature, then cRGD-PEG-N_3_ (10 mg, 100 μL) was added dropwise and stirred at room temperature overnight. The as-synthesized particles were collected by centrifugation, washed with water, and dialyzed for 48 h. The obtained NPs are referred to as Alkyn-nCOF-cRGD.

The chemical composition of Alkyn-nCOFs and Alkyn-nCOF-cRGD was investigated by FTIR spectroscopy, cross-polarization magic-angle spinning nuclear magnetic resonance (CP/MAS NMR) and X-ray photoelectron spectroscopy (XPS).

The FTIR spectrum of Alkyn-nCOFs shows a weak stretching band at 1622 cm^–1^ corresponding to the imine C=N bond, two broad and intense peaks at 1585 and 1345 cm^–1^ attributed to the C=N and C–N bonds of the pyridines and a strong vibrational peak at 1515 cm^–1^ corresponding to the aromatic C=C bonds (Figures S2 and S3). Additionally, vibrational peaks corresponding to the C–O–CH_2_–C≡C–H group of the Alkyn-DFP at 1443 cm^–1^ are evident, consistent with C–O stretching, and at 626 cm^–1^, corresponding to the C–H bending vibration of the terminal alkyne and the corresponding bond bending overtone at 1293 cm^–1^, originating from an out-of-plane overtone (harmonic) of the C≡C–H bending. This overtone absorption appears at twice the fundamental frequency. The disappearance of the N–H bonding of the amine in the range 3400–3200 cm^–1^ and the attenuation of the C=O signa at 1705 cm^–1^ (Figures S2 and S3) indicate a complete consumption of the starting materials. Following conjugation of the cyclic peptide cRGD-PEG with the Alkyn-nCOF NPs, changes in the C–O–CH_2_–C≡C–H group of the Alkyn-pyridines of the framework were observed (Figure S4). While the C–O stretching vibration at 1442 cm^–1^ remained unchanged, the terminal alkyne C–H bending vibration at 626 cm^–1^ and the corresponding bond bending overtone at 1293 cm^–1^ disappeared completely, while the other peaks became broader due to the presence of the peptide (Figure S4).

The CP/MAS NMR spectrum of Alkyn-nCOFs (Figure S5) shows chemical shifts from the aromatic carbons (100 to 150 ppm), the carbons of the C≡C alkyne (∼78 ppm), –OCH_2_ (∼57 ppm), and the imine (150 to 170 ppm). The absence of the aldehyde signal at ∼200 ppm confirms the complete conversion of the monomers. The spectrum of cRGD-PEG-N_3_ shows chemical shifts of the aliphatic (10 to 80 ppm), aromatic (120 to 140 ppm), guanidine (∼158 ppm), and carbonyl (165 to 180 ppm) carbons. The successful reaction between the cRGD-PEG peptide and the Alkyn-nCOFs was confirmed by the disappearance of the alkyne peaks at ∼78 ppm and by the appearance of new peaks at ∼70 ppm belonging to the –OCH_2_ groups of the cyclic peptide. This latter peak is broad compared to those of the free peptide, indicating the successful coupling of cRGD-PEG to the nCOF.

The chemical composition of Alkyn-nCOF and Alkyn-nCOF-cRGD was also confirmed by XPS. In the high-resolution C 1s XPS spectra of Alkyn-nCOF, the three peaks with binding energies at (i) 284.6, (iii) 285.4, and (iii) 286.8 eV were assigned to (i) C=C/C–C, (ii) C≡C,^30−32^ and (iii) carbon-oxygen/nitrogen bonds (C–O in Alkyn-DFP/C–N=C in the imine bond and the pyridine), respectively (Figure 2b and Table S2). The N 1s band was deconvoluted into two peaks at (i) 399.6 and (ii) 400.5 eV which are assigned to the nitrogen atoms in (i) the imine bond –N=C– and (ii) the N-pyridine, respectively (Figure 2b and Table S3).

High-resolution C 1s XPS spectra of cRGD-PEG-N_3_ revealed four peaks: (i) C=C/C–C at 284.7 eV, (ii) N–C=O at 286.5 eV, and (iii) the side chain COOH along with the guanidinium at 287.6 eV (Figure S7 and Table S2).^33^ In the high-resolution N 1s XPS spectra, the peptide has amide N–C=O linkages that lead to photoemission at 399.9 eV (Figure S7 and Tables S3), along with a small peak to higher binding energies at 401.4 eV arising from the protonated guanidine group.^33^ The presence of the azide N=N^+^=N^–^ functional group is evident at 404.6 eV, corresponding to the central electron deficient N atom in the azido group.^34^

After clicking the cRGD-PEG-N_3_ (Figure 2b, Tables S2 and S3), the N 1s initially at 399.64 in Alkyn-nCOF (fwhm = 1.39) broadened (fwhm = 1.83) and shifted to slightly lower energy (399.5 eV) in Alkyn-nCOF-cRGD spectra. Moreover, the intensity of the N 1s peak also increased (70%), indicating more N on the NP surface originating from the amide of the peptides. A weak contribution due to the presence of the –N= unit of the newly formed triazole rings and the charged guanidinium groups^35,36^ was identified as a shift and a broadening at ∼401.05 eV (fwhm = 2.6 compared to 2.4 for Alkyn-nCOF) in addition to the N-pyridine contribution of the nCOF. Additionally, no azide was detected, confirming no peptides are noncovalently bound to the NP surface.^34^

Furthermore, the C 1s region of Alkyn-nCOF-cRGD confirmed the successful peptide click and was deconvoluted into three peaks assigned to C=C/C–C (285.0 eV), C–O/C–N=C/N–C=O/triazole (286.6 eV), and the side chain COOH along with the guanidinium (288.3 eV) (Figure 2b and Table S2). The peak at 285.0 eV assigned to C=C/C–C is more intense and broadened (fwhm = 1.44 compared with 1.15 for Alkyn-nCOF), clearly indicating the presence of more C on the clicked surface due to new species/interaction components between the Alkyn-nCOF and the peptides on the surface. Significantly, no C≡C units (or significantly decreased and adsorbed in the 284.5 eV peak) were observed, indicating a successful click on the surface of the NPs. Moreover, the increase in cRGD-PEG surface concentration resulted in an increase in the intensity of C–O/C–N=C/N–C=O/triazole peak and the rise of a well-defined new peak corresponding to the COOH and guanidinium (288.3 eV).^37,38^ It is also important to note that no catalyst (CuSO_4_·5H_2_O) residues were detected by XPS or ICP–MS, which could lead to undesirable toxicity.

PXRD measurements were recorded to confirm the crystallinity of Alkyn-nCOF (Figure 2c). The PXRD pattern shows the most intense diffraction peaks at 2θ = 5.1° (110), 7.4° (101), 10.3° (220)/(21-1), indicating the long-range order in the framework. Following the principles of reticular chemistry, crystal structure models were constructed (Figure 2c,d) based on the geometry of the building blocks, resulting in distorted hcb layers. A crystal structure model created in the *P*3 space group was geometrically optimized by energy minimization using a universal forcefield. The completed Pawley refinement supports the obtained unit cell with lattice parameters of *a* = *b* = 34.632 Å, and *c* = 13.009 Å, with three layers per unit cell and an interlayer spacing of ∼4.3 Å. The PXRD pattern of Alkyn-nCOF-cRGD shows the presence of peaks at 4.0, 13.0, and 24.6°. However, the intensity of these peaks decreased significantly compared to the pristine nCOF due to the presence of the peptide on the surface (Figure S9).^39−42^

**Figure 3:**
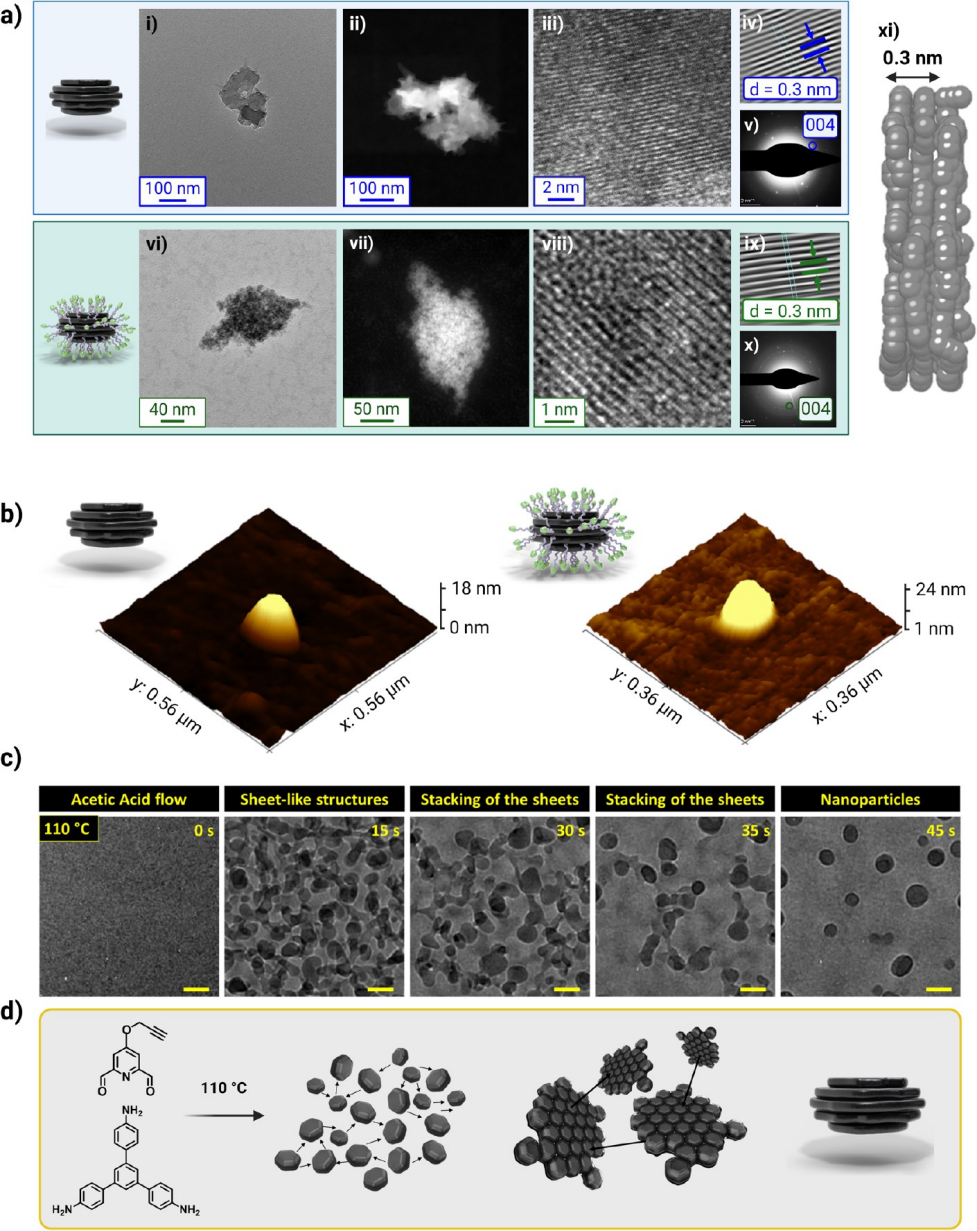
Morphological analysis. (a) HRTEM (i,vi) and STEM (ii,vii) images of Alkyn-nCOF (top) and Alkyn-nCOF-cRGD (bottom), respectively, demonstrating an average particle diameter of approximately 60 nm. Particles were negatively stained with a 1% uranyl acetate substitute to enhance contrast and highlight the presence of peptides on the NP surface. High-resolution images of lattice fringes (iii,viii) and their reconstruction with corresponding interlayer spacing (d) approximately 0.3–0.4 nm (iv,ix), and SAED patterns (v,x) for Alkyn-nCOF (top) and Alkyn-nCOF-cRGD (bottom). (xi) A space-filling model depicts three stacked layers of Alkyn-nCOF. (b) Representative atomic force microscopy (AFM) images Alkyn-nCOF (left) and Alkyn-nCOF-cRGD (right). (c) Variable-temperature liquid-cell transmission electron microscopy (VT-LCTEM) experiment: sequential LCTEM frames extracted from Movie 1 illustrating the in situ heating of TAB and Alkyn-DFP precursor solution to 110 °C in the presence of acetic acid. The initial frame shows the COF precursor solution at 110 °C without nanostructures at 0 s, while the following frames (15–45 s) capture the changes in a selected region as the solution is heated. An average particle size of approximately 60 nm is achieved within 60 s at 110 °C, maintaining stability over prolonged periods at this temperature. Scale bar = 50 nm. (d) Synthesis pathway from precursors to NPs: the diagram shows how molecular precursors (TAB and Alkyn-DFP) transform into Alkyn-nCOF at 110 °C. It traces the formation of sheet-like structures, their stacking, and rearrangement into spherical NPs.

Lattice-resolution TEM images of Alkyn-nCOFs and Alkyn-nCOF-cRGD suggested that they are crystalline with consistent and continuous lattice fringes that extend throughout the COF (Figure 3a). The lattice spacings were 0.3–0.4 nm, corresponding to the interlayer distances. Furthermore, the selected area electron diffraction (SAED, Figure 3a) demonstrates the well-crystallized feature of Alkyn-nCOFs and Alkyn-nCOF-cRGD, which exhibited distinct electron diffraction spots, that fit well with the *d*_004_ plane with *d*-spacing of 0.32 nm (Figure 3a).

HRTEM images showed Alkyn-nCOFs and Alkyn-nCOF-cRGD as spheroidal morphology with an average diameter of ∼60 nm (Table 1, Figures 3a and S11–S14). After clicking the peptide, the morphology, size, and height of Alkyn-nCOF-cRGD remained unchanged compared with pristine nCOF because the peptide size is insignificant (≈1.32 nm) (Table 1 and Figures S11–S14). To verify the presence of the peptide, the samples were negatively stained with uranyl acetate substitute (1%), which specifically stains the peptide, making it appear as dark spots in TEM;^43^ the differences between the Alkyn-nCOF and cRGD-conjugated nCOF are clearly visible. Small dark aggregates with an average size of 1–2 nm, corresponding to the peptides on the surface of the Alkyn-nCOF-cRGD, can be easily identified (Figure 3a(ii)). At the same time, the Alkyn-nCOFs do not exhibit these aggregates (Figure 3a(vii)).

**Table 1:** Physicochemical Characterization of the Alkyn-nCOF before and after Clicking cRGD-PEG-N_3_ on the NP Surface

	alkyn-nCOF	alkyn-nCOF-cRGD
PXRD, 2θ° (plane)	5.1° (110), 7.4° (101), 10.3° (220)/(21-1)	4.0, 13.0, 24.6°
TEM width (nm)	59.1 ± 8.4 nm	59.9 ± 9.5 nm
AFM height (nm)	22.65 ± 5.5	23.59 ± 3.4
BET (m^2^ g^–1^), (TPV*, m^3^ g^–1^)	140, (0.17)	95, (0.08)
pore width (Å)	10.2/13.1	10.1/13.1
ζ-potential (mV)	–6.7	–21.9
hydrodynamic diameter (nm)	220.0	92.2
Dox loading capacity (wt %)	61	42

AFM images in tapping mode provided additional evidence for the spheroidal morphology of the particles with a height of ∼23 nm (Figures 3b, S17, S18, Table 1). The mechanism of formation of Alkyn-nCOFs was directly monitored by in situ measurements using liquid cell TEM (LC-TEM, Movie 1, Figures 3c and S15). The two linkers, Alkyn-DFP and TAB, dissolved in dioxane, were drop-casted onto a bottom chip. Once wetting of the liquid cell was confirmed, acetic acid was flowed into the cell and the liquid cell was heated to 110 °C (Movie 1, Figures 3c and S15: extracted frames from Movie 1). The formation of sheet-like structures was observed within a few seconds. Then the nanosheets started to stack on top of each other, forming spherical particles highlighting the dynamic assembly process and the factors influencing their morphology and size (Figure 3d). The observations from LC-TEM explain why the diameter (60 nm) was larger than the height (23 nm).

The porosity of Alkyn-nCOFs was evaluated by measuring the adsorption–desorption isotherms of nitrogen gas at 77 K (Figure S19). Alkyn-nCOFs particles exhibit type-II isotherms with Brunauer–Emmett–Teller (BET) surface areas of 140 m^2^ g^–1^, two narrow pore size distributions with average pore widths of 10.2 and 13.1 Å, and a calculated pore volume of 0.17 cm^3^ g^–1^ (Figure S19 and Table 1). Due to the large size of the peptide, conjugation occurred only at the surface and not inside the pores, as confirmed by the analysis of BET. Indeed, a slight decrease in BET surface area (95 m^2^ g^–1^) and pore volume (0.08 cm^3^ g^–1^), but not in pore width (10.1/13.1 Å) were observed, confirming that the pores remained unaffected after the peptide was clicked (Figure S19).

The electronic properties of Alkyn-nCOF and Alkyn-nCOF-cRGD were studied by absorption and fluorescence spectroscopy in water. The UV–vis absorption for an aqueous dispersion of Alkyn-nCOF and Alkyn-nCOF-cRGD exhibits two maxima at λ_max_ = 283 and 366 nm (Figures S20 and S21). In addition, Alkyn-nCOF showed a broad emission band centered at a maximum of 468 nm when excited at 380 nm (Figure S22). The presence of the peptide on the surface of Alkyn-nCOF affected the emission of the material. After clicking the peptide, in addition to the broad emission peak of nCOF at 468 nm, an additional emission shoulder band centered at 438 nm corresponding to the emission of the peptide was observed (Figure S23).

TGA showed that Alkyn-nCOFs and Alkyn-nCOF-cRGD were stable up to 400 °C (Figure S26). Their chemical stability was also evaluated. The particles immersed in a PBS solution with a pH of 7.4 and kept at 37 °C for 24 h maintained their shape, size, and crystallinity (Figure S8). Under acidic conditions (pH = 5.4), the particles decomposed and lost their shape, size, and crystallinity, due to acid-catalyzed hydrolysis of the imine bonds (Figure S8).

The successful conjugation of the peptide to the nCOF surface was also confirmed by analyzing the surface charge at pH 7.4 (Figure S27). After clicking the peptide, the ζ-potential of the Alkyn-nCOF decreased significantly from −6.7 ± 0.5 to −21.9 ± 1.1 mV. This decrease in ζ-potential was attributed to surface modification with cRGD-PEG cyclic peptides, providing additional evidence for the effective attachment of the peptide to the nCOF surface.^44,45^ A likely explanation lies in the preferential orientation of the anionic carboxyl groups (in the aspartic acid residue) and the cationic guanidine group (in the arginine).^46^ As a consequence of the stronger negative charge and thus better interparticle repulsion, the average hydrodynamic diameter at pH 7.4 of Alkyn-nCOF-cRGD was 92.2 nm with a polydispersity (PDI) of 0.1 and no precipitation was observed over time (Figures S8 and S28–S31), in contrast to 220.0 nm with a PDI of 0.5 for Alkyn-nCOFs. At acidic pH, both systems decompose with a drastic decrease in particle size (Figures S8 and S28–S31).

The weight % efficiency of peptide conjugation on NP surfaces reflects the proportion of conjugated peptides to the total weight of the NPs. The amount of peptide on the nCOF surface was quantified using the *o*-phtalaldehyde (OPA) method,^29^ revealing a peptide conjugation efficiency of 54.2 wt % (Figure S35).

The properties of Alkyn-nCOF and Alkyn-nCOF-cRGD NPs are summarized in Table 1. These bulk characterizations indicate the successful synthesis of nanoscale imine-linked Alkyn-nCOF NPs. The presence of the alkyne functions was successfully conjugated to an azide-pegylated cyclic RGD peptide (cRGD-PEG-N_3_) by copper-mediated click chemistry. This prompted us to use it for the targeted delivery of Dox.

### 3.2 Loading and Release of Dox in Alkyn-nCOF and Alkyn-nCOF-cRGD NPs

**Figure 4:**
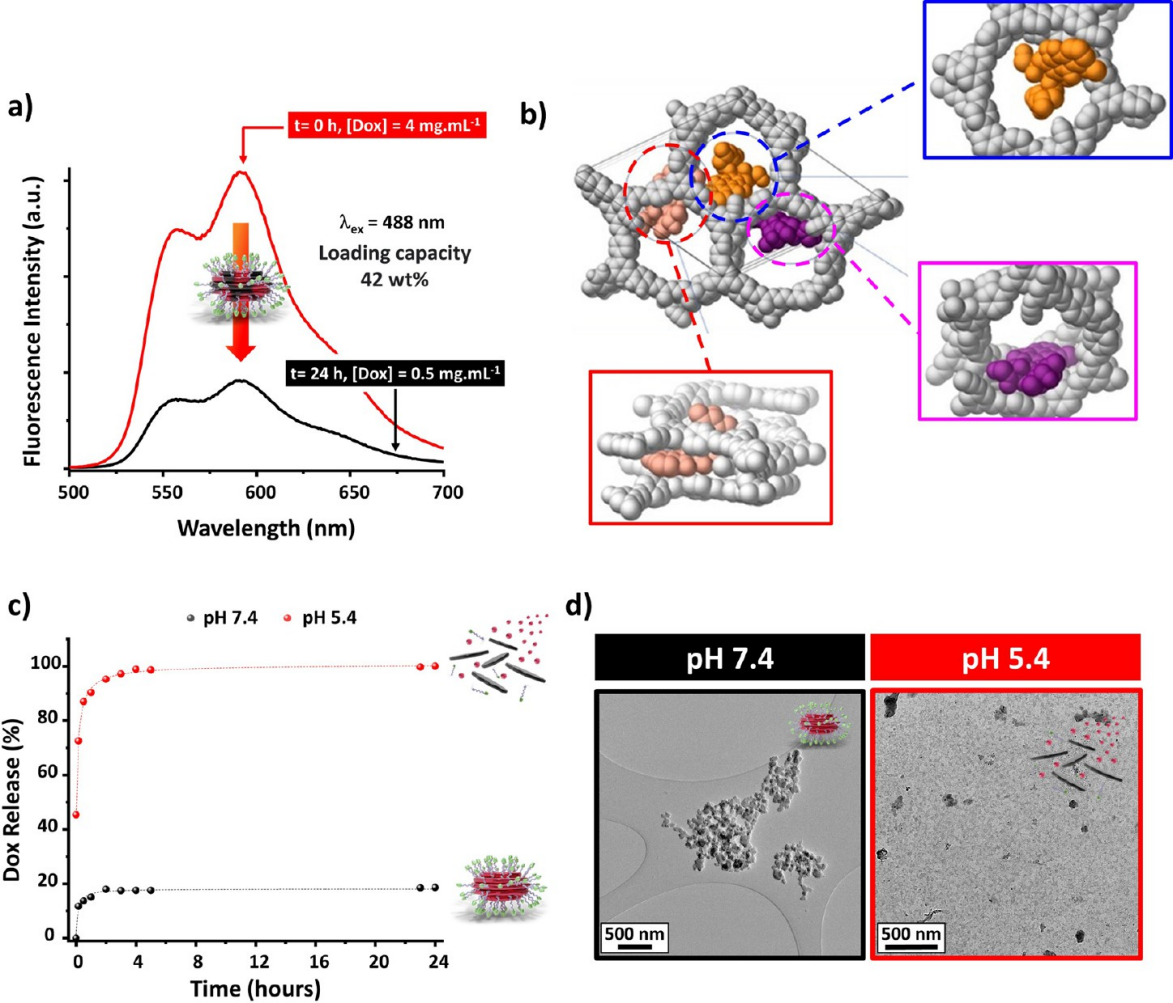
Dox loading in Alkyn-nCOF-cRGD and its pH-dependent release. (a) Fluorescence emission spectra of a diluted supernatant solution containing Dox before (red curve) and 24 h after the addition of Alkyn-nCOF-cRGD (black curve). Dox fluorescence intensity was used to derive the loading efficiency (λ_ex_ = 488 nm, H_2_0, pH 7.4, 298 K). The experiment was performed in triplicate. (b) Schematic diagram displaying three Dox molecules occupying a pore within Alkyn-nCOF. The nCOF pores are occupied by Dox molecules depicted in orange and purple, while an additional Dox molecule, shown in salmon, adopts a sandwich-like conformation nestled between the layers of nCOF. (c) In vitro Dox release from Alkyn-nCOF-cRGD at 37 °C in 10 mM PBS solution at pH 7.4 (black curve) or 5.4 (red curve). (d) TEM images of Alkyn-nCOF-cRGD@Dox after 24 h at pH 7.4 (black) and 5.4 (red).

The anticancer drug Dox was loaded in Alkyn-nCOF and Alkyn-nCOF-cRGD by impregnation at pH 7.4 and 37 °C for 24 h (weight ratio Alkyn-nCOF/Dox = 1:5). Using fluorescence spectroscopy of the Dox-containing supernatant solution, the loading efficiency of Alkyn-nCOF and Alkyn-nCOF-cRGD was calculated to be 61 ± 4, and 42 ± 3 wt %, respectively (Figures 4a and S37), which are among the highest values compared to existing COF systems (Supporting Information Table S4 for comparison with reported COFs). At the same Dox concentration, an aqueous sample of free Dox shows greater fluorescence intensity than Alkyn-nCOF@Dox and Alkyn-nCOF-cRGD@Dox, indicating fluorescence quenching of Dox within the NPs (Figure S25). This quenching can be attributed to electronic interactions between the excited drug molecules and the nCOF pores or to self-quenching of the drug in the pores of the particle, where the effective concentration of the drug is relatively high.^47^ These results suggest that Dox molecules reside within the nCOF pores.^48−51^ The small dynamic radius and aromatic rings of Dox interact with Alkyn-nCOF through π–π conjugation. Additionally, the –NH and –OH groups of Dox can form hydrogen bonds with the framework, providing strong physical adsorption forces for drug loading and retention.^48−50,52,53^ Indeed, a simulated annealing process performed with three Dox molecules per Alkyn-nCOF unit cell (corresponding to a loading of about 50 wt %) shows different preferential sorption sites (Figure 4b). Thus, the Dox molecules fill the nCOF pores (orange- and purple molecules in Figure 4b). Interestingly, a sandwich conformation for a Dox molecule is also found between the nCOF layers (the salmon-colored molecule in Figure 4b). This behavior is possible due to the particular distorted hexagonal pore geometry of Alkyn-nCOF, which allows the diffusion of Dox through the pores into the interlayer space. Van Der Waals interactions between nCOF layers and Dox molecules, which also involve alkyne groups, are present in all cases.

The ^13^C CP/MAS NMR spectrum of Dox is well resolved (Figure S6), and shows peaks derived from aliphatic (10 to 100 ppm), aromatic (110 to 165 ppm), and carbonyl (180 to 190 ppm) carbons. The successful loading of Alkyn-nCOF with Dox was confirmed by the presence of a distinct peak of the carbonyl carbons of Dox at around 187 ppm in addition to the peaks of the Alkyn-nCOF (Figure S6). The presence of Dox molecules within the COFs was further confirmed by the sorption isotherm of the Alkyn-nCOF@Dox and Alkyn-nCOF-cRGD@Dox, which showed a decrease in BET surface area to 78 and 46 m^2^ g^–1^, a pore size distribution with an average pore width of 11.7 Å, and a total pore volume of 0.045 cm^3^ g^–1^ (Figure S19), indicating localization of Dox in the internal pores of the nCOFs. The PXRD peak intensity of the Alkyn-nCOF@Dox and Alkyn-nCOF-cRGD@Dox decreased significantly after Dox loading, indicating effective encapsulation of Dox in the nCOF pores (Figure S10).

The drug release behavior of Alkyn-nCOF@Dox and Alkyn-nCOF-cRGD@Dox was thoroughly investigated using Dox fluorescence in condition that simulate the slightly acidic pH of the lysosomal compartments and the variable pH within the tumor environment at 37 °C, using an in vitro dialysis method (Figures 4c and S38, S39). At a physiological pH of 7.4, less than 15% of the drug was released from both Alkyn-nCOF and Alkyn-nCOF-cRGD. This highlights the efficient entrapment of Dox in the nCOF pores and demonstrates the robust structural and drug-loading stabilities under these conditions. At a mildly acidic pH of 6.4, a significant yet controlled release of Dox was observed. However, at more acidic pH values of 4.0 and 5.4, a rapid and complete release of Dox occurred within 24 h. This confirms the expected pH sensitivity of the NPs and their ability to respond effectively to highly acidic environments.

Structural integrity assessments at pH 7.4 showed no significant changes in the morphology of Alkyn-nCOF@Dox and Alkyn-nCOF-cRGD@Dox over a 24 h period, as confirmed by TEM images and DLS measurements (Figures 4d, S16, and S32). At a pH of 6.4, the NPs maintained a size of approximately 60 nm, indicating relative stability even under mildly acidic conditions (Figure S32). At higher acidic pH (4.0 and 5.4), significant degradation occurred in both systems, characterized by a complete loss of structural morphology and a significant reduction in particle size (Figure S32). When tested in cell culture medium and FBS at a neutral pH of 7.4, the NPs showed sustained stability, with no significant morphological changes observed within 24 h (Figure S33). This sustained stability in neutral pH environments such as cell culture medium and FBS is critical to prevent premature release of the drug during circulation and ensures that the NPs can achieve targeted delivery in the tumor microenvironment.

The stability and functionality of Alkyn-nCOF-cRGD NPs are significantly impacted by the acidic conditions characteristic of the tumor microenvironment. These COFs are equipped with imine linkages that tend to hydrolyze in acidic environments and convert back into their constituent components. This reaction facilitates pH-dependent disintegration and thus enables the targeted release of drugs.^10^ The rate of imine hydrolysis, which is determined by chemical equilibrium and kinetics, increases with increased acidity, promoting the release of encapsulated Dox. Structural analyses indicate that while the COFs maintain their integrity at physiological pH, they readily disintegrate under acidic conditions. In addition, the acidic conditions in the tumor microenvironment likely disrupt the interactions (π–π conjugation and hydrogen bonding) that initially facilitate Dox loading, leading to the release of the drug from the NPs.^10^ This selective disintegration and bond disruption optimize drug delivery directly to the tumor sites while minimizing the impact on healthy tissue.

Taken together, these results indicate that the NPs we developed are potential candidates for the specific release of Dox in an acidic environment of tumor tissues coupled with peptide-targeting properties.

### 3.3 Comparative In Vitro Efficacy and Cytotoxicity Studies on Breast Cancer Cell Lines

Breast cancer is a complex and heterogeneous disease.^54^ Two representative cell lines corresponding to different breast cancer subtypes, with different aggressiveness, physiology, and treatment responses, were selected (Figure S40). MCF-7 cells originate from breast adenocarcinoma and typify as luminal breast cancers, characterized by a less aggressive nature. In contrast, MDA-MB-231 cells, known for their cancer stem-like features, fall under the category of triple-negative basal-type breast cancer. They are notorious for their aggressive behavior and pose a challenging prognosis due to higher instances of treatment resistance.^55^ The aggressive behavior of MDA-MB-231 cells is linked to their elevated expression of α_v_β_3_ integrin. This integrin plays a crucial role in promoting tumor growth and invasiveness and contributes to the increased migratory and metastatic capabilities of these cancer cells.^56,57^ However, α_V_β_3_ integrins are only weakly expressed on MCF-7.^56,57^ Therefore, MDA-MB-231 cells were used as integrin-positive cancer cells overexpressing α_v_β_3_ integrin on the cell membrane. In contrast, MCF-7 cells were chosen as a negative control for low expressed α_v_β_3_ integrin. Based on the interaction between cyclic RGD peptides and α_v_β_3_ integrins, we compared in vitro the ability of Alkyn-nCOF and Alkyn-nCOF-cRGD to internalize and release Dox in both cell lines. The biological activity of Alkyn-nCOF and Alkyn-nCOF-cRGD were compared in vitro and in vivo, with both systems loaded with the same amount of Dox (40 wt %).

#### 3.3.1 Evaluation of Cytotoxicity and Cellular Response to Treatment

**Figure 5:**
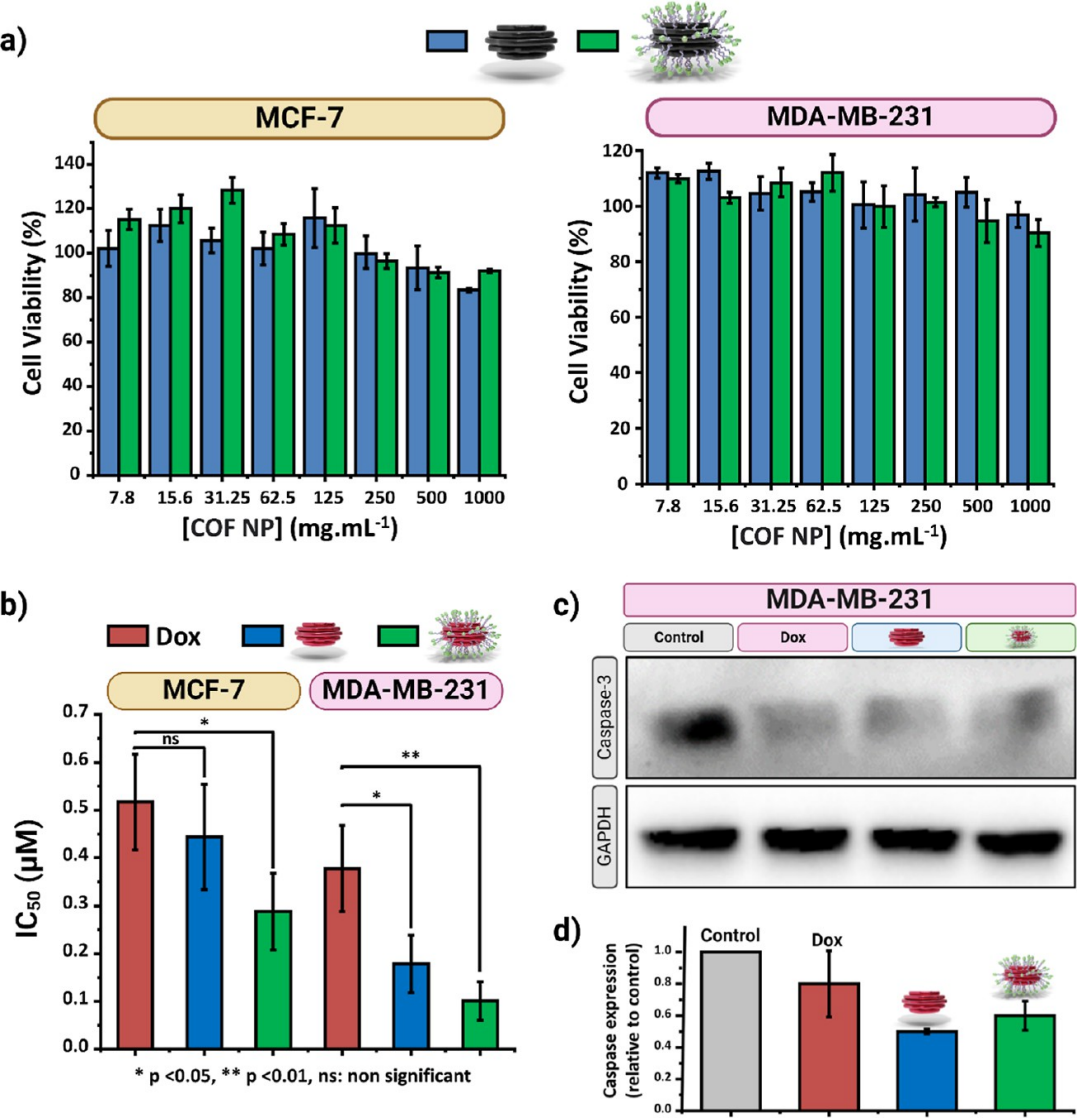
In vitro antitumor study in breast cancer cells with high (MDA-MB-231) and low (MCF-7) integrins levels. (a) Cell viability of MCF-7 and MDA-MB-231 after 48 h treatment with Alkyn-nCOF (blue) and Alkyn-nCOF-cRGD (green) at concentrations up to 1 mg/mL. Error bars denote the standard deviations from triplicate measurements. (b) Cytotoxic effects (IC_50_ values) of free Dox (red), Alkyn-nCOF@Dox (blue), and Alkyn-nCOF-cRGD@Dox (green) on MCF-7 and MDA-MB-231 cells. Data represent the mean ± SD from three independent experiments. Statistical significance was assessed using Student’s *t*-test, where ns denotes not significant, *p < 0.05, and **p < 0.01, when compared to the Dox-treated group. (c) Western blot analysis illustrating Caspase-3 protein expression of MDA-MB-231 cells treated for 24 h with no additives (control, gray), free Dox (red), Alkyn-nCOF@Dox (blue), and Alkyn-nCOF-cRGD@Dox (green) at a Dox concentration of 1 μM. (d) Quantitative analysis of normalized Caspase-3 expression relative to control in MDA-MB-231 cells treated for 24 h with free Dox (red), Alkyn-nCOF@Dox (blue), and Alkyn-nCOF-cRGD@Dox (green) at a Dox concentration of 1 μM. Error bars denote standard deviations illustrating the variability within duplicate experiments.

To evaluate the biocompatibility of Alkyn-nCOF and Alkyn-nCOF-cRGD (without drug), in vitro viability studies were performed on MCF-7 and MDA-MB-231 cells (Figure 5a). Alkyn-nCOF and Alkyn-nCOF-cRGD did not elicit cytotoxic effects at concentrations up to 1 mg mL^–1^ and after 48 h of incubation. These in vitro results are indicative of biocompatibility.

Next, we investigated the anticancer effects of three different Dox formulations—free Dox, Alkyn-nCOF@Dox, and Alkyn-nCOF-cRGD@Dox—on MCF-7 and MDA-MB-231 cells (Figures 5b and S41). In the case of MCF-7 cells, both the free Dox (IC_50_ = 0.52 ± 0.1 μM)^58^ and Alkyn-nCOF@Dox (IC_50_ = 0.44 ± 0.1 μM) showed comparable IC_50_ values. However, the Alkyn-nCOF-cRGD@Dox proved to be significantly more potent (IC_50_ = 0.28 ± 0.09 μM, *p* ≤ 0.05 compared to Dox). In MDA-MB-231 cells, Alkyn-nCOF-cRGD@Dox again proved to be the most effective formulation (IC_50_ = 0.10 ± 0.03 μM), showing significant superiority (*p* < 0.01 compared to Dox) over both free Dox (IC_50_ = 0.39 ± 0.09 μM)^59^ and Alkyn-nCOF@Dox (IC_50_ = 0.18 ± 0.1 μM). The increased potency of Alkyn-nCOF-cRGD@Dox, particularly pronounced in MDA-MB-231 cells, could be ascribed to its potential enhanced uptake by integrin α_v_β_3_ receptor-mediated endocytosis leading to increased cytotoxicity of Dox due to its targeted delivery. This observation prompted us to further investigate the cell death and internalization mechanism of Dox, Alkyn-nCOF@Dox, and Alkyn-nCOF-cRGD@Dox in MDA-MB-231 cells compared to MCF-7 cells.

The Caspase family is essential in mediating apoptotic responses, with Caspase-3 being a key pro-apoptotic protease.^60^ To investigate whether cell death in MCF-7 and MDA-MB-231 cells treated with Alkyn-nCOF@Dox and Alkyn-nCOF-cRGD@Dox results from apoptosis via caspase cascade activation, we conducted Western blot experiments to measure Caspase-3 protein levels. These experiments compared the effects of these compounds to those induced by free Dox, as shown in Figure 5c. In MCF-7 cells, Caspase-3 was not detectable, which is consistent with previous findings indicating the absence of Caspase-3 protein expression in these cells (Figure S42).^61^ In contrast, the Western Blot data for MDA-MB-231 cells, presented in Figure 5c,d, reveal a notable decrease in pro-Caspase-3 levels (32 kDa) following treatment with Dox, Alkyn-nCOF@Dox, and Alkyn-nCOF-cRGD@Dox. This decline points to apoptosis, likely driven by alterations in the caspase pathways, as a primary cell death mechanism in MDA-MB-231 cells after these treatments.

#### 3.3.2 Examination of Intracellular Distribution and Endocytic Mechanisms

The interaction between NPs and cells is influenced by the surface properties of the NPs.^62^ Cell uptake occurs primarily by endocytosis, either by nonspecific macropinocytosis or through a targeted approach using receptor-mediated endocytosis.^63^ When NPs are equipped with specific ligands such as the cRGD peptide, they induce targeted integrin-mediated endocytosis, mainly through the clathrin-mediated pathway.^64,65^ After internalization, the NPs generally move to endosomes and then to lysosomes for degradation, with behavior possibly dependent on factors such as the nature of NPs, ligands, and receptor expression levels of the cell.^64,65^ Therefore, we investigated the interaction and possible endocytic pathways of Alkyn-nCOF@Dox and Alkyn-nCOF-cRGD@Dox in MCF-7 and MDA-MB-231 cells at the ultrastructural level using TEM. We qualitatively examined endocytosis pathways in normally functioning cells without using specific inhibitors to avoid potential disruptions that inhibitors might cause. Although several cytotoxicity studies have been performed with drug-loaded COF NPs,^48−50^ none has directly visualized their interactions with cell membranes and organelles.

**Figure 6:**
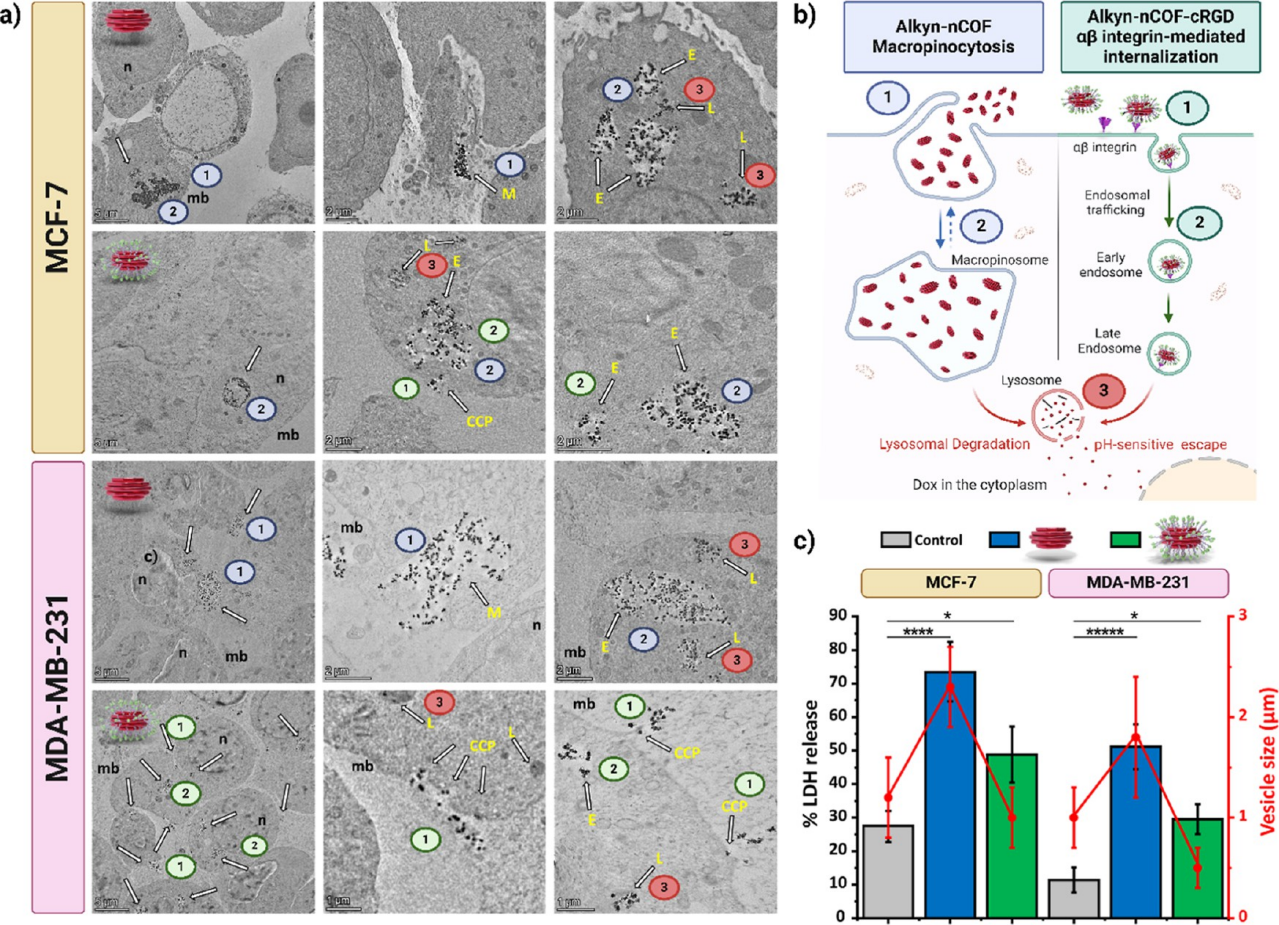
Differential cellular responses to Alkyn-nCOF and Alkyn-nCOF-cRGD NPs in MCF-7 and MDA-MB-231 cells: analysis of membrane interaction and internalization. (a) High-resolution electron micrographs comparing cellular interactions in MCF-7 (upper panel) and MDA-MB-231 (lower panel) cells when exposed to Alkyn-nCOF@Dox and Alkyn-nCOF-cRGD@Dox for 4 h. White arrows pinpoint internalized NPs after 4 h incubation. Key identifiers: *n* = nucleus; mb = membrane; M = macropinosome; CCP = clathrin-coated pits; E = endosome; L = lysosome. (b) Schematic representations detailing the mechanisms of macropinocytosis (left, blue) and αβ integrin-mediated internalization (right, green). The journey of the compounds within the cells is depicted through sequential stages, from the formation of macropinosomes (left, blue) or clathrin-coated pits (right, green) to their eventual lysosomal degradation and pH-sensitive release into the cytoplasm. Corresponding numbers on the micrographs indicate these stages. (c) Comparison of endocytotic vesicle size measured by TEM and percentage (%) LDH release in breast cancer cell lines MCF-7 and MDA-MB-231 after a 24 h treatment period. The cells, characterized by low (MCF-7) and high (MDA-MB-231) integrin levels, were exposed to a concentration of [Dox] = 10 μM of Alkyn-nCOF@Dox (blue) and Alkyn-nCOF-cRGD@Dox (green), with gray bars indicating the control cells without treatment. The *y*-axis on the right (red) represents the vesicle size in micrometers (μm), while the *y*-axis on the left (black) indicates the % of LDH release, a marker of cell membrane integrity. The red lines with corresponding data point markers provide a visual guide for interpreting changes in vesicle size across the different treatments and cell types. Error bars denote standard deviations. Statistical significance was assessed using Student’s *t*-test, where *p < 0.05; **p < 0.01; ***p < 0.001; ****p < 0.0005; *****p < 0.0001 when compared to the control sample.

In MCF-7 cells, large aggregates of both Alkyn-nCOF@Dox and Alkyn-nCOF-cRGD@Dox were detected near or under membrane protrusions and within large vacuoles in the cytoplasm. These aggregates occurred mainly in the perinuclear region, suggesting macropinocytosis as a likely pathway for NP internalization, with the macropinosomes’ endocytic vesicles varying in size and shape and sometimes reaching substantial diameters, 2.3 ± 0.4 μM for Alkyn-nCOF@Dox and 1.0 ± 0.3 μM for Alkyn-nCOF-cRGD@Dox (Figures 6a,b, S43–S47 and S53) whereas MCF-7 control cells display vesicle size of 1.2 ± 0.4 μM.^64,65^ However, MCF-7 cells treated with Alkyn-nCOF-cRGD@Dox have fewer particles interacting directly with the cell membrane. This observation suggests some degree of receptor-mediated endocytosis, which is consistent with the low levels of integrin in MCF-7 cells (Figures 6a,b and S43–S47).^63,65^ The distribution of vesicle size in the cells is shown in Figure S53.

In MDA-MB-231 cells, significant aggregates of Alkyn-nCOF@Dox are primarily detected near the membrane, where membrane ruffling is also observed. These aggregates accumulate in the perinuclear region within large vacuoles, resulting in increased cellular uptake by macropinocytosis (1.8 ± 0.6 μM whereas MDA-MB-231 control cells 1.0 ± 0.3 μM). This uptake mode appears to be receptor-independent, which was also observed in MCF-7 cells (Figures 6a,b, and S48–S52).^66,67^ MDA-MB-231 cells treated with Alkyn-nCOF-cRGD@Dox showed an increased number of smaller endocytic vesicles (0.5 ± 0.2 μM) which contained fewer particle aggregates than cells treated with nontargeted NPs. The Alkyn-nCOF-cRGD@Dox particles mainly bind to the cell membrane individually and enhanced uptake into cancer cells through receptor-mediated endocytosis, such as clathrin-mediated pathways (Figures 6a,b, and S48–S52). NP aggregates were observed in clathrin-coated pits on the plasma membrane and in internalized clathrin-coated vesicles, confirming this endocytic pathway. Alkyn-nCOF-cRGD@Dox-bound integrins are internalized and then transported to endosomes and lysosomes (Figures 6a,b and S48–S52).^64^ The Alkyn-nCOF-cRGD@Dox NPs adhere to the vesicle wall, unlike Alkyn-nCOF@Dox NPs, which are evenly distributed in endosomes. Individual NPs were also observed exiting the vesicles and residing in the cytosol. Our results emphasize that adding the cRGD peptide to surface of the NPs alters the uptake mechanism and cellular processing in MDAMB-231 cells with high integrin levels. This alteration modifies the entry pathway, and directs it in particular through receptor-mediated endocytosis, such as clathrin-mediated cycles.

The extracellular release of lactic acid dehydrogenase (LDH) is an established marker of cell membrane integrity. Elevated LDH levels in the extracellular environment typically indicate the cell membrane injury, which can be caused by various factors, including the interaction with exogenous agents like NPs.^68−70^ Since LDH is predominantly localized in the cytoplasm, its detection outside the cell is a clear sign of membrane permeabilization or injury.^68−70^ To assess the extent of cell membrane damage in breast cancer cells with varying levels of integrin expression, we evaluated the release of LDH from MCF-7 and MDA-MB-231 cells after 24 h treatment with Alkyn-nCOF@Dox and Alkyn-nCOF-cRGD@Dox, both at a Dox equivalent concentration of 10 μM (Figure 6c).

In MCF-7 cells, a significant increase in LDH release was noted upon treatment with Alkyn-nCOF@Dox. This increase was quantified at 73.4 ± 8.7%, showing a statistically significant difference (*p* ≤ 0.0001) compared to untreated control cells, which showed a 27.5 ± 4%. In contrast, cells treated with Alkyn-nCOF-cRGD@Dox exhibited a moderate LDH release of 48.8 ± 8.3%, which was still statistically significant (*p* ≤ 0.05) compared to the control. This suggests that the larger vesicle size associated with Alkyn-nCOF@Dox treatment (≈2.3 ± 0.2 μM) could be a factor in the impaired membrane integrity. Conversely, when treated with Alkyn-nCOF-cRGD@Dox, the vesicle size was smaller (≈1.0 ± 0.3 μM), indicative of a degree of receptor-mediated endocytosis that may mitigate extensive membrane damage, as shown by the two vesicle size distributions (Figure S53).

In MDA-MB-231 cells, treatment with Alkyn-nCOF@Dox led to a markedly higher release of LDH (51.2 ± 6.7%, *p* ≤ 0.000001) compared to control cells, which released 11.4 ± 3.8% LDH. This was significantly greater than the LDH release observed with Alkyn-nCOF-cRGD@Dox treatment, which was 29.5 ± 4.5% (*p* ≤ 0.05 compared to control). This suggests that Alkyn-nCOF@Dox treatment causes significant damage to the cell membrane and can be correlated with TEM images, showing larger endocytic vesicles with an average size of approximately 1.8 μm (±0.6), suggesting that membrane distortion could lead to the formation of membrane defects and subsequent LDH release.^68−70^ In contrast, MDA-MB-231 cells treated with the targeted Alkyn-nCOF-cRGD@Dox presented smaller endocytic vesicles, of approximately 0.5 μm (±0.2), containing fewer NP aggregates. This indicates less membrane distortion and, thus, a lower extent of LDH release than in cells treated with nontargeted NPs.^68−70^

The molecular properties of the cell have a major impact on the cellular response to NP treatment, as shown by the different LDH release and vesicle size in MCF-7 and MDA-MB-231 cells. In addition, the targeted approach of Alkyn-nCOF-cRGD@Dox appears to reduce nonspecific cellular interactions, resulting in less membrane damage. This suggests that receptor-targeted NPs can offer a more refined treatment strategy that takes advantage of the unique molecular signatures of cancer cells, potentially leading to better therapeutic outcomes with less collateral damage to the cells.

**Figure 7:**
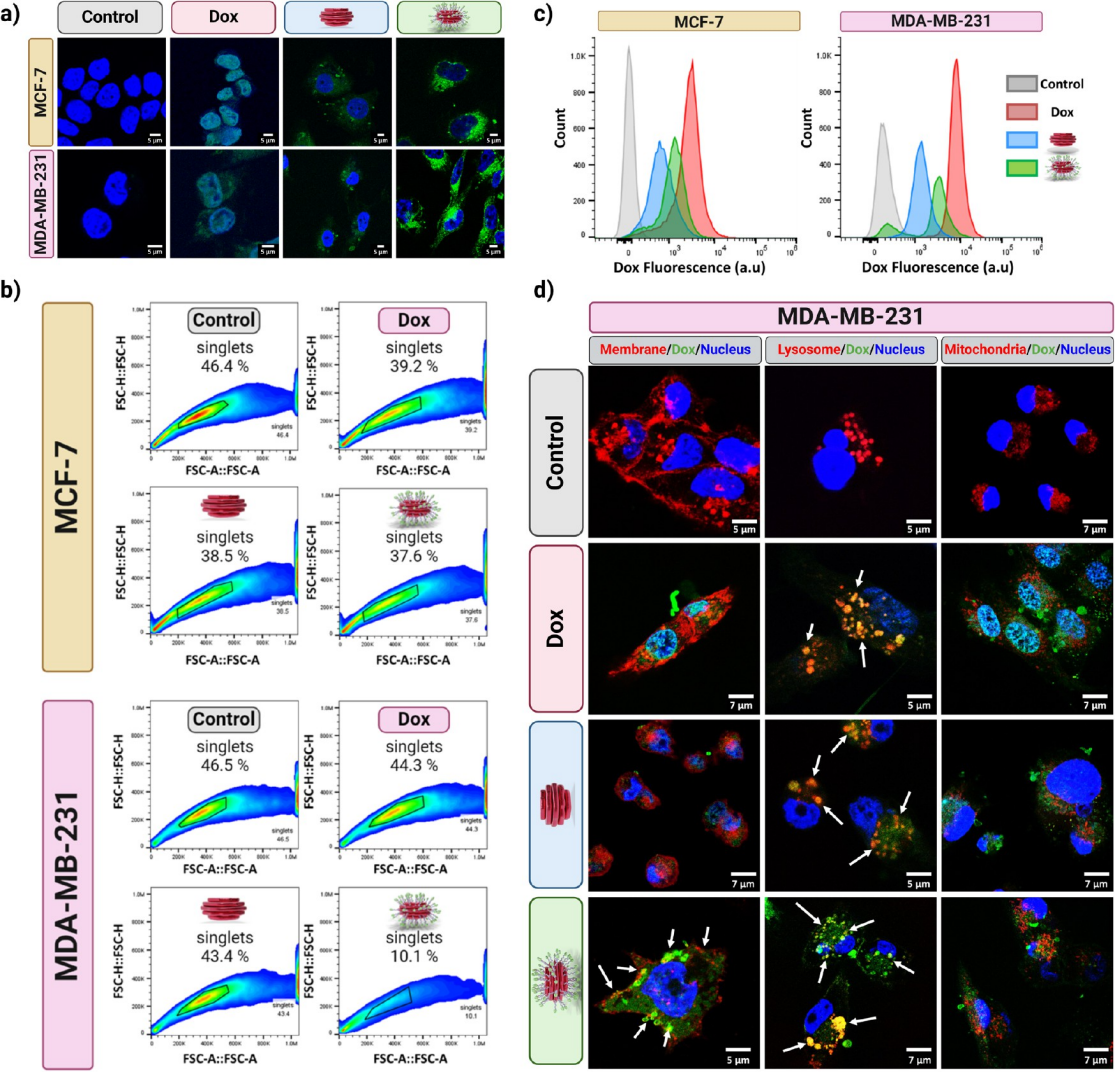
Intracellular trafficking of Dox in responses to Alkyn-nCOF and Alkyn-nCOF-cRGD NPs in MCF-7 and MDA-MB-231 cells. (a) CLSM images displaying MCF-7 (top panel) and MDA-MB-231 cells (bottom panel) subjected to a 24 h treatment with control (no additive), free Dox, Alkyn-nCOF@Dox, or Alkyn-nCOF-cRGD@Dox at a concentration of [Dox] = 10 μM. Dox fluorescence was captured with 488 nm excitation/520 nm emission, while nuclear DAPI staining was visualized under 355 nm excitation and 450 nm emission. (b) Flow cytometry analysis of MCF-7 and MDA-MB-231 cell populations after 24 h of treatment ([Dox] = 1 μM), using forward scatter area (FSC-A) and forward scatter height (FSC-H) gating: the depicted charts show the percentages of single-cell events (singlets) in untreated controls compared to cells treated with Dox, Alkyn-nCOF@Dox, and Alkyn-nCOF-cRGD@Dox. Notably, MDA-MB-231 cells exhibit a marked reduction in singlets when treated with Alkyn-nCOF-cRGD@Dox, indicating a potential increase in cell aggregation or cell death. (c) Flow cytometry analysis of Dox fluorescence within MCF-7 (left) and MDA-MB-231 (right) cells post 24 h incubation with either no additive (control, gray), free Dox (red), Alkyn-nCOF@Dox (blue), or Alkyn-nCOF-cRGD@Dox (green) at a concentration of [Dox] = 1 μM. (d) CLSM images of MDA-MB-231 cells after 4 h of treatment with no additive (control), free Dox, Alkyn-nCOF@Dox, or Alkyn-nCOF-cRGD@Dox at [Dox] = 10 μM, and costained with red markers for the plasma membrane, lysosomes, and mitochondria. All images include nuclear DAPI labeling. Channels used: DAPI (λ_ex_ = 400 nm), Dox (λ_ex_ = 488 nm), and red marker (λ_ex_ = 647 nm).

The intracellular internalization of free Dox, Alkyn-nCOF@Dox, and Alkyn-nCOF-cRGD@Dox was further detected by confocal laser scanning microscopy (CLSM) and flow cytometry. MDA-MB-231 and MCF-7 breast cancer cells were incubated with free Dox, Alkyn-nCOF@Dox, and Alkyn-nCOF-cRGD@Dox ([Dox] = 10 μM) for 24 h. Internalization of Dox was then visualized using CLSM at Dox excitation (λ_ex_ = 488 nm/λ_em_ = 520 nm) (Figures 7a and S54–S55). The nucleus was also specifically stained using DAPI (λ_ex_ = 360 nm/λ_em_ = 460 nm). Free Dox was predominantly found in the nuclei of both cell types, where it intercalates with DNA strands, causing chromatin condensation and apoptosis.^71,72^ In contrast, the fluorescence from Alkyn-nCOF@Dox and Alkyn-nCOF-cRGD@Dox was primarily in the cytoplasm, Alkyn-nCOF-cRGD@Dox showed higher fluorescence intensity in the cytoplasm in all cell types compared to its nontargeting counterpart confirming efficient receptor-mediated endocytosis.

Flow cytometry was used to evaluate the levels of intracellular Dox in MDA-MB-231 and MCF-7 cells after 24 h of treatment with: free Dox, Alkyn-nCOF@Dox, and Alkyn-nCOF-cRGD@Dox, at a concentration of 1 μM (Figures 7b,c and S56). To ensure accurate measurement of intracellular Dox concentrations, single cells were differentiated from cell debris, doublets, or clusters by gating on FSC-A with FSC-H, indicative of cell size and morphology, respectively.

Population analysis of single cells revealed a 78.2% decrease in singlets in MDA-MB-231 cells after 24 h of exposure to Alkyn-nCOF-cRGD@Dox, compared to the control group (with singlets at 46.5% for control and 10.1% for treated cells, as shown in Figure 7b). Conversely, the singlets population in MCF-7 cells treated with Alkyn-nCOF-cRGD@Dox experienced a modest 18.9% reduction compared to the control (46.4% singlets in control versus 37.6% in treated cells, shown in Figure 7b). Treatments with Dox and Alkyn-nCOF@Dox had negligible impact on the single cell populations of both MCF-7 and MDA-MB-231 cells. These results are consistent with the previously reported toxicity profile (Figure 5b), and emphasize the significant toxicity of Alkyn-nCOF-cRGD@Dox in MDA-MB-231 cells.

Quantitative analysis of Dox fluorescence in single cells indicated that MCF-7 and MDA-MB-231 cells treated with Dox exhibited a fluorescence signal 40 times greater than that of the control cells (Figures 7c and S56). For cells treated with Alkyn-nCOF@Dox and Alkyn-nCOF-cRGD@Dox, the fluorescence intensity increased by 10.1 and 18.8-fold in MCF-7 cells and by 7.1 and 16.1-fold in MDA-MB-231 cells, respectively, compared to untreated cells.

These results suggest that the incorporation of the RGD peptide on the NPs effectively doubles the release of Dox into the cells. The comparatively higher fluorescence signal of cells treated with free Dox compared to cells treated with Alkyn-nCOF-cRGD@Dox could be due to the greater cell mortality induced by the latter, as indicated by cytotoxicity assessments and the FSC-A vs FSC-H analysis (Figure 7b). It is, therefore, likely that only the cells that internalized a lower amount of Dox from the NPs survived and were thus detected in the fluorescence analysis.

To determine the subcellular localization of the Dox formulations, MDA-MB-231 cells were treated for 4 h with free Dox, Alkyn-nCOF@Dox, or Alkyn-nCOF-cRGD@Dox and then stained with organelle-specific fluorescent markers (DAPI, LysoTracker 647 Deep Red, MitoTracker Deep Red FM, and CellMask 647 Deep Red to visualize nuclei, lysosomes and late endosomes, mitochondria and membrane, respectively) followed by CLSM imaging (Figure 7d). Four hours after treatment, free Dox was colocalized with lysosomes, late endosomes, and nuclei in MDA-MB-231 cells (Figure 7d). Dox was shown to enter primarily through passive diffusion and endocytosis, potentially leading to lysosomal capture.^71−74^ Regardless of the uptake mechanism, its main target is the nucleus, where it interferes with DNA functionality.^71,72,74^ Alkyn-nCOF@Dox was also observed to colocalize with lysosomes and late endosomes but not with mitochondria or nuclei. Alkyn-nCOF-cRGD@Dox colocalized with the cell membrane, confirming receptor-mediated endocytosis, as well as with lysosomes and late endosomes (Figure 7d). We also detected increased fluorescence in the cytoplasm. This enhanced cytoplasmic fluorescence can be attributed in part to the release of Dox from the NPs and in part to the increased number of internalized NPs resulting from endosome fusion and lysosomal acidification (Figure 7d). These observations suggest that cellular internalization of Alkyn-nCOF-cRGD@Dox’s into the cytoplasm is facilitated by receptor-mediated endocytosis, which triggers Dox release into the cytosol. These findings are consistent with the TEM, flow cytometry, CLSM, and toxicity results.

The therapeutic efficacy and targeting properties of Alkyn-nCOF-cRGD@Dox in vitro encouraged us to further investigate its antitumor ability in vivo in mice bearing triple-negative MDA-MB-231 breast tumors.

### 3.4 In Vivo Biological Studies

#### 3.4.1 Assessment of NP Biodistribution in Major Organs of Athymic NU/J Nude Mice

**Figure 8:**
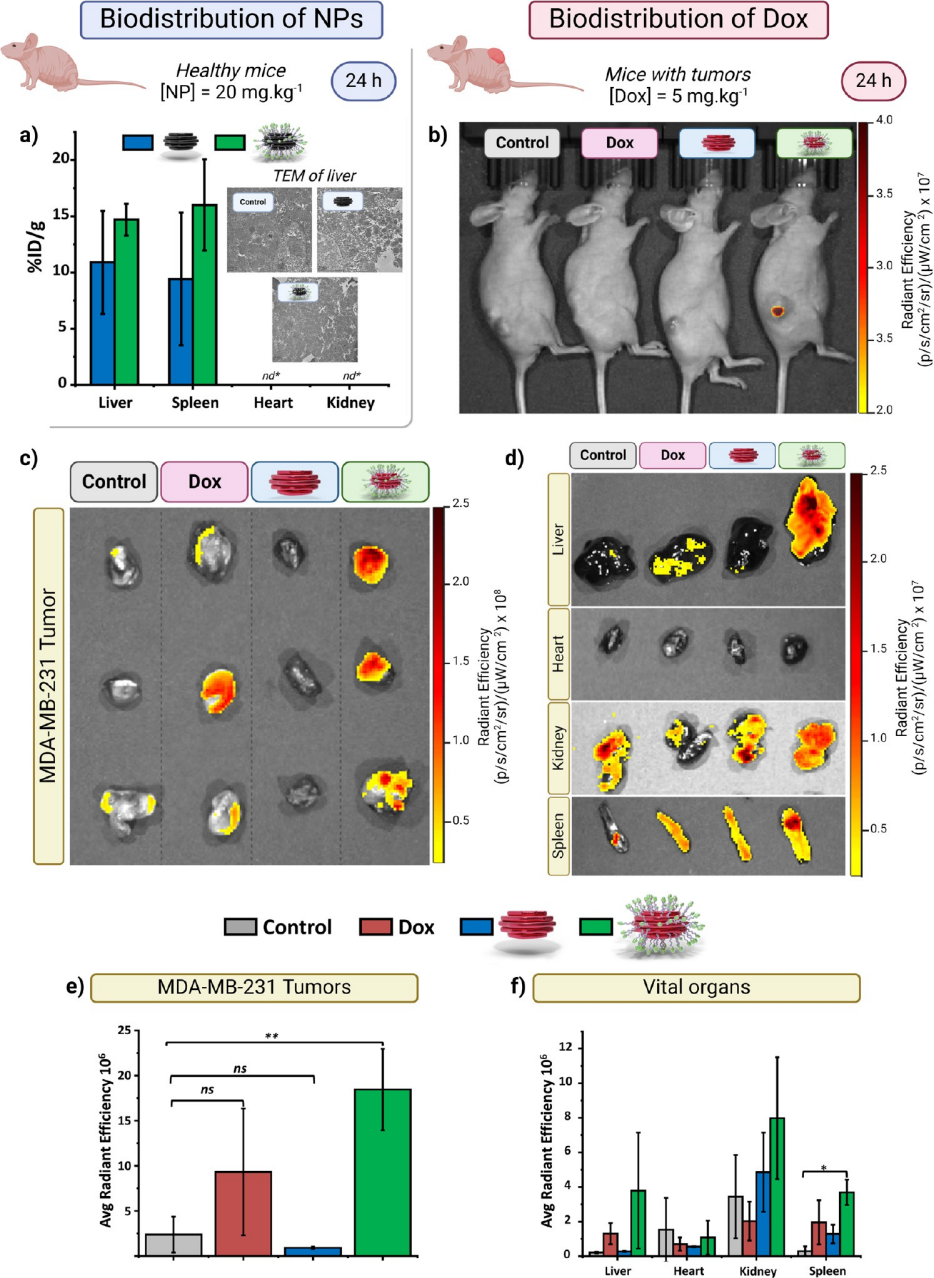
Assessment of NP distribution in healthy and tumor-bearing mice. (a) Biodistribution profile of nondrug-loaded NPs in major organs (heart, liver, spleen, kidney) following intraperitoneal administration in healthy mice. Sacrifice and quantitative analysis were performed 24 h postadministration of Alkyn-nCOF (blue bars) and Alkyn-nCOF-cRGD (green bars) NPs. Dose: [NP] = 20 mg/kg, 200 μL, with three mice per group (*n* = 3). The inset shows representative TEM images of liver sections from treated and control mice, illustrating the maintenance of normal liver architecture. nd* = nondetected. (b) In vivo fluorescence imaging (IVIS spectrum) of tumor-bearing mice under anesthesia, depicting the distribution of Dox fluorescence 24 h after treatment with PBS (control), free Dox, Alkyn-nCOF@Dox, or Alkyn-nCOF-cRGD@Dox. Dose: [Dox] = 5 mg/kg, [NP] = 10 mg/kg, 200 μL. Ex vivo fluorescence images using IVIS spectrum (c,d) and quantitative analysis of Dox radiance efficiency (e,f) of excised tumors (c,e) and vital organs, including liver, heart, kidney, and spleen (d,f) from the same groups of mice as in panel (b), showing fluorescence intensity indicative of Dox distribution within the tissues. Organs were dissected and analyzed via IVIS spectrum for fluorescence imaging, 24 h postinjection. Each bar represents one of the treatments: control (gray), free Dox (red), Alkyn-nCOF@Dox (blue), and Alkyn-nCOF-cRGD@Dox (green). Values are mean ± SD (*n* = 3), ns: nonsignificant, *p ≤ 0.05, **p ≤ 0.01 compared to the control sample.

We initially investigated the immediate biodistribution of NPs in athymic NU/J nude mice by assessing the percentage of the injected dose (ID) per gram of tissue. We administered both drug-free Alkyn-nCOF and Alkyn-nCOF-cRGD intraperitoneally (IP) at a concentration of 20 mg/kg body weight. This dosage is double that intended for subsequent toxicity studies (Figure 8a). Twenty-four hours postadministration, the mice were euthanized, and pivotal organs were harvested. We specifically focused on the liver, spleen, and kidneys, anticipating them to be primary clearance organs that might exhibit cellular responses to NP accumulation. Additionally, the heart was scrutinized for any signs of cardiotoxicity.^75^ Following a thorough rinse with PBS, organs were homogenized and subsequently subjected to UV–vis spectrometry for NP detection. Both Alkyn-nCOF and Alkyn-nCOF-cRGD were well-tolerated by the mice, with no discernible weight loss noted (Figure S57). Notably, approximately 11 and 9% of ID of Alkyn-nCOF and 15 and 16% of ID of Alkyn-nCOF-cRGD localized in the liver and the spleen, respectively, with no apparent accumulation in the heart or kidneys, implying effective circulatory clearance, in line with previous studies.^76^ TEM imaging confirmed the presence of NPs in the liver and spleen but also validated the absence of any structural alterations (Figures 8a inset and S58).

To assess the tumor-targeting potential of Alkyn-nCOF-cRGD@Dox, we employed real-time IVIS spectrum imaging system and subsequent Dox radiance efficiency quantification. Athymic NU/J nude mice carrying subcutaneous MDA-MB-231 tumors (50–100 mm^3^) received IP injections of Alkyn-nCOF-cRGD@Dox, Alkyn-nCOF@Dox, Dox, or a saline solution ([Dox] = 5 mg/kg, [NP] = 10 mg/kg). Animals were anesthetized, and whole-body dorsal fluorescence images were captured 24 h postinjection. Live animal imaging, as shown in Figure 8b, indicated an increase in fluorescence intensity only in the tumor of the animal treated with Alkyn-nCOF-cRGD@Dox, highlighting the targeted delivery facilitated by the cRGD conjugation.

Then, we euthanized the mice to harvest the tumors and vital organs, including the heart, liver, spleen, and kidney. We conducted a detailed examination of Dox’s biodistribution by performing ex vivo IVIS spectrum imaging and quantifying Dox fluorescence. As shown in Figure 8c,e, a significant increase in fluorescence was noted in the tumors from mice treated with Alkyn-nCOF-cRGD@Dox. This contrasted the moderate fluorescence observed in the free Dox group, underscoring the enhanced tumor-targeted delivery capabilities of the cRGD-conjugated NPs. We also analyzed the general biodistribution of the Dox treatments (Figure 8d,f). Organ-specific investigation indicated that the injected Alkyn-nCOF-cRGD was predominantly localized in the liver, with detectable fluorescence also present in the spleen and kidneys, as depicted in Figure 8d. The distribution pattern of Dox across the liver, spleen, and kidneys suggests efficient systemic clearance of the Alkyn-nCOF-cRGD that did not localize within the tumor.^77^

These results underscore the precision targeting of Alkyn-nCOF-cRGD@Dox NPs, their favorable tumor retention, and their effective systemic elimination. This promising profile motivates further investigation of their antitumor effects on MDA-MB-231 tumor-bearing mice.

#### 3.4.2 Comparative Analysis of In Vivo Antitumor Efficacy

**Figure 9:**
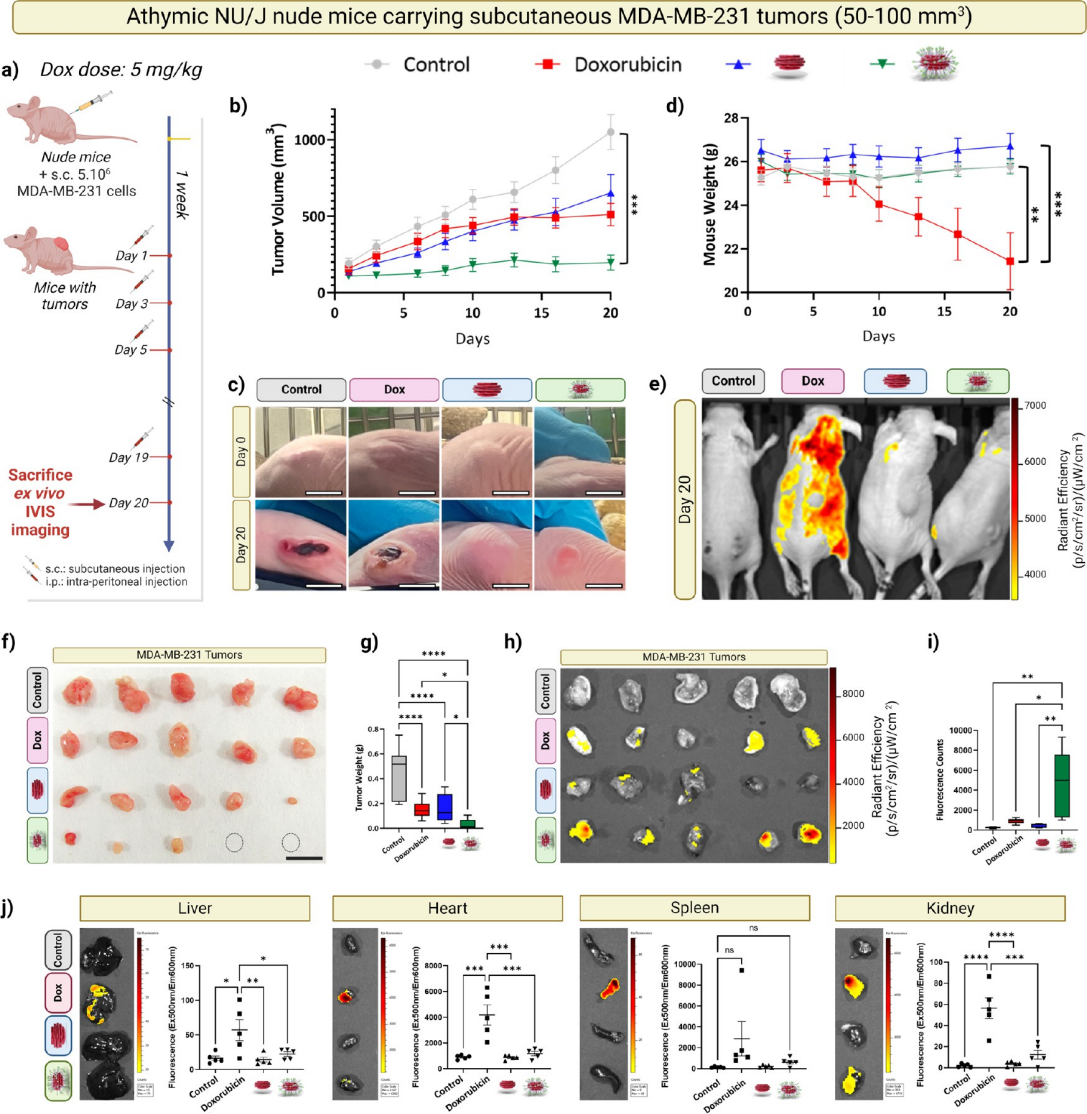
Assessment of antitumor efficacy in athymic NU/J nude mice with MDA-MB-231 tumors. (a) Schematic of the treatment and evaluation timeline. (b) Tumor volume progression in mice after administration of PBS (control, *n* = 11, gray), free Dox (*n* = 15, red), Alkyn-nCOF@Dox (*n* = 14, blue), and Alkyn-nCOF-cRGD@Dox (*n* = 14, green), with a Dox dose of 5 mg/kg. (c) Visual comparison of tumor in mice at the start (day 0) and after 20 days of treatment. (d) Body weight tracking across different treatment groups throughout the 20 day treatment. (e) IVIS spectrum fluorescence imaging depicting Dox distribution in anesthetized mice after 20 day treatment. (f) Ex vivo images of excised tumors at the end of the treatment, with scale bars representing 1 cm. (g) Post-treatment tumor weight analysis. (h) IVIS spectrum fluorescence imaging of the harvested tumors, and (i) quantification of Dox radiance efficiency among different treatment groups after treatment. (*n* = 5). (j) Ex vivo evaluation of Dox distribution in the liver, heart, spleen, and kidneys of tumor-bearing nude mice treated with PBS (control), Dox alone, Alkyn-nCOF@Dox, and Alkyn-nCOF-cRGD@Dox on day 20, including average ROI measurements. (*n* = 5) statistical significance was determined using one-way ANOVA with Tukey’s post hoc test. Significance denoted as: *p < 0.05; **p < 0.01; ***p < 0.001; ****p < 0.0005.

Next, we compared the anticancer therapeutic effects of Alkyn-nCOF-cRGD@Dox, Alkyn-nCOF@Dox, and free Dox in an orthotopic tumor model. Athymic NU/J nude mice with subcutaneous MDA-MB-231 tumors (50–100 mm^3^) were IP injected every 2 days with Alkyn-nCOF-cRGD@Dox, Alkyn-nCOF@Dox, Dox, or saline solution as control ([Dox] = 5 mg kg^–1^ [NP] = 10 mg kg^–1^, 200 μL, Figure 9a). The selected doses of Dox (5 mg kg^–1^) and NPs (10 mg kg^–1^) in a 200 μL volume were based on established therapeutic norms for TNBC models and preliminary toxicity studies, that balance anticancer efficacy and safety, ensure consistent drug delivery, and allow effective tumor targeting without significant systemic toxicity. The evolution of tumor volume shows that Alkyn-nCOF-cRGD@Dox treatment has significantly blocked the growth of tumors in mice (Figure 9b). A statistical analysis of these changes over the 20 day treatment period shows that only the injections of Alkyn-nCOF-cRGD@Dox had a significant effect on slowing tumor growth (*p* < 0.001) when compared to the control group (Figure 9b). In contrast, mice treated with Alkyn-nCOF@Dox and free Dox exhibited comparable and statistically insignificant level of tumor suppression compared to the control group. These results reflect the limited efficacy of nontargeted chemotherapy.

Figure 9c shows the progression of tumor size in mice in the four treatment groups at the start (day 0) and after 20 days. In the groups treated with PBS and free Dox, there was a noticeable increase in tumor size, accompanied by obvious necrosis at day 20. For the group treated with Alkyn-nCOF@Dox, tumors enlarged significantly without any signs of necrosis. Remarkably, in the Alkyn-nCOF-cRGD@Dox group, the tumors had almost disappeared by day 20, highlighting the potential efficacy of this targeted treatment approach.

Moreover, body weight analysis shows a significant reduction in the weight of mice treated with free Dox. This indicates that free Dox has considerable side effects, resulting in weight loss even as the tumor continues to grow. In contrast, the Alkyn-nCOF@Dox, Alkyn-nCOF-cRGD@Dox, and saline-treated groups showed no significant changes in body weight compared to the initial weights at the start of treatment. These results demonstrate that Alkyn-nCOF@Dox and Alkyn-nCOF-cRGD@Dox treatments have minimal side effects (Figure 9d). After the 20-day treatment regimen, whole-body dorsal fluorescence imaging was performed and provided explanation to the body weight results. Figure 9e illustrates a marked presence of off-target Dox fluorescence in the animals treated with free Dox. This explains the considerable weight loss in this group due to the free Dox systemic toxicity. This was in sharp contrast to the groups treated with Alkyn-nCOF-cRGD@Dox or Alkyn-nCOF@Dox, which exhibited significantly reduced off-target fluorescence when compared to the control.

At the end of the treatment, the mice were sacrificed, and the tumors and major organs were extracted. The visualization of the tumor and the weight analysis show drastic differences between the treatment groups (Figure 9f,g). The average tumor weight decreased by 67% in the free Dox group, by 65% in the Alkyn-nCOF@Dox group, and by a notable 95% reduction in the Alkyn-nCOF-cRGD@Dox group compared to the control group (Figure 9g). These results demonstrate that incorporating the RGD peptide significantly improves targeted tumor therapy and subsequent drug delivery in vivo.

Further analysis was conducted using IVIS spectrum through ex vivo fluorescence imaging of tumors that did not vanish during the treatment and vital organs (heart, liver, spleen, lung, and kidney). The findings corroborated our initial observations. Notably, tumors from the Alkyn-nCOF-cRGD@Dox-treated mice displayed fluorescence signals that were markedly higher than those from the saline, free Dox, and Alkyn-nCOF@Dox groups (Figure 9h,i). To quantify, the Dox fluorescence in tumors treated with Alkyn-nCOF-cRGD@Dox was 16.5-fold higher than the saline group, ∼5-fold higher than the free Dox group, and 11.2-fold higher than the Alkyn-nCOF@Dox group (Figure 9h,i). Furthermore, a significant fluorescence indicative of Dox was detected in the heart, spleen, liver, and kidneys of mice treated with free Dox, showcasing the drug’s nonselective distribution and its potential for unintended systemic toxicity (Figure 9j). In contrast, the organs harvested from mice treated with Alkyn-nCOF-cRGD@Dox or Alkyn-nCOF@Dox did not exhibit significant fluorescence when compared to the control (Figure 9j).

Overall, the in vivo results indicate that the targeted chemotherapeutic agent Alkyn-nCOF-cRGD@Dox, which utilizes the RGD peptide as a targeting agent, provides significantly improved treatment efficacy and specificity over conventional treatments with nontargeted Alkyn-nCOF@Dox and free Dox. The targeted approach not only more effectively suppresses tumor growth, but also minimizes side effects, as evidenced by stable body weight and reduced systemic toxicity. These results underline the potential of RGD-modified nCOF nanocarriers for more precise and safer chemotherapy.

## 4 Conclusion

In conclusion, this study demonstrates the remarkable potential of alkyne-functionalized nCOFs modified with cyclic RGD peptides for targeted drug delivery in TNBC. The Alkyn-nCOF-cRGD platforms showcase an innovative integration of COF stability and responsiveness to the acidic microenvironment of TNBC cells, which enables precise drug release mechanisms. This approach significantly improves the therapeutic efficacy and safety profile by minimizing systemic toxicity while maximizing drug concentration at the tumor site. The successful in vitro and in vivo results not only highlight the advanced targeting capabilities of these engineered nCOFs but also set new standards for personalized cancer therapy. By harnessing the unique properties of COFs and combining them with targeted delivery strategies, we are moving closer to developing treatments that are as precise and predictable as they are effective. The challenges now lie in scaling up alkyne-functionalized nCOFs for clinical use, particularly in ensuring reproducibility and cost efficiency, as well as navigating the complex regulatory landscape for clinical translation. The economic impact of production costs versus potential healthcare savings must also be carefully considered. In addition, the variability of α_v_β_3_ integrin expression and the limitations of TNBC models highlight the need for further research into the long-term stability and immunogenicity of nCOFs to fully realize their clinical potential.

## Supporting Information Available

Figures: S1–S65, Tables S1–S4, References (1–28) (PDF)
Movie S1: Variable-temperature liquid-cell transmission electron microscopy (VT-LCTEM) experiment: Movie 1 illustrates the in situ heating of TAB and Alkyn-DFP precursor solution to 110 °C in the presence of acetic acid and the formation of NPs (MP4)
