# Supplementary material for: cRGD-Peptide Modified Covalent Organic Frameworks for Precision Chemotherapy in Triple-Negative Breast Cancer
Source: ACS Appl Mater Interfaces. 2024 Sep 13;16(42):56676–95. doi: 10.1021/acsami.4c10812 (PMC11503616; doi:10.1021/acsami.4c10812)
Supplement: Supplementary file 1 — am4c10812_si_001.pdf [file am4c10812_si_001.pdf]

Supporting Information

cRGD-Peptide Modified Covalent Organic Frameworks for Precision Chemotherapy in Triple-Negative Breast Cancer

Farah Benyettou,[1*†] Mostafa Khair,[2†] Thirumurugan Prakasam,[1] Sabu Varghese,[2] Zineb Matouk,[3] Maryam Alkaabi,[1] Pilar Pena-Sánchez,[4] Maylis Boitet,[2] Rasha AbdulHalim,[1] Sudhir Kumar Sharma,[5] Rose Ghemrawi,[6] Sneha Thomas,[2] Jamie Whelan,[1] Renu Pasricha,[2] Ramesh Jagannathan,[5] Felipe Gándara,[4] Ali Trabolsi[1]*

Affiliations:

[1] Chemistry Program, New York University Abu Dhabi (NYUAD), Abu Dhabi 129188, United Arab Emirates

[2] Core Technology Platforms, New York University Abu Dhabi, 129188 Abu Dhabi, United Arab Emirates

[3] Technology Innovative Institute, Abu Dhabi 9639, United Arab Emirates.

[4] Instituto de Ciencia de Materiales de Madrid-CSIC, C. Sor Juana Inés de la Cruz 3, 28049 Madrid, Spain.

[5] Engineering Program, New York University Abu Dhabi (NYUAD), Abu Dhabi 129188, United Arab Emirates

[6] College of Pharmacy, Al Ain University; Abu Dhabi P.O. Box 112612, United Arab Emirates. AAU Health and Biomedical Research Center, Al Ain University, Abu Dhabi P.O. Box 112612, United Arab Emirates.

*Corresponding author. at105@nyu.edu, fb51@nyu.edu

† These authors contributed equally to this work

Contents

1 - General Materials and Methods 4

2. Synthesis 7

2.1. Synthesis of 4-ethynyl-2,6-diformyl pyridine (Alkyn-DFP) 7

2.2. Synthesis of Alkyn-nCOF 7

2.3. Synthesis of Alkyn-nCOF-cRGD 7

2.4. Dox loading in Alkyn-nCOF and Alkyn-nCOF-cRGD 8

2.5. *In vitro* Dox release. 8

3. Characterizations 9

3.1. Fourier Transform infrared (FTIR) spectroscopy 9

3.2. Solid-state NMR spectroscopy 12

3.3. X-ray photoelectron (XPS) spectroscopy 14

3.4. Powder X-ray diffraction (PXRD) 17

3.5. High-resolution transmission electron microscopy (HRTEM). 20

3.6. Atomic force microscopy (AFM) 27

3.7. N_2 adsorption-desorption experiments 29

3.8. UV-visible spectroscopy 31

3.9. Fluorescence spectroscopy 32

3.10. Thermogravimetric analysis (TGA) 34

3.11 Dynamic light scattering (DLS) 35

3.12. Quantification of cRGD-PEG-N_3 conjugated using the OPA method 41

3.13. Quantification of Dox loaded using fluorescence spectroscopy 44

3.14. pH-dependent Dox release experiments 47

4. *In vitro* biological studies 49

4.1. Cell culture 49

4.2. *In vitro* cell viability 49

4.3. *In vitro* cell toxicity 50

4.4. Western blotting 51

4.5. Intracellular distribution study using TEM 52

4.6. LDH release experiment 63

4.7. *In vitro* internalization study by confocal microscopy 63

4.8. Flow cytometry analysis 66

4.9. *In vitro* organelle co-localization study by confocal microscopy 67
5. *In vivo* biological studies 68
5.1. Biodistribution study on healthy animals 68
5.2. Tumor model establishment 70
5.3. Biodistribution study on tumor-bearing mice 70
5.4. *In vivo* antitumor efficacy 73
5.5. *In vivo* biodistribution study using IVIS Spectrum 74
5.6. Statistical Analysis. 77
References 78

1 - General Materials and Methods

All reagents and starting materials were purchased from Sigma-Aldrich and used without further purification. Deionized water was used from Millipore Gradient Milli-Q water purification system. Thin-layer chromatography (TLC) was performed on silica gel 60 F254 (E. Merck). The plates were inspected under UV light. Column chromatography was performed on silica gel 60F (Merck 9385, 0.040–0.063 mm). Infrared spectra were recorded on an Agilent Technologies Cary 600 Series FTIR Spectrometer using the ATR mode. The samples' PXRD patterns were recorded using an X-ray Panalytical Empyrean diffractometer. N_2 adsorption-desorption isotherms were obtained at 77 K using Micromeritics ASAP 2020 surface area analyzer. Transmission electron microscopy (TEM) images were obtained using a Thermo Fisher Scientific (TFS) Talos F200X scanning/transmission electron microscope (S/TEM) operating at 200 kV acceleration voltage. Samples for the TEM investigation were prepared by placing a 3μL drop of the NP suspension on a carbon-coated copper grid (TED PELLA, Inc.) and allowing the solvent to evaporate. The high-resolution images of periodic structures were analyzed using TIA software. The topography of the samples was analyzed by dynamic atomic force microscopy (5500 Atomic Force Microscope; Keysight Technologies Inc., Santa Rosa, CA). We acquired topography, phase, and amplitude scans simultaneously. Silicon cantilevers (NanosensorsTM, Neuchatel, Switzerland) with resonant frequencies of 250–300 kHz and force constants of 100–130 Nm^{-1} were used. The set point value was kept at 2.5V. AFM scans were collected at 1024 points/lines with a scan speed of 0.20 at a fixed scan angle of 0°. Scan artifacts were minimized by acquiring a typical scan at an angle of 90° under identical image acquisition parameters. We used GwyddionTM free software (version 2.47), an SPM data visualization and analysis tool for postprocessing the AFM scans. UV-visible absorption spectra were recorded with an Agilent Technologies Cary 5000 Series UV-Vis-NIR Spectrophotometer in water at room temperature (298 K). Solutions were examined in 1 cm spectrofluorimetric quartz cells. The experimental error of the wavelength values was estimated to be ~1 nm. Emission spectra in water at room temperature were recorded on a Perkin Elmer LS55 Fluorescence Spectrometer using an excitation wavelength of 488 nm, corresponding to the wavelength that absorbs maximally. Dynamic light scattering (DLS) measurements were performed on a Malvern Zetasizer NanoSeries to obtain the size and ζ-potential of the

nanoparticles. Magic Angle Spinning (MAS) solid-state NMR experiments were carried out on a Bruker Avance-HD 600 MHz spectrometer operating at a static field of 14.1 T using a 4.0 mm MAS probe. Dry samples were packed into 4.0 mm zirconia rotors and were spun at a MAS frequency of 14 kHz. ^{1}H-^{13}C CP/MAS experiments were performed using a standard linearly ramped cross-polarization pulse sequence. ^{13}C chemical shifts were externally referenced to the adamantane CH_2 signal at 38.48 ppm. All the NMR data were processed using TopSpin software. Thermogravimetric analysis (TGA) was performed on TA SDT Q600. The copper content of the nCOF was analyzed using the Inductively coupled plasma mass spectrometry (ICP-MS) on the Agilent 7800 series instrument after digesting the nCOF in nitric acid. Copper concentrations were determined by the external calibration method using copper calibration standard solutions in the 1–100 ppm range trace metal grade HNO_3. The digested nCOF samples were diluted in milliQ water to a final co100 to 200 ppm concentration measurements with no gas and helium mode. For The ICP-MS instrument control Agilent Mass Hunter software was used under the following conditions: RF power 1550 W, nebulizer gas flow 1.03 L/min, auxiliary gas flow 0.90 L/min, and plasma gas flow 15 L/min. Data were processed using Agilent Mass Hunter software. Phase contrast and fluorescence images were observed on an Olympus FV1000MPE confocal scanning microscope. Flow cytometry analyses were performed on Attune NxT Flow cytometer.

Table S1. Comparative analysis with other RGD-conjugated nanoparticle systems.

Nanoparticle System	Properties	Advantages	Limitations	Comparison with Alkyn-nCOF-cRGD@Dox
**Liposomes[1-2]**	Biocompatible, integrin-targeting	Enhanced tumor localization, reduced systemic toxicity	Lower stability and control over drug release	Alkyn-nCOF offers greater stability and controlled release triggered by tumor acidity, potentially improving therapeutic outcomes.
**Polymeric Nanoparticles[3-4] (e.g., PLGA)**	Controlled release, biodegradable	Versatility in drug loading and release kinetics, EPR effect	Variable degradation rates, potential for aggregation	nCOFs provide a higher degree of surface functionality and predictability in behavior, leading to better targeting efficiency and lower toxicity.
**Metallic Nanoparticles [5-6] (e.g., Gold, Silver)**	Photothermal properties, imaging capabilities	Useful for combined therapy and diagnostics, effective delivery	Long-term biocompatibility issues, potential toxicity	nCOFs are made from biocompatible organic materials that degrade into non-toxic byproducts, offering safer alternatives for therapeutic applications without heavy metal concerns.
**Silica-Based Nanoparticles[7-8]**	Good stability, biocompatible	Effective in stable drug encapsulation and delivery	Less tunable for responsive drug release in specific environments	nCOFs excel in tunability for degradation and responsive release in acidic tumor environments, enhancing drug delivery efficiency directly to cancer cells.

2. Synthesis

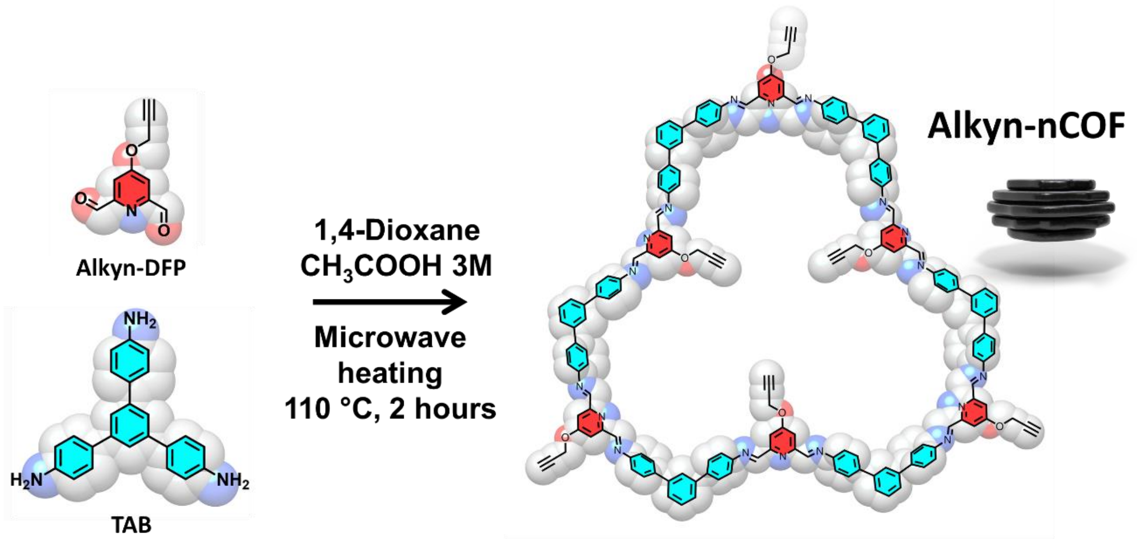

Figure S1. Chemical structure and synthetic route of Alkyn-nCOF obtained under microwave irradiation.

2.1. Synthesis of 4-ethynyl-2,6-diformyl pyridine (Alkyn-DFP)

4-ethynyl-2,6-diformyl pyridine synthesis was synthesized according to the published procedure with no modifications.[9]

2.2. Synthesis of Alkyn-nCOF

Alkyn-nCOF was synthesized by condensation of 4-ethynyl-2,6-diformyl pyridine (Alkyn-DFP, 12 mg, 0.06 mmol, 1 equiv.) and 1,3,5-tris(4-aminophenyl)benzene (TAB, 21 mg, 0.06 mmol, 1 equiv.) in anhydrous 1,4-dioxane (3 mL) with an acetic acid catalyst (0.5 mL, 3M) sealed in a 10 mL glass tube and heated under microwave irradiation to 110 °C for 120 minutes. The as-synthesized brownish gel was collected by filtration and washed with 1,4-dioxane and ethanol, followed by water.

2.3. Synthesis of Alkyn-nCOF-cRGD

The cRGD-PEG-N_3 (MW 1k, BOC Sciences) copper-catalyzed cycloadditions to the Alkyn-nCOF surface were carried out in water. Copper(II) sulfate (3.2 mg, 0.02mmol, 200 µL) and sodium

ascorbate (12 mg, 0.06 mmol, 200 µL) were added to the aqueous nCOF solution (10 mg, 2mL). The resulting brown solution was stirred (10 min) at room temperature, then the cRGD-PEG-N_3 azido reactant (10 mg, 100 µL) was added dropwise and stirred overnight. The solution was then cleaned with H_2O several times followed by centrifugation and finally by dialysis for 48 hours to remove unreacted species.

2.4. Dox loading in Alkyn-nCOF and Alkyn-nCOF-cRGD

The anticancer drug doxorubicin (Dox) was loaded into Alkyn-nCOF or Alkyn-nCOF-cRGD by a simple impregnation method. Alkyn-nCOF or Alkyn-nCOF-cRGD (10 mg) was suspended in an aqueous Dox solution at pH 7 ([Dox] = 0.4 mM, 10 mL, nCOF: Dox ratio = 1:5). The solution was placed under magnetic stirring for 24 hours. The solution was then cleaned with H_2O several times followed by centrifugation and finally by dialysis for 48 hours to remove unloaded Dox molecules. The Dox-loaded Alkyn-nCOF or Alkyn-nCOF-cRGD were denoted Alkyn-nCOF@Dox and Alkyn-nCOF-cRGD@Dox, respectively.

2.5. *In vitro* Dox release.

The kinetic of Dox release from Alkyn-nCOF@Dox and Alkyn-nCOF-cRGD@Dox was measured by fluorescence. Alkyn-nCOF@Dox or Alkyn-nCOF-cRGD@Dox (5 mL, 1.0 mg/mL) were placed into dialysis bags (MWCO: 3500 Da) and incubated at 37 °C in PBS solutions (30 mL) at pH 4.0, 5.4, 6.4 or 7.4 under gentle stirring. At regular intervals, 2 mL supernatant was collected and analyzed using fluorescence spectroscopy at Dox excitation. The kinetic curves of released Dox from Alkyn-nCOF or Alkyn-nCOF-cRGD were plotted over time; samples were run in triplicate.

3. Characterizations

3.1. Fourier Transform infrared (FTIR) spectroscopy

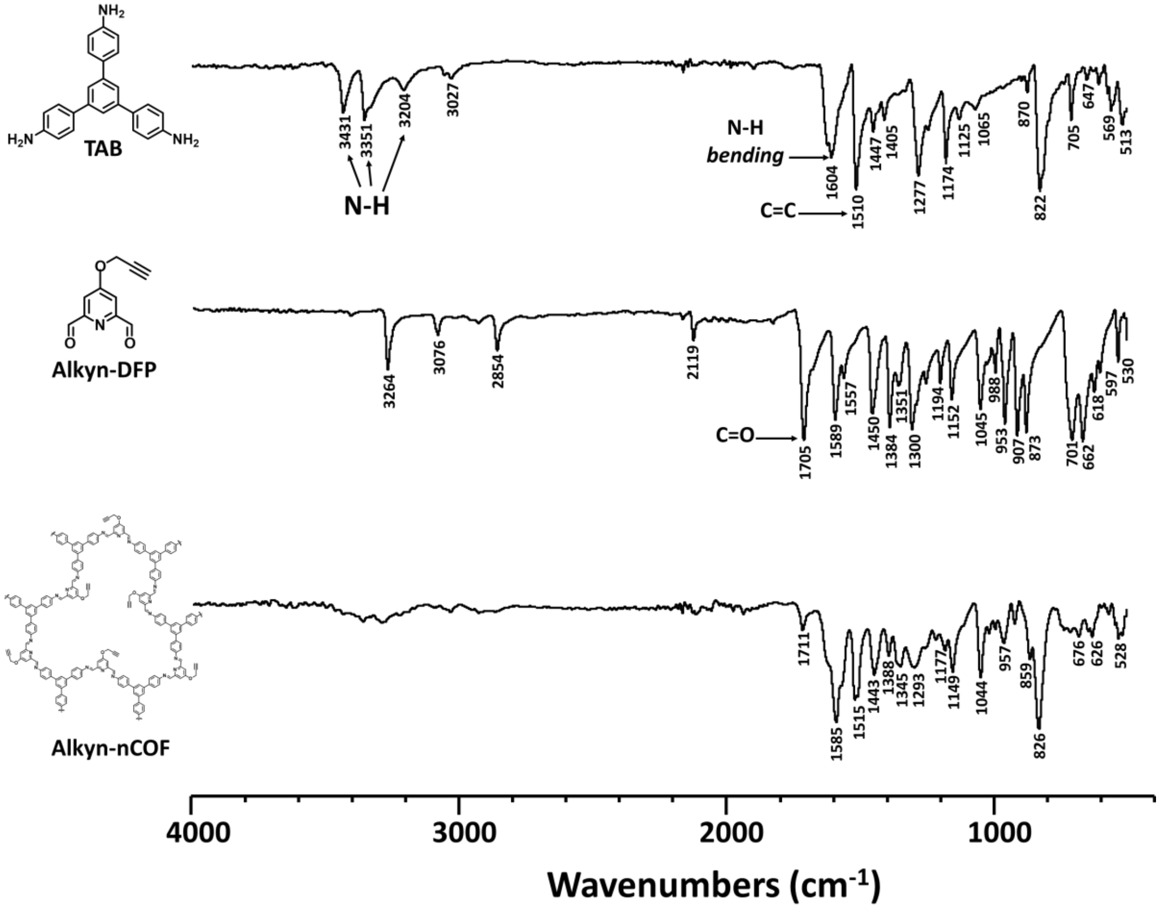

Figure S2. Stacked FTIR spectra of Alkyn-nCOF and its precursors, 4-ethynyl-2,6-diformyl pyridine (Alkyn-DFP) and 1,3,5-tris(4-aminophenyl)benzene (TAB).

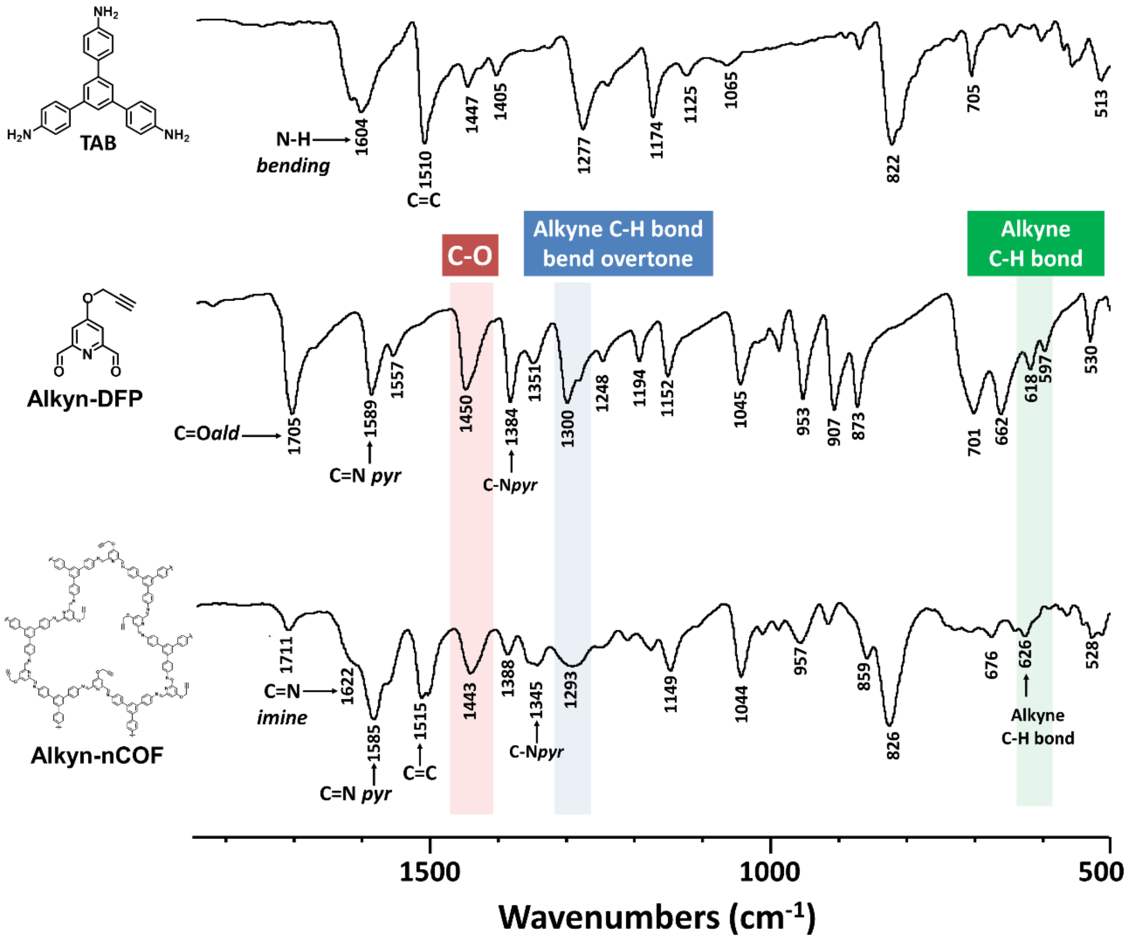

Figure S3. Stacked FTIR spectra of Alkyn-nCOF and its precursors, 4-ethynyl-1,6-diformyl pyridine (Alkyn-DFP) and 1,3,5-tris(4-aminophenyl)benzene (TAB) between 1800-500 cm^{-1}.

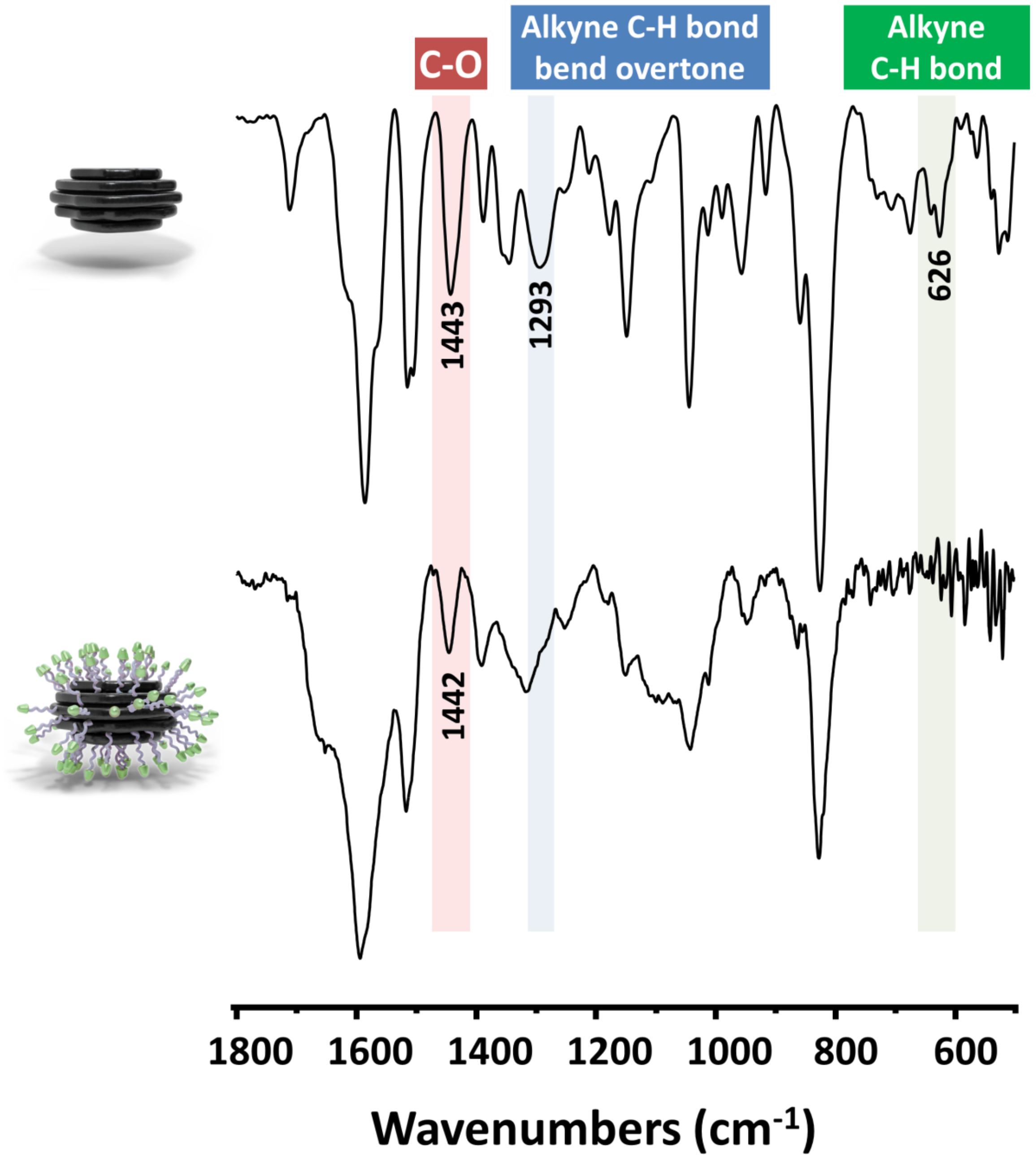

Figure S4. Stacked FTIR spectra of Alkyn-nCOF and Alkyn-nCOF-cRGD.

3.2. Solid-state NMR spectroscopy

a)

b)

c)

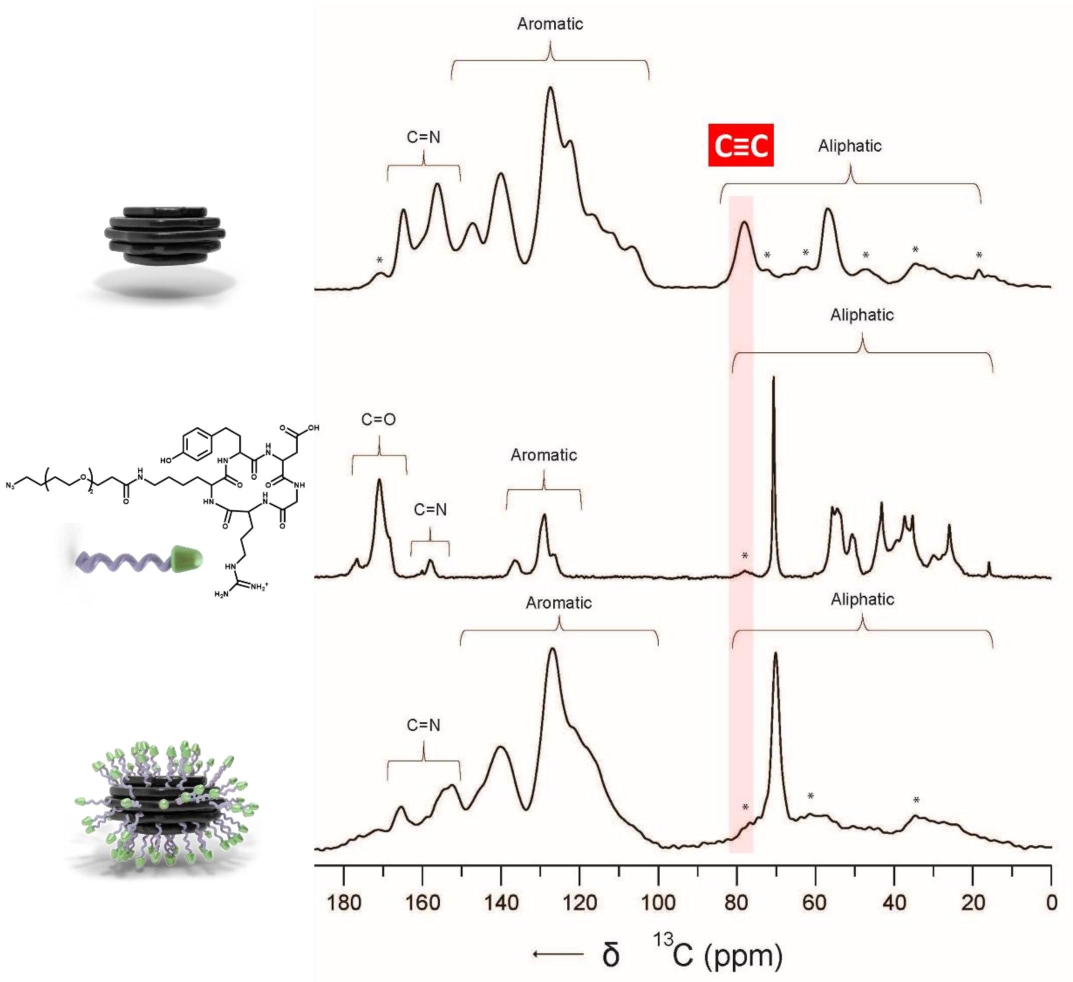

Figure S5. Stacked ^{13}C solid-state NMR spectra of a) Alkyn-nCOF, b) cRGD-PEG-N_3, and c) Alkyn-nCOF-cRGD. The asterisks denote spinning sidebands.

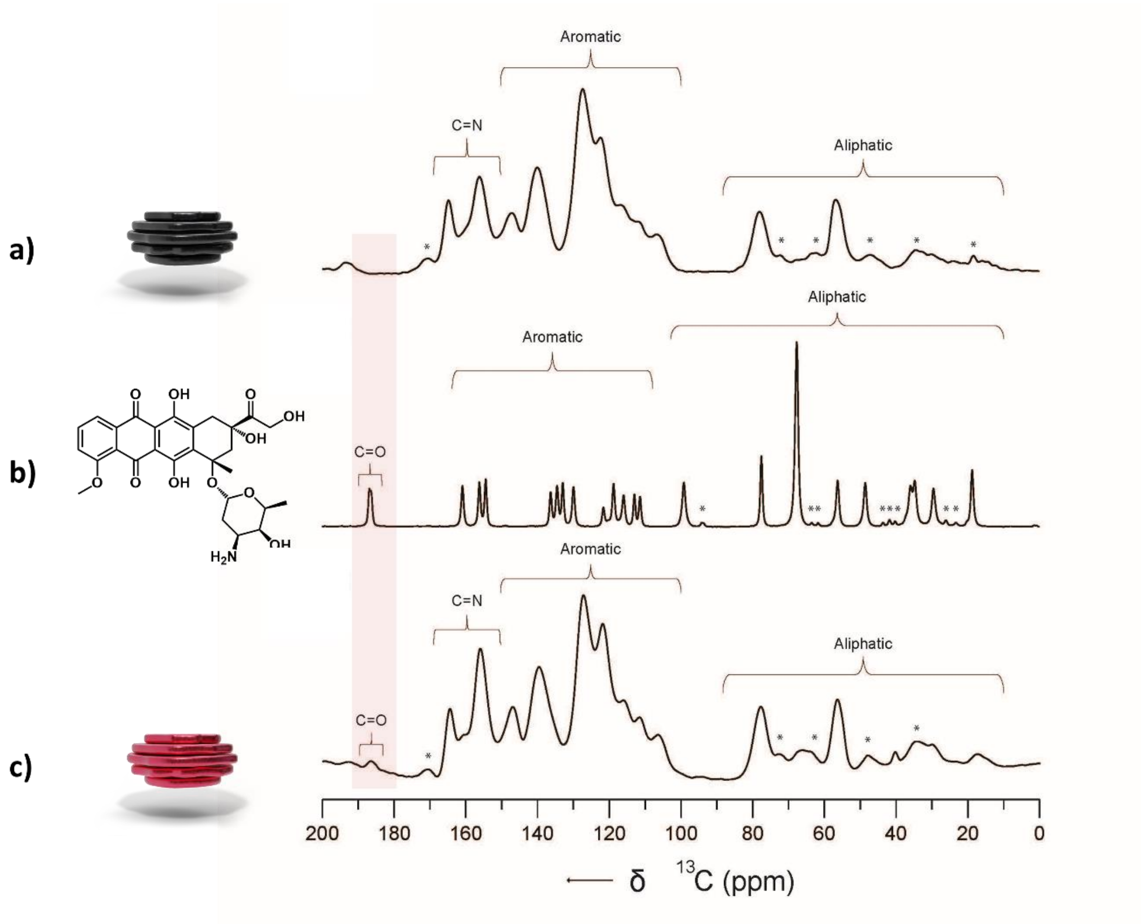

Figure S6. Stacked ^{13}C solid-state NMR spectra of a) Alkyn-nCOF, b) doxorubicin hydrochloride, and c) Alkyn-nCOF@Dox. The asterisks denote spinning sidebands.

3.3. X-ray photoelectron (XPS) spectroscopy

X-ray photoelectron spectroscopy (XPS) analysis was performed to analyse the elemental composition and understand the interactions between the nCOF surface and the peptide. XPS experiments were carried out on a Kratos Axis Ultra DLD spectrometer under a base pressure of ~ 2×10^{-10} mbar. A monochromated Al Kα X-ray source (1486.69 eV) irradiated samples at room temperature. XPS spectra were recorded from an analysis area of 700 µm × 300 µm. High-resolution XPS data of core levels were obtained with an energy resolution of 0.05 eV. For consistency, XPS measurements were calibrated to C 1s (~ 285 eV). Data were analyzed using CasaXPS package with Shirley background subtraction.

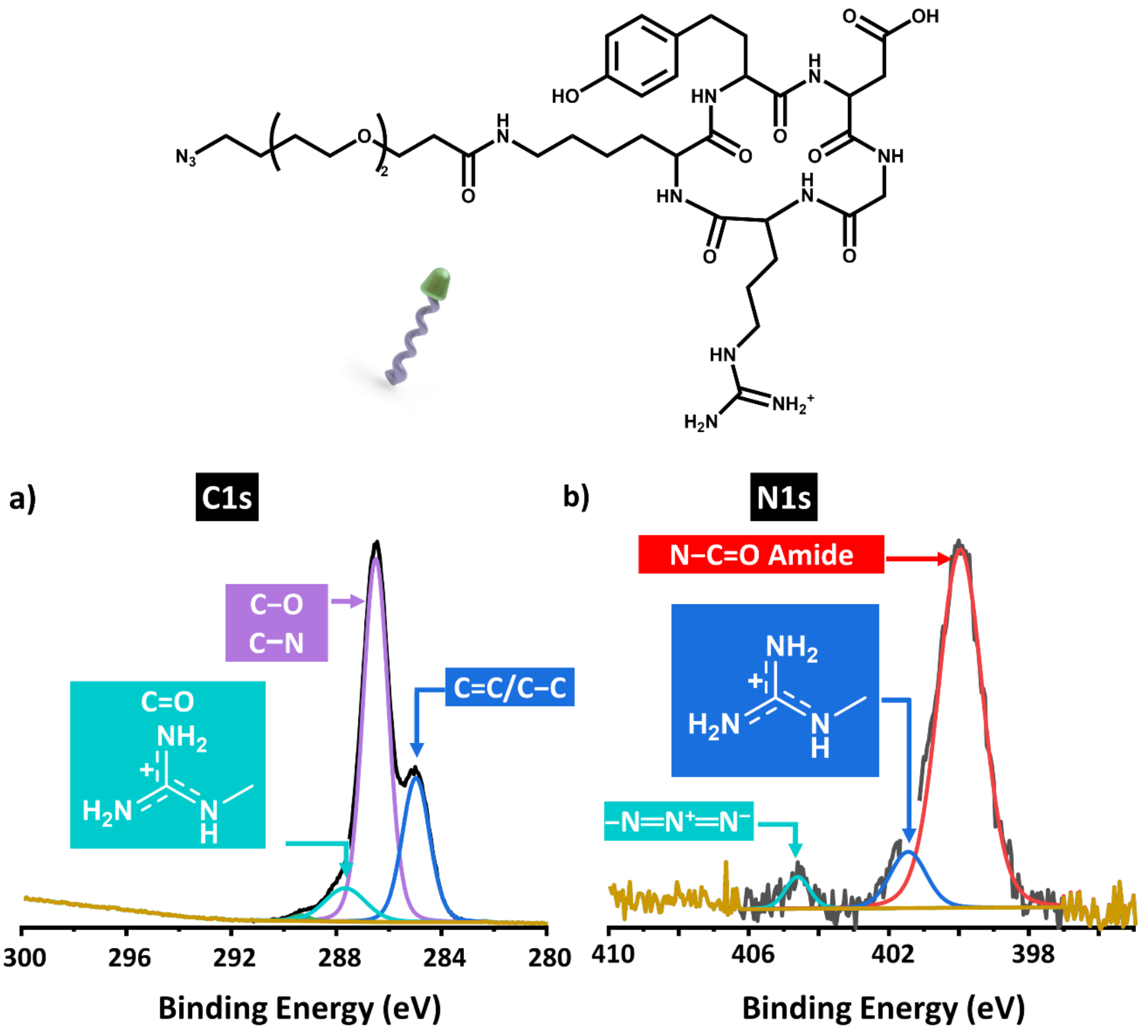

Figure S7. XPS a) C 1s and b) N 1s spectra of cRGD-PEG-N_3.

Table S2. Binding energy maxima, FHWM, Δ (eV) and % for C 1s spectrum deconvolution curves of Alkyn-nCOF, cRGD-PEG-N_3, and Alkyn-nCOF-cRGD.

			**Alkyn-nCOF**	**cRGD-PEG-N_3**	**Alkyn-nCOF-cRGD**
**C=C/C-C**	max		284.59	284.75	285.04
	FWHM	max	285.15	285.61	285.77
		min	284	284.3	284.33
		Δ	1.15	1.31	1.44
	%		28.5	27	40
**−C≡C-**	max		285.4	-	-
	FWHM	max	286.3	-	-
		min	284.6	-	-
		Δ	1.7	-	-
	%		36.6	-	-
**C-O / C-N**	max		286.79<br>*C-O / C-N=C /*<br>*C-N* pyridine	286.5<br>C-O / *N−C=O*	286.65<br>*C-O / C-N=C /*<br>*C-N* pyridine<br>*N−C=O*<br>*triazole*
	FWHM	max	288.14	287.05	287.64
		min	285.6	285.95	285.66
		Δ	2.54	1.1	1.98
	%		27.3	62	50
**-COOH guanidinium**	max		-	287.6	288.3
	FWHM	max	-	288.6	289.6
		min	-	286.8	287.2
		Δ	-	1.8	2.4
	%		-	9.5	9

Table S3. Binding energy maxima, FHWM, Δ (eV) and % for N 1s spectrum deconvolution curves of Alkyn-nCOF, cRGD-PEG-N_3, and Alkyn-nCOF-cRGD.

			**Alkyn-nCOF**	**cRGD-PEG-N_3**	**Alkyn-nCOF-cRGD**
**imine bond −N=C− amide N−C=O**	max		399.64 *imine bond −N=C−*	399.95 *amide N−C=O*	399.5 *imine bond −N=C− amide N−C=O*
	FWHM	max	400.31	400.72	400.93
		min	398.92	399.2	399.1
		Δ	1.3	1.52	1.83
	%		46.6	85	70
**N-pyridine guanidinium group −N= unit of the triazole**	max		400.49 *N-pyridine*	401.45 *guanidine group*	401.05 *N-pyridine guanidine group −N= unit of the triazole*
	FWHM	max	401.71	402.1	402.37
		min	399.23	400.76	399.77
		Δ	2.48	1.34	2.6
	%		53.4	10	30
**azide $N{=}N^{+}{=}N^{-}$**	max		-	404.6	-
	FWHM	max	-	405.08	-
		min	-	404.15	-
		Δ	-	0.93	-
	%		-	5	-

3.4. Powder X-ray diffraction (PXRD)

Powder X-ray diffraction (PXRD) measurements were carried out to confirm the crystalline nature of the framework.

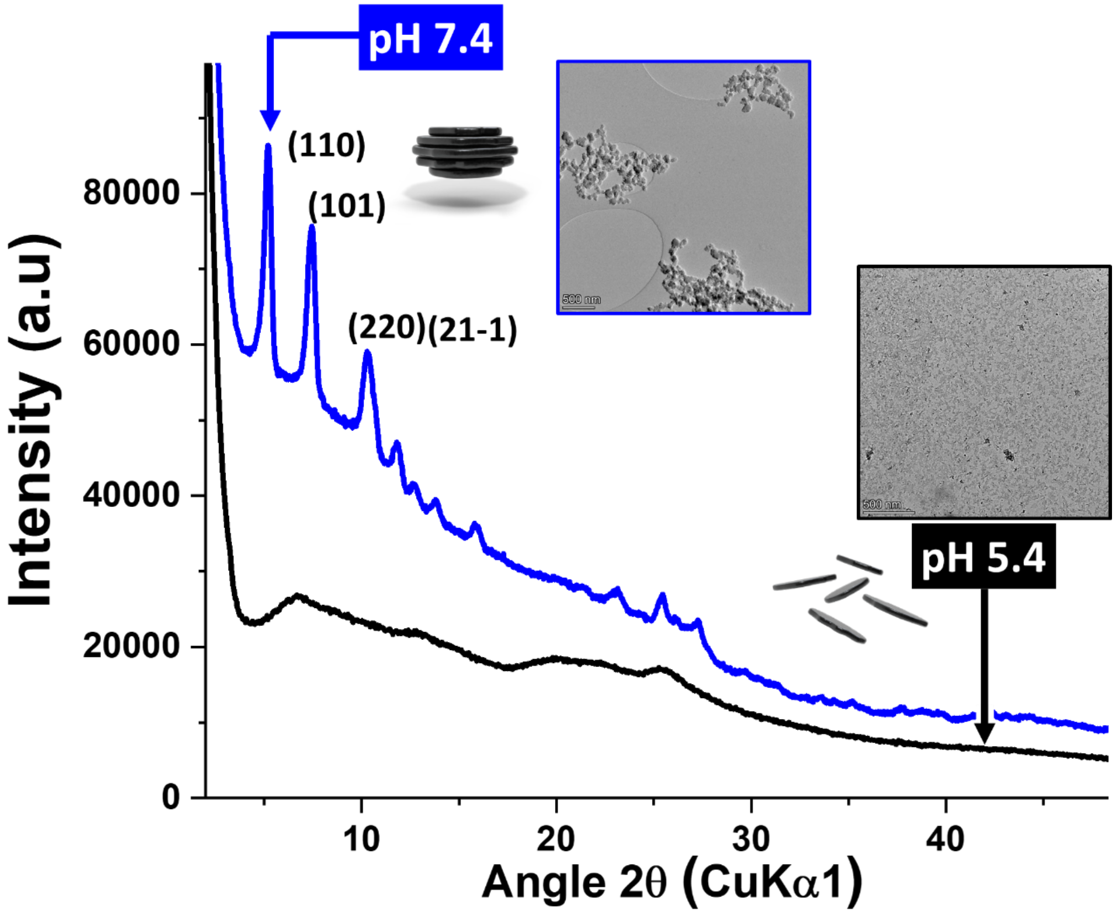

Figure S8. PXRD patterns of Alkyn-nCOF at pH 7.4 (blue) versus 5.4 (black) after 24 hours, with corresponding TEM images.

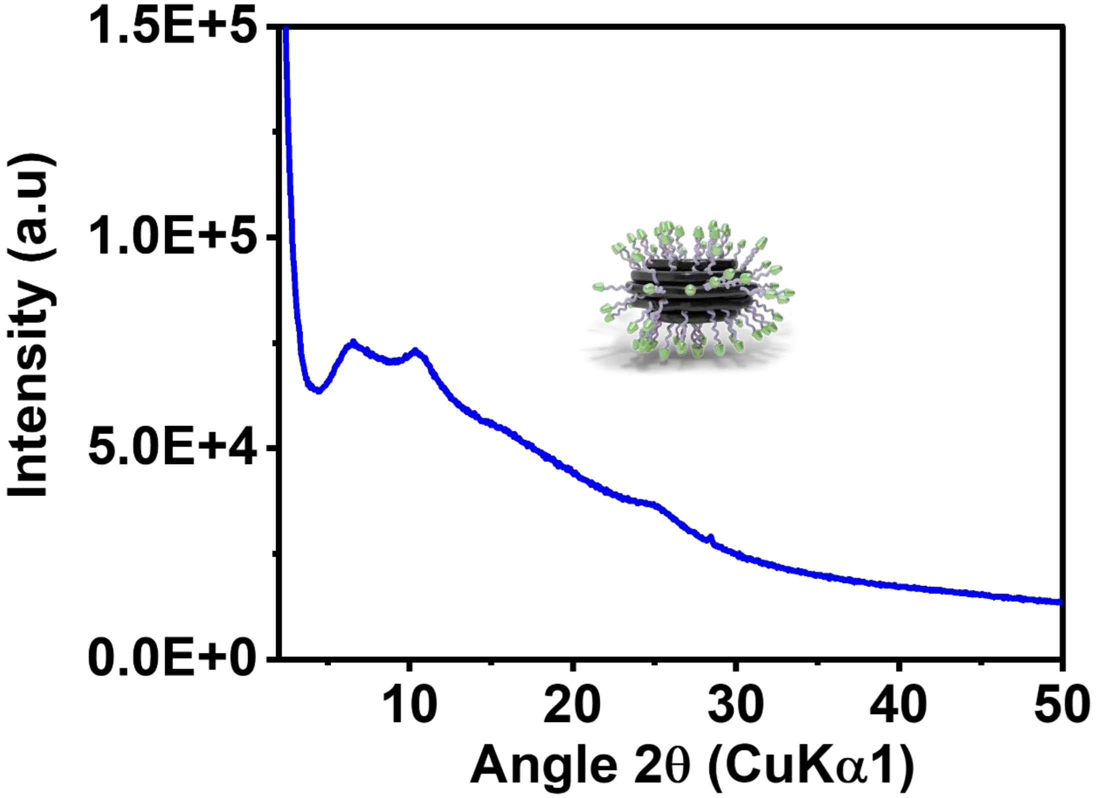

Figure S9. PXRD analysis of Alkyn-nCOF-cRGD.

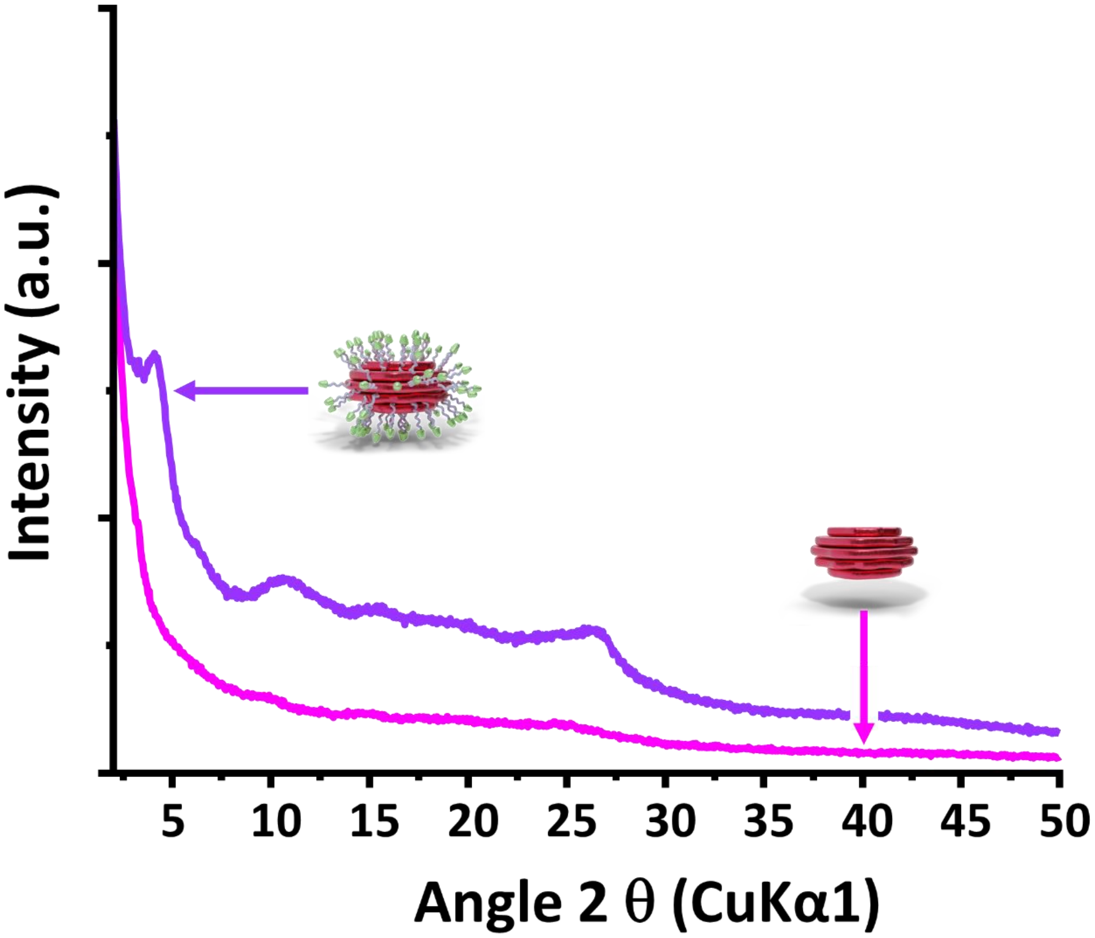

Figure S10. PXRD analysis of Alkyn-nCOF@Dox (pink) and Alkyn-nCOF-cRGD@Dox (purple).

3.5. High-resolution transmission electron microscopy (HRTEM).

High-resolution transmission electron microscopy (HRTEM) images were obtained using Thermo Fisher Scientific (TFS) Talos F200X scanning/transmission electron microscope (S/TEM) with a lattice-fringe resolution of 0.14 nm at an accelerating voltage of 200 kV equipped with a CETA 16M camera. The samples were prepared on holey carbon film mounted on a copper grid. A drop of diluted particle solution was spotted on the grid and dried overnight at room temperature (298 K). The obtained images of periodic structures were analyzed using TIA software. All the relevant areas were marked using bright field imaging mode at spot size 3, and the marked areas were also scanned using the STEM-HDAAF mode at spot size 9 for imaging and spot size 6 for conducting the STEM-EDAX. The STEM mode helps provide the elemental composition as it works on the principle of mass determination. Such measurements can be performed at low electron dose by collecting the high-angle dark-field signal using an annular detector. This mode is generally used to image the elements with different masses, with the heavier mass element appearing brighter. The samples were scanned at spot size 9 and with a screen current of 60 pA. The data was analyzed using Velox analytical software.

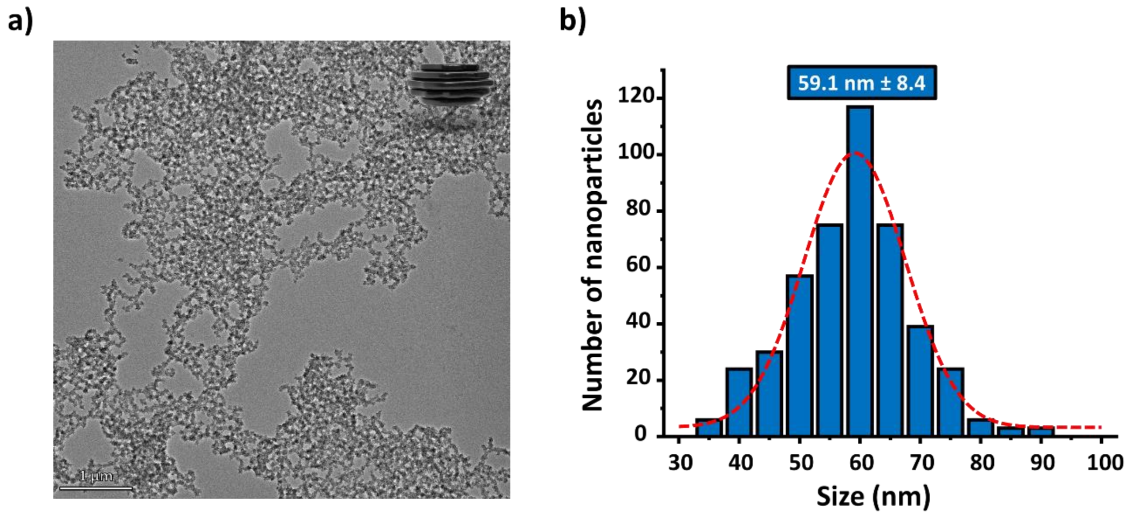

Figure S11. a) Transmission Electron Microscopy (TEM) image of Alkyn-nCOF nanoparticles. b) Particle size distribution of Alkyn-nCOF. The overlaid dashed lines represent Gaussian fit profiles, derived using the mean diameter calculated from the analysis of 500 individual particles.

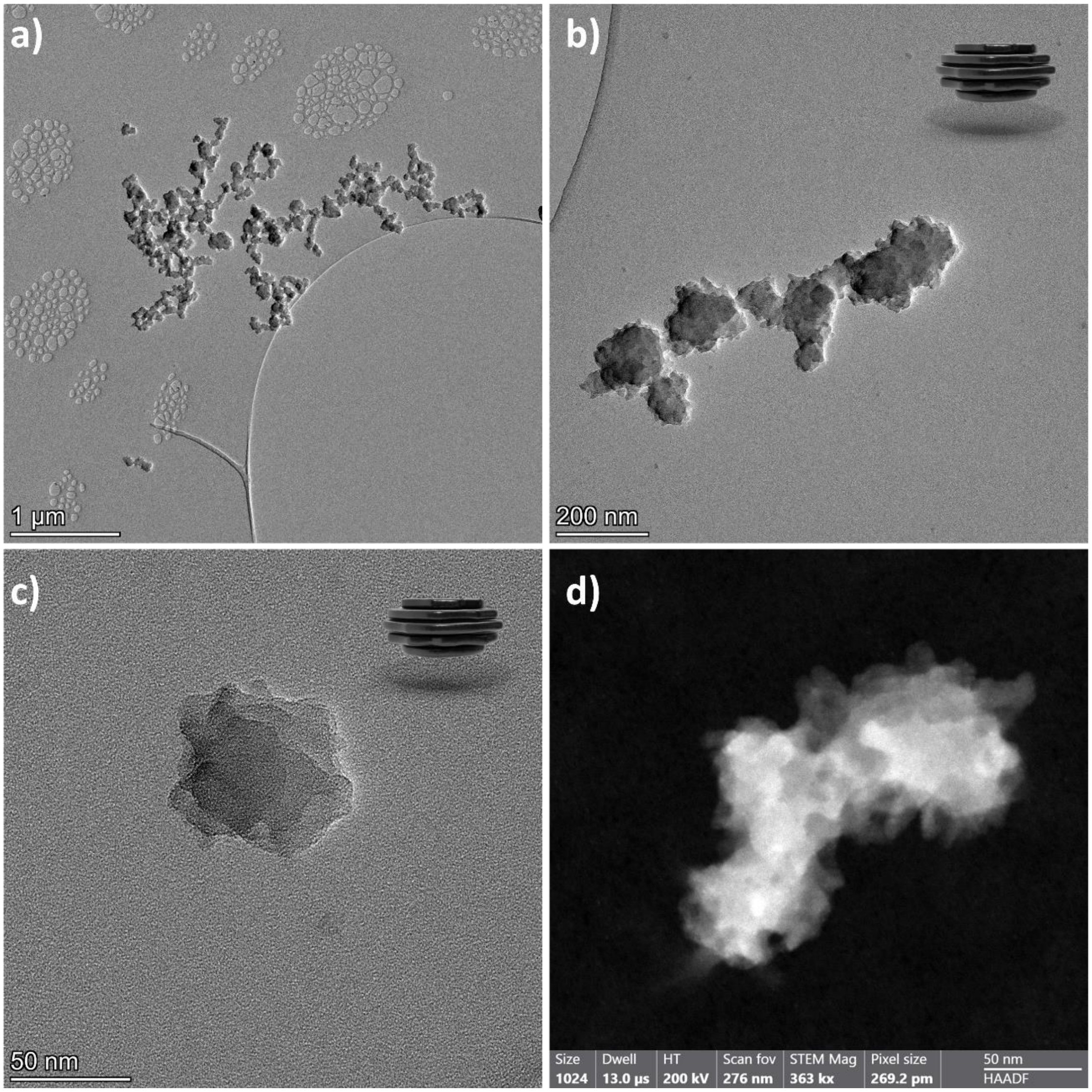

Figure S12. TEM at different magnification (a, b, c) and STEM (d) images of Alkyn-nCOF.

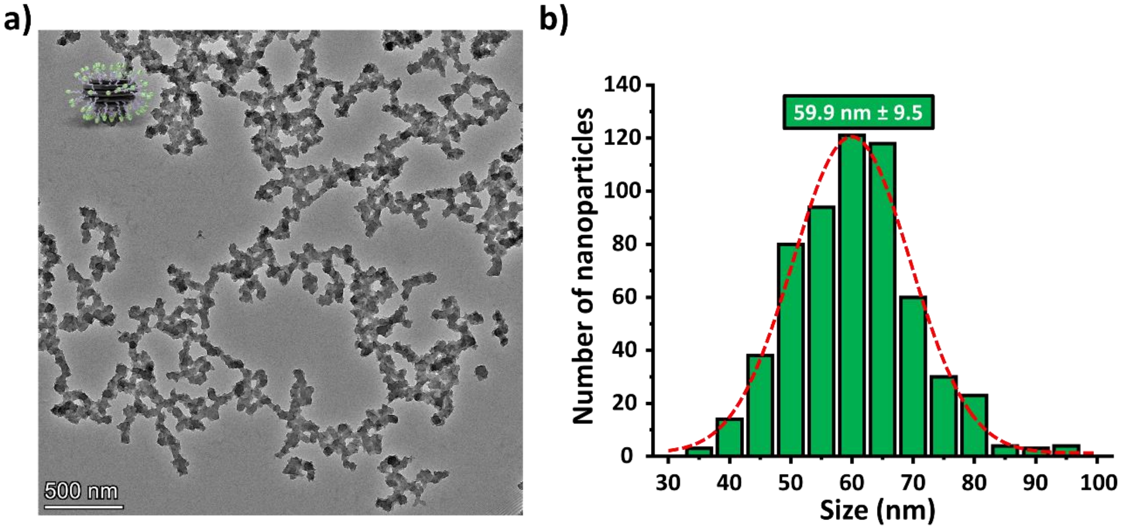

Figure S13. a) Transmission Electron Microscopy (TEM) image of Alkyn-nCOF-cRGD nanoparticles. b) Particle size distribution of Alkyn-nCOF-cRGD. The overlaid dashed lines represent Gaussian fit profiles, derived using the mean diameter calculated from the analysis of 500 individual particles.

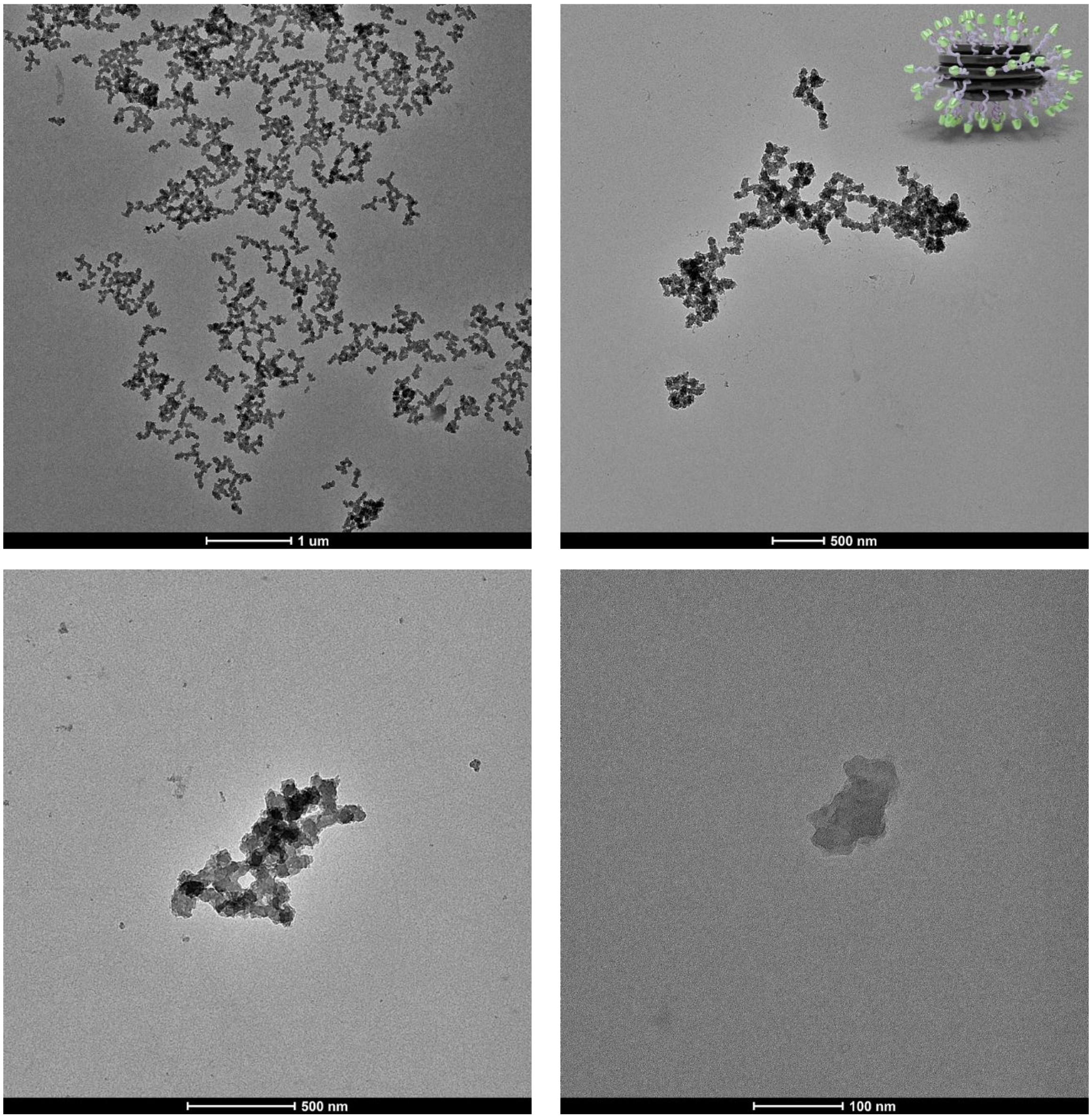

Figure S14. TEM images of Alkyn-nCOF-cRGD at different magnifications.

In-Situ Variable-Temperature Liquid-Cell Transmission Electron Microscopy (VT-LCTEM)

The *in-situ* variable-temperature liquid-cell transmission electron microscopy (VT-LCTEM) experiments were performed using a Protochips Inc. (Morrisville, NC) Poseidon Select liquid-cell TEM holder with on-chip heating capability. A pair of Protochips Poseidon Heating Chips SiNx membrane windows (50 µm × 400 µm and 550 µm × 20 µm lateral window dimensions, respectively) with 50 nm gold spacer were plasma cleaned for 60 s. The chips were loaded on the holder so the windows of the two chips were crossed, so the viewing area was a 50 µm × 20 µm rectangle. This was done to avoid the bulging of the windows. A drop (about 2 µl) of the solution was loaded onto the bottom chip before placing the top chip and sealing the chip setup. The integrity of the windows was visually inspected for any cracks/leaks. The window integrity and O-ring seal was further checked by pumping the holder down to vacuum ($5.4.10^{-6}$ mbar) in a "pre-pump" vacuum chamber. The holder was inserted into the TEM to begin the *in-situ* observations and initiate the on-chip liquid heating.

The two linkers, Alkyn-DFP and TAB, dissolved in dioxane, were drop-casted onto a bottom chip. Once wetting of the liquid cell was confirmed acetic acid was flowed into the cell, and the liquid cell was heated to 110 °C. The formation of sheet-like particles was observed within a few seconds.

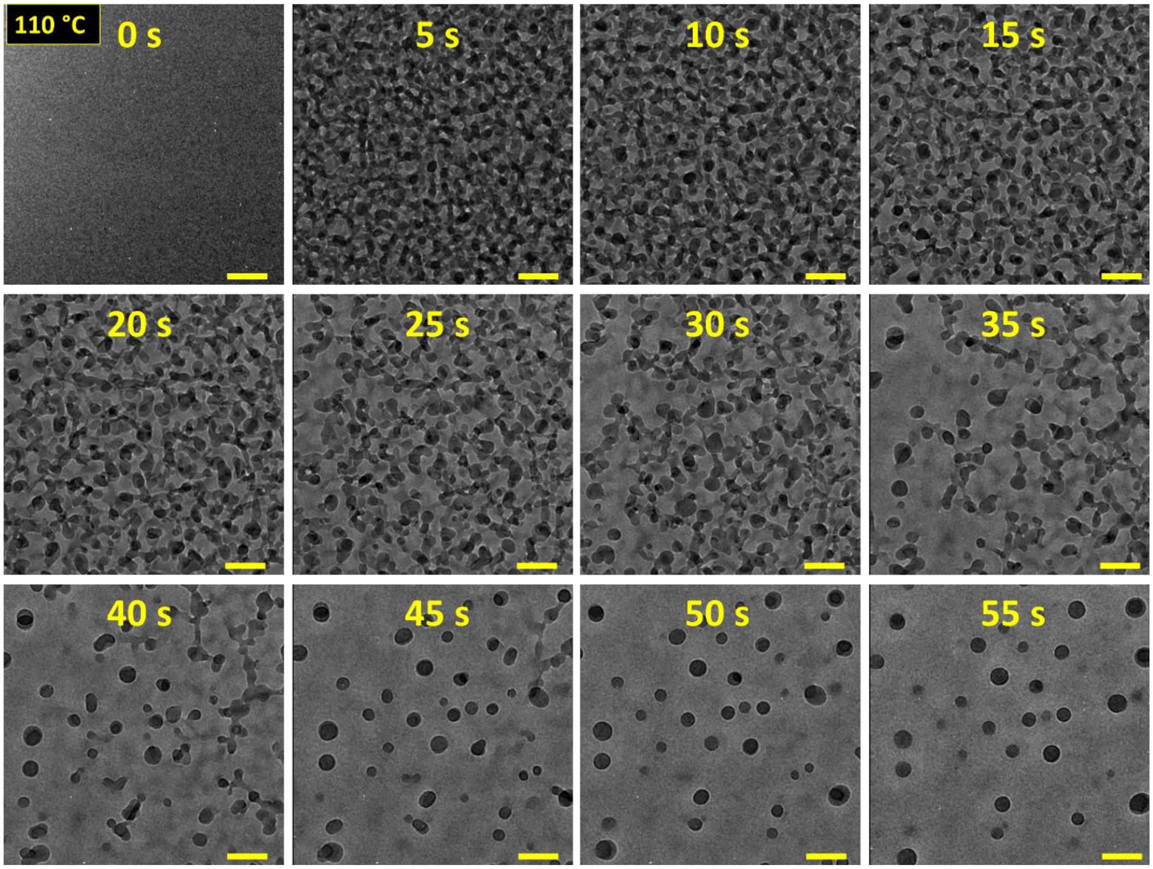

Figure S15. VT-LCTEM experiment: Sequential LCTEM frames extracted from Movie 1 illustrating the *in-situ* heating of TAB and Alkyn-DFP precursor solution to 110 °C in the presence of acetic acid. The initial frame shows the COF precursor solution at 110 °C without nanostructures (t = 0 s), while the following frames capture the changes at different time points in a selected region as the solution is heated. An average particle size of approximately 60 nm is achieved within a minute at 110 °C, maintaining stability over prolonged periods at this temperature. Scale bar = 200 nm.

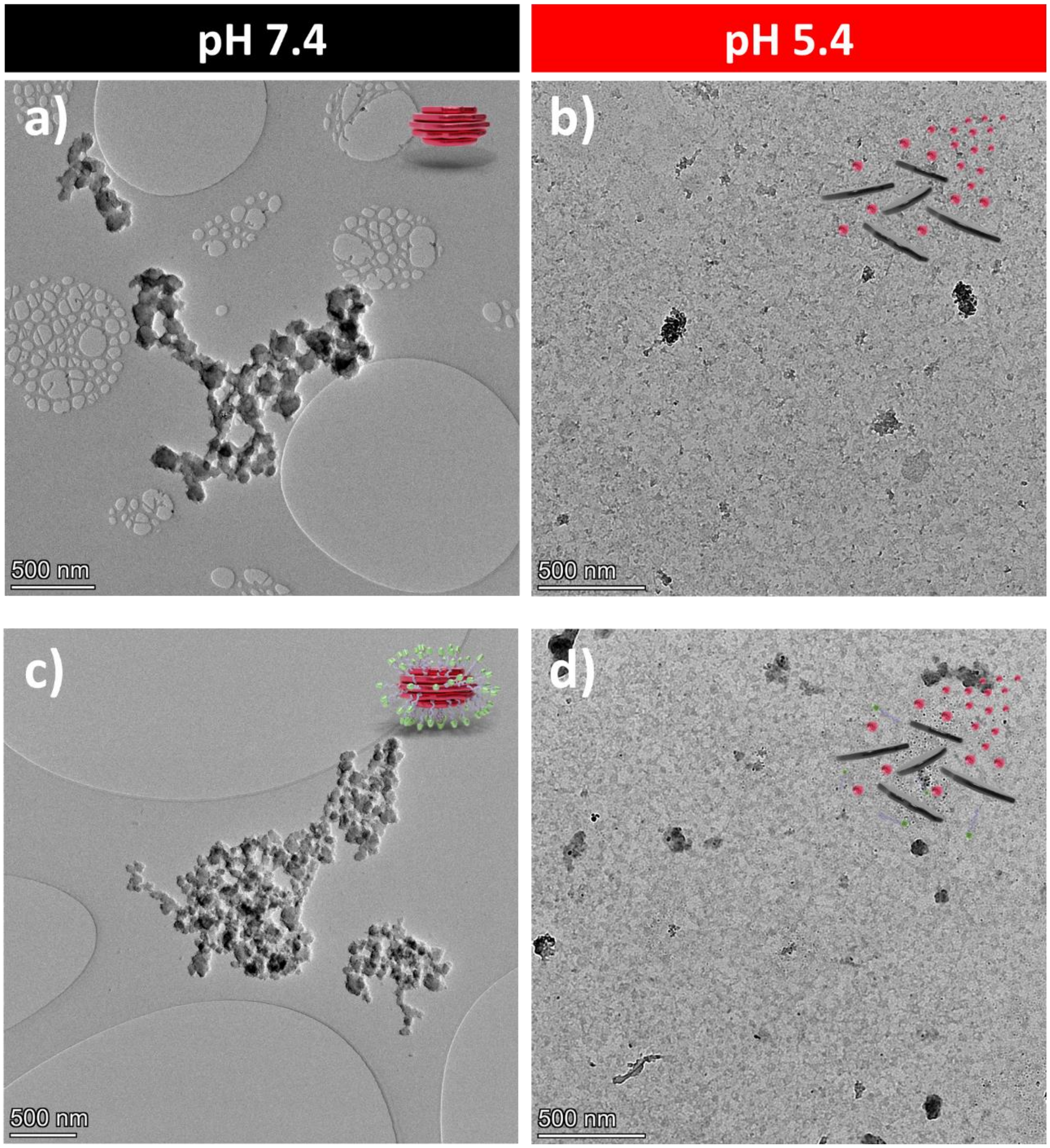

Figure S16. TEM images of Alkyn-nCOF@Dox (a,b) and Alkyn-nCOF-cRGD@Dox (c,d) at pH 7.4 (a,c, black) versus 5.4 (b,d, red) after 24 hours

3.6. Atomic force microscopy (AFM)

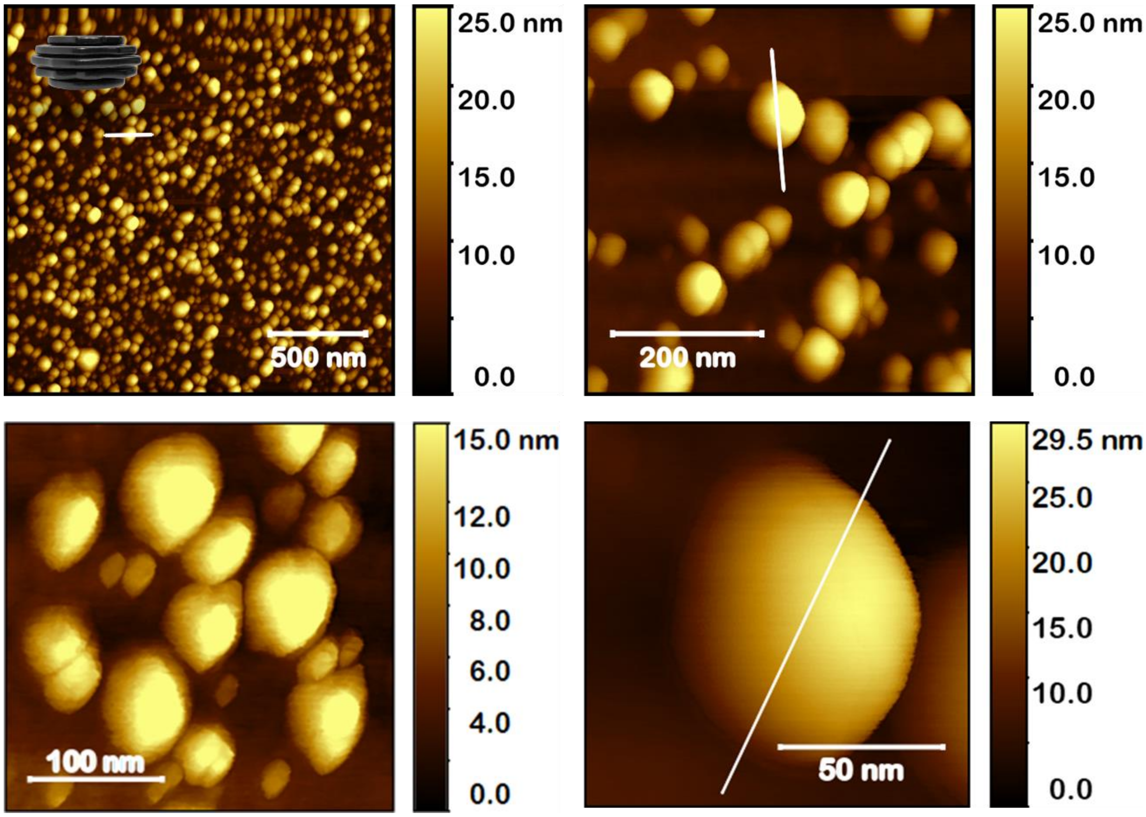

Figure S17. AFM images Alkyn-nCOF at different magnifications.

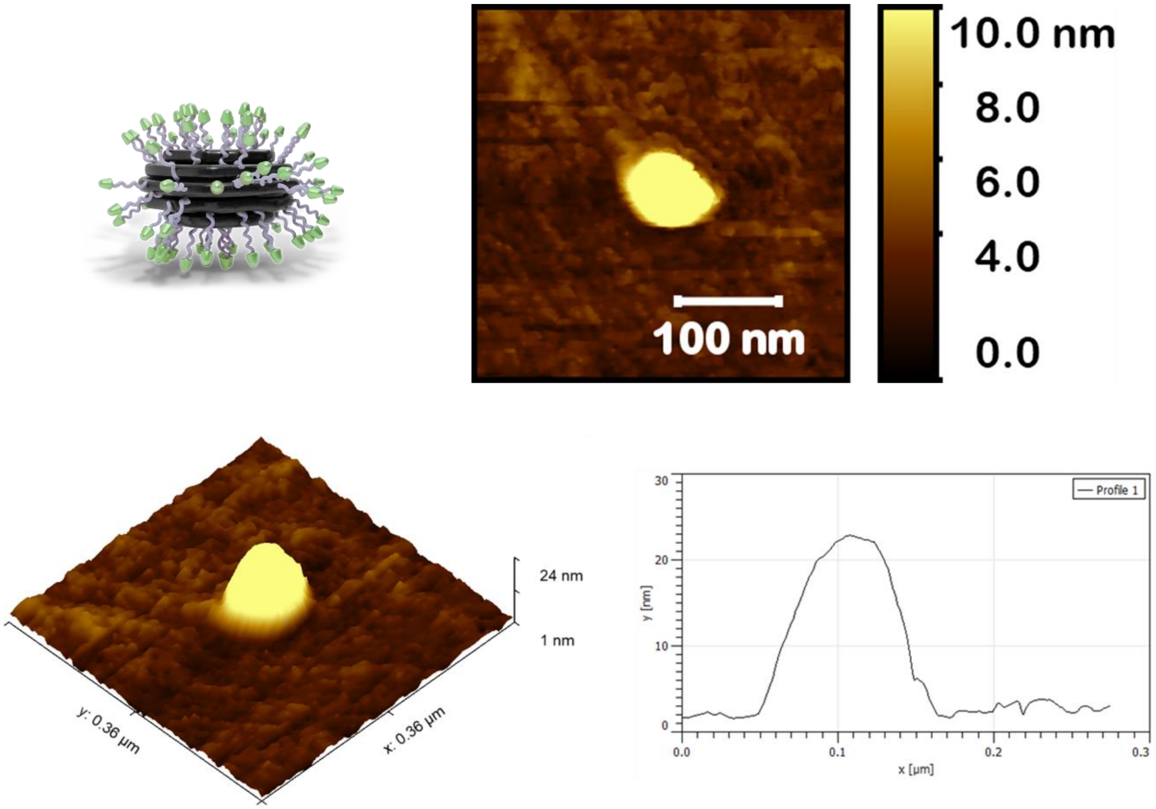

Figure S18. AFM images and height profiles of Alkyn-nCOF-cRGD.

3.7. N_2 adsorption-desorption experiments

Low-pressure gas adsorption measurements were performed on 3-Flex Surface Characterization Analyzer (Micromeritics) at relative pressures up to 1 atm. The cryogenic temperatures were controlled using liquid nitrogen baths at 77 K. The apparent surface area was determined from N_2 adsorption isotherm collected at 77 K by applying the Brunauer-Emmet-Teller (BET) model between P/P_0 values of 0.0005 and 0.05 for microporous COFs.

In a typical experiment, 20 to 30 mg of homogenous polycrystalline samples are transferred (dry) to a 6-mm large bulb glass sample cell and are evacuated at room temperature using a turbo molecular vacuum pump and then gradually heated to 125 °C, held for 24 h and cooled to room temperature.

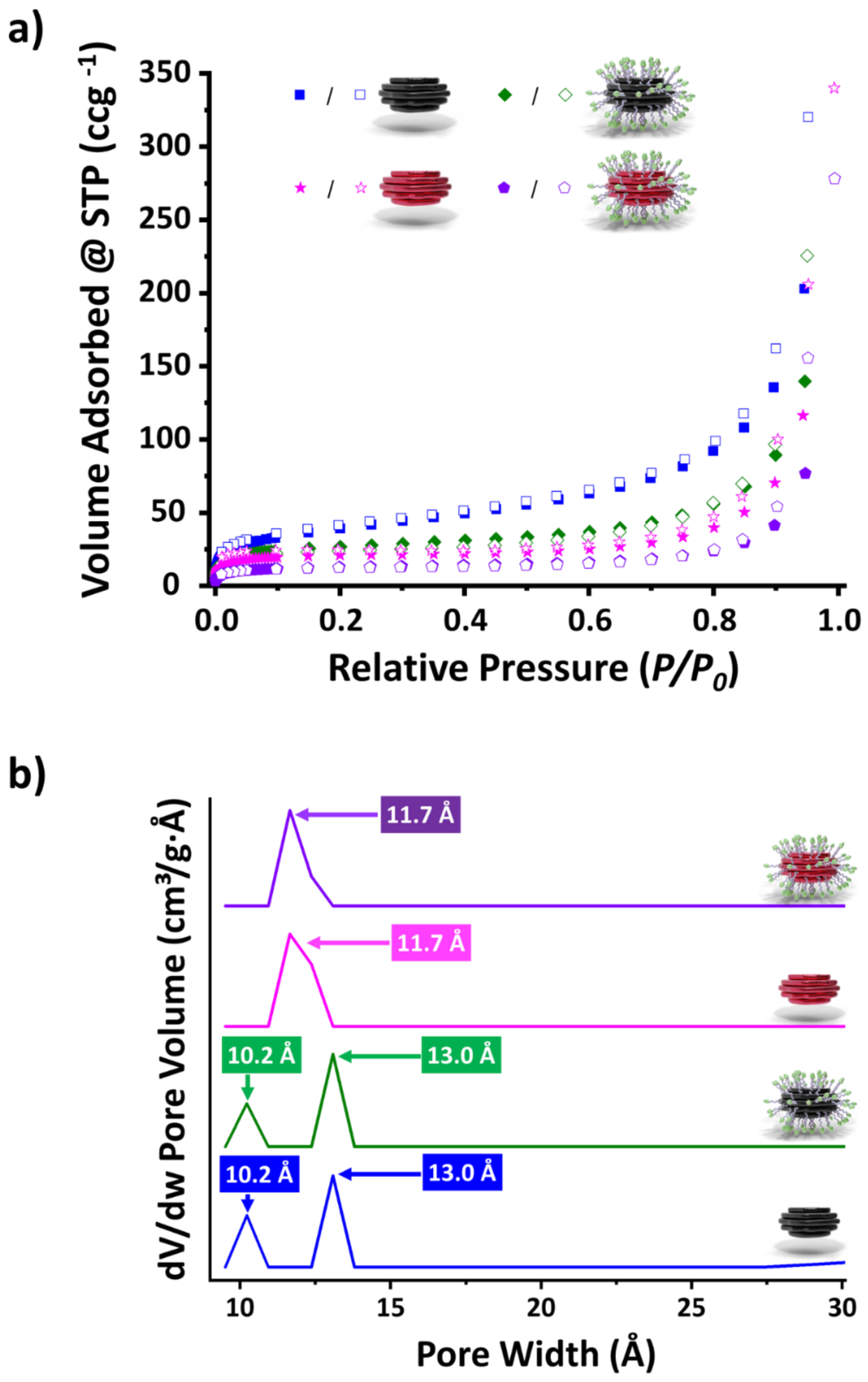

Figure S19. a) Porosity analysis and b) pore size distribution of Alkyn-nCOF (blue), Alkyn-nCOF-cRGD (green), Alkyn-nCOF@Dox (pink) and Alkyn-nCOF-cRGD@Dox (purple). The activation of the sample was carried out at 125 °C for 24 hours under a high vacuum.

3.8. UV-visible spectroscopy

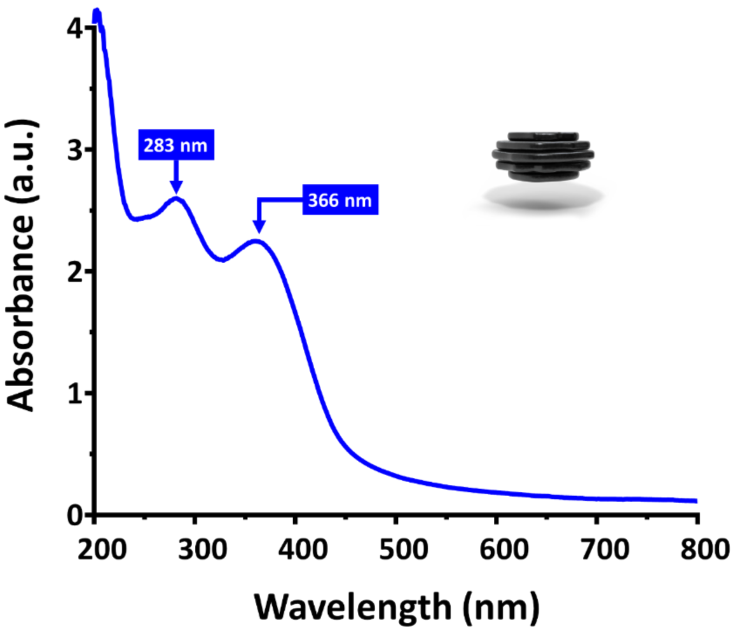

Figure S20. Absorption spectrum of Alkyn-nCOF. The experiment was performed in triplicate.

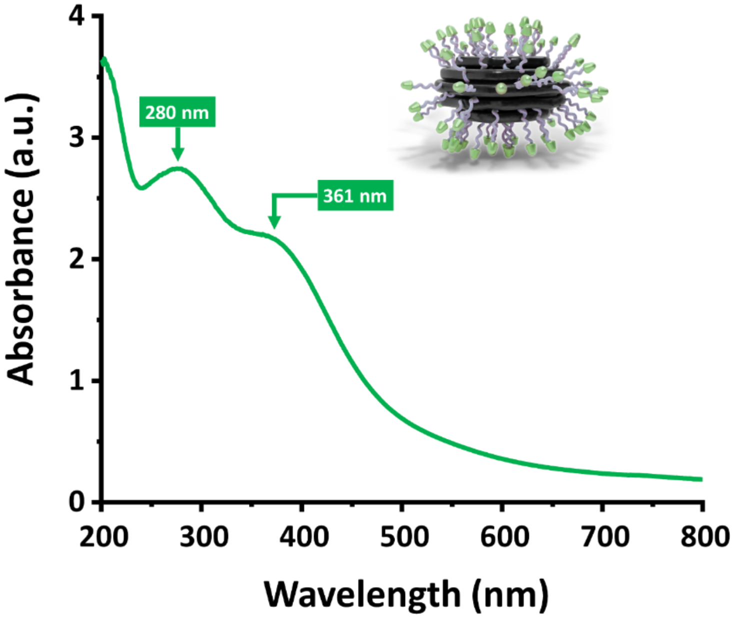

Figure S21. Absorption spectrum of Alkyn-nCOF-cRGD. The experiment was performed in triplicate.

3.9. Fluorescence spectroscopy

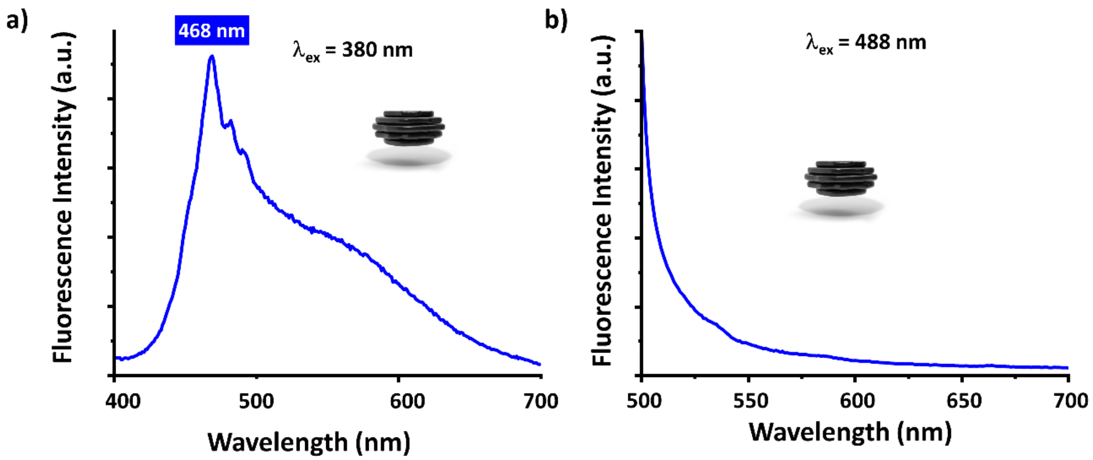

Figure S22. Fluorescence emission spectrum of Alkyn-nCOF at a) λ_{ex}= 380 nm and b) λ_{ex}= 488 nm (H_2O, pH 7.4, 298 K). The experiment was performed in triplicate.

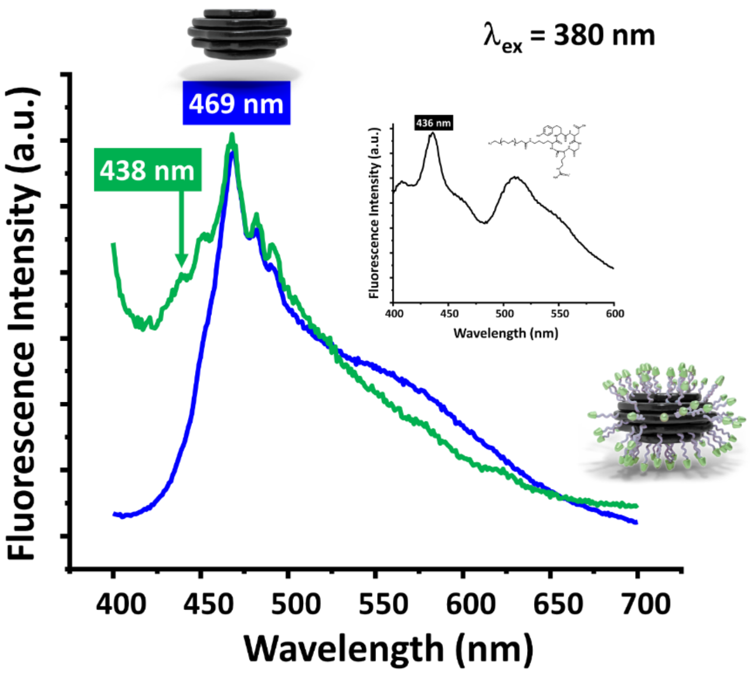

Figure S23. Fluorescence emission spectrum of Alkyn-nCOF (blue) and Alkyn-nCOF-cRGD (green). Inset: Fluorescence emission spectrum of free cRGD-PEG-N_3 (black) (λ_{ex}= 380 nm, H_2O, pH 7.4, 298 K). The experiment was performed in triplicate.

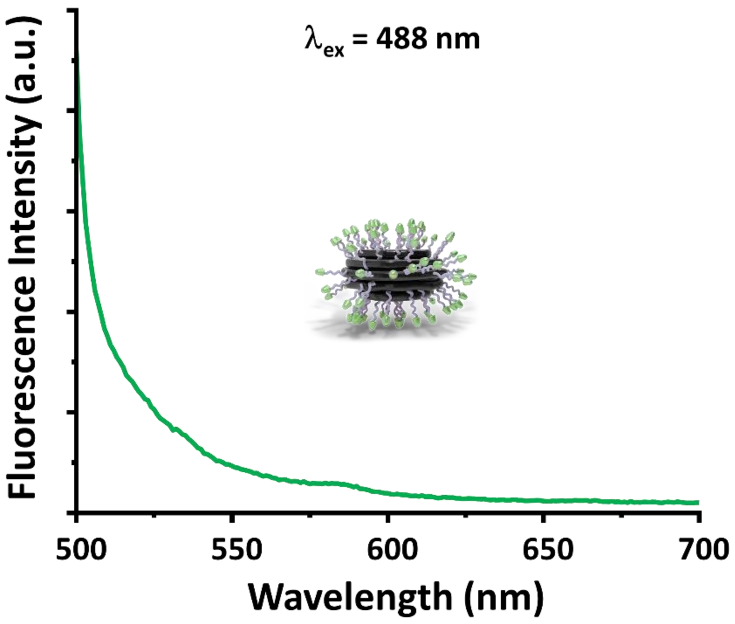

Figure S24. Fluorescence emission spectrum of Alkyn-nCOF-cRGD (green) (λ_{ex}= 488 nm, H_2O, pH 7.4, 298 K). The experiment was performed in triplicate.

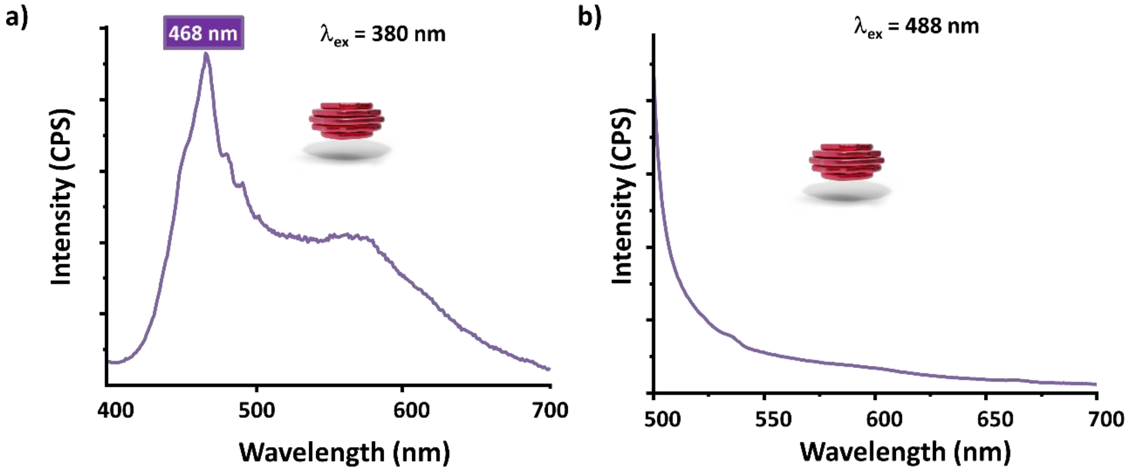

Figure S25. Fluorescence emission spectrum of Alkyn-nCOF@Dox at a) λ_{ex}= 380 nm and b) λ_{ex}= 488 nm (H_2O, pH 7.4, 298 K). The experiment was performed in triplicate.

3.10. Thermogravimetric analysis (TGA)

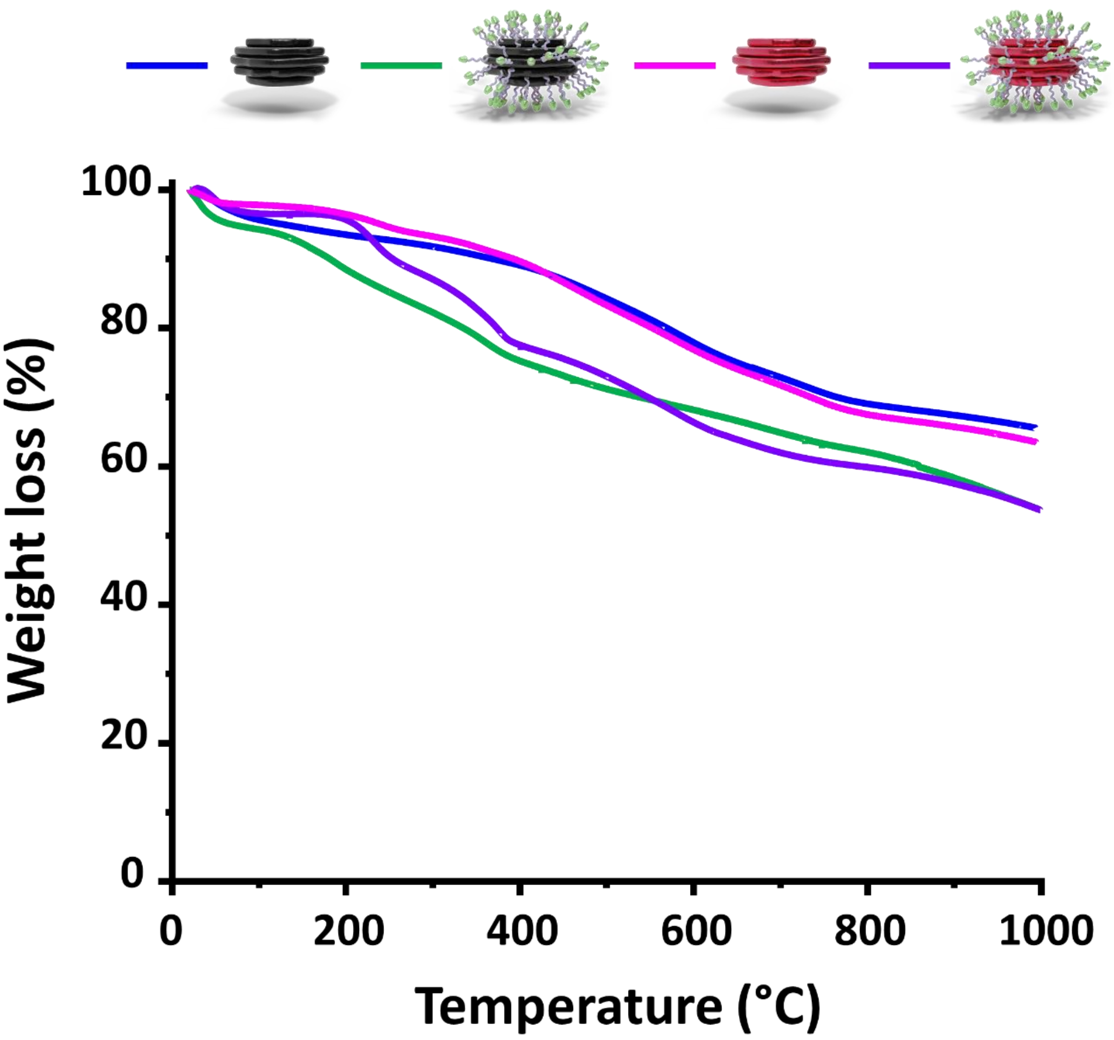

Figure S26. Thermogravimetric analysis (TGA) of Alkyn-nCOF (blue), Alkyn-nCOF-cRGD (green), Alkyn-nCOF@Dox (pink) and Alkyn-nCOF-cRGD@Dox (purple). The thermal stability of the COF profile was recorded at a scan rate of 5 °C/min.

3.11 Dynamic light scattering (DLS)

DLS measurements were carried out on a Zetasizer Nano-ZS (Malvern Instruments) to determine the Zeta(ζ)-potential as well as the hydrodynamic size of the particles. All samples were analyzed at room temperature in 10 mM phosphate-buffered saline (PBS). Each experiment was performed in triplicate.

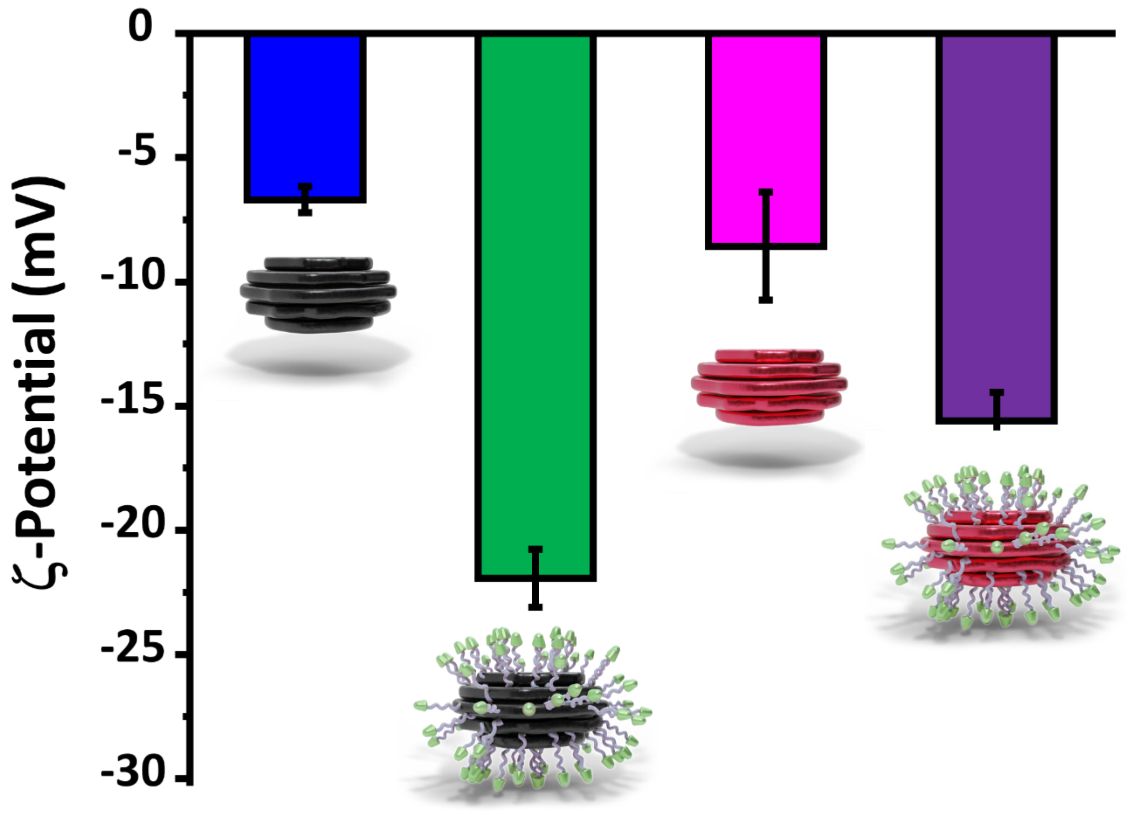

Figure S27. Zeta(ζ)-potential of Alkyn-nCOF (blue), Alkyn-nCOF-cRGD (green), Alkyn-nCOF@Dox (pink), and Alkyn-nCOF-cRGD@Dox (purple) at pH 7.4. The experiment was performed in triplicate.

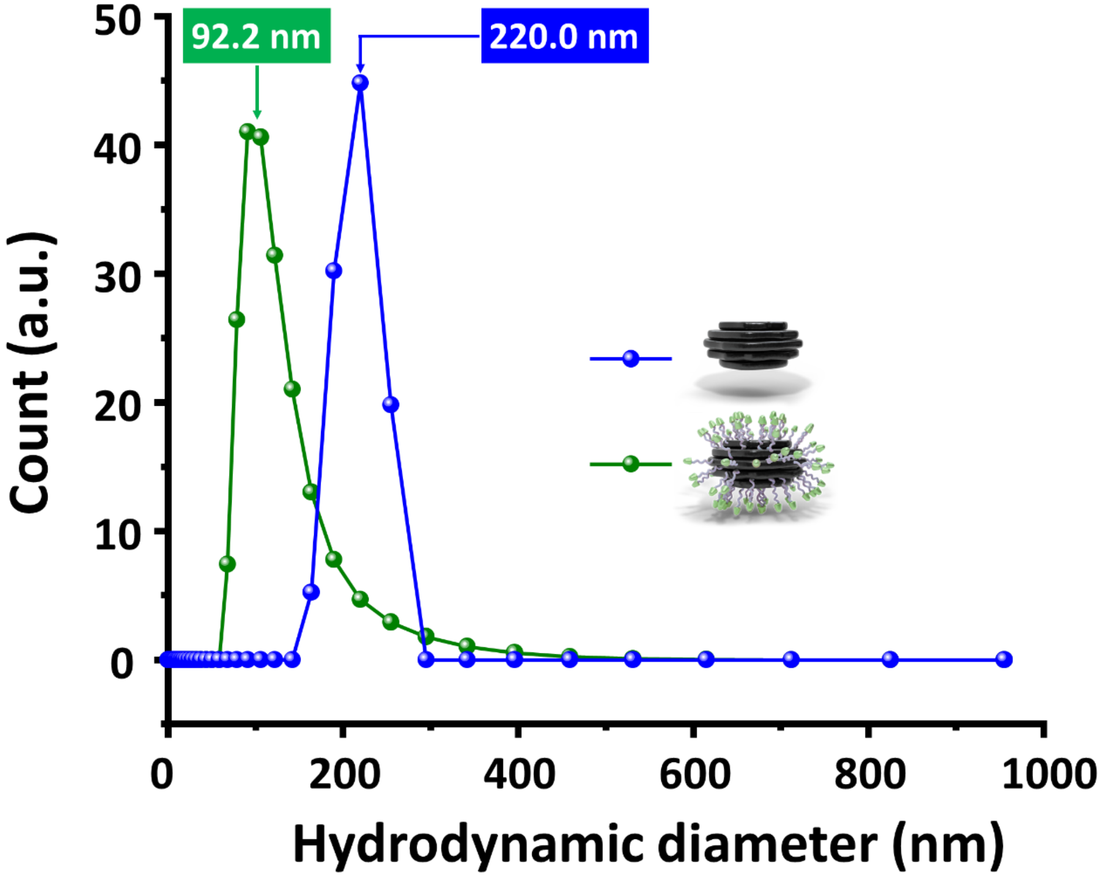

Figure S28. Hydrodynamic diameter of Alkyn-nCOF (blue) and Alkyn-nCOF-cRGD (green) at pH 7.4. The experiment was performed in triplicate.

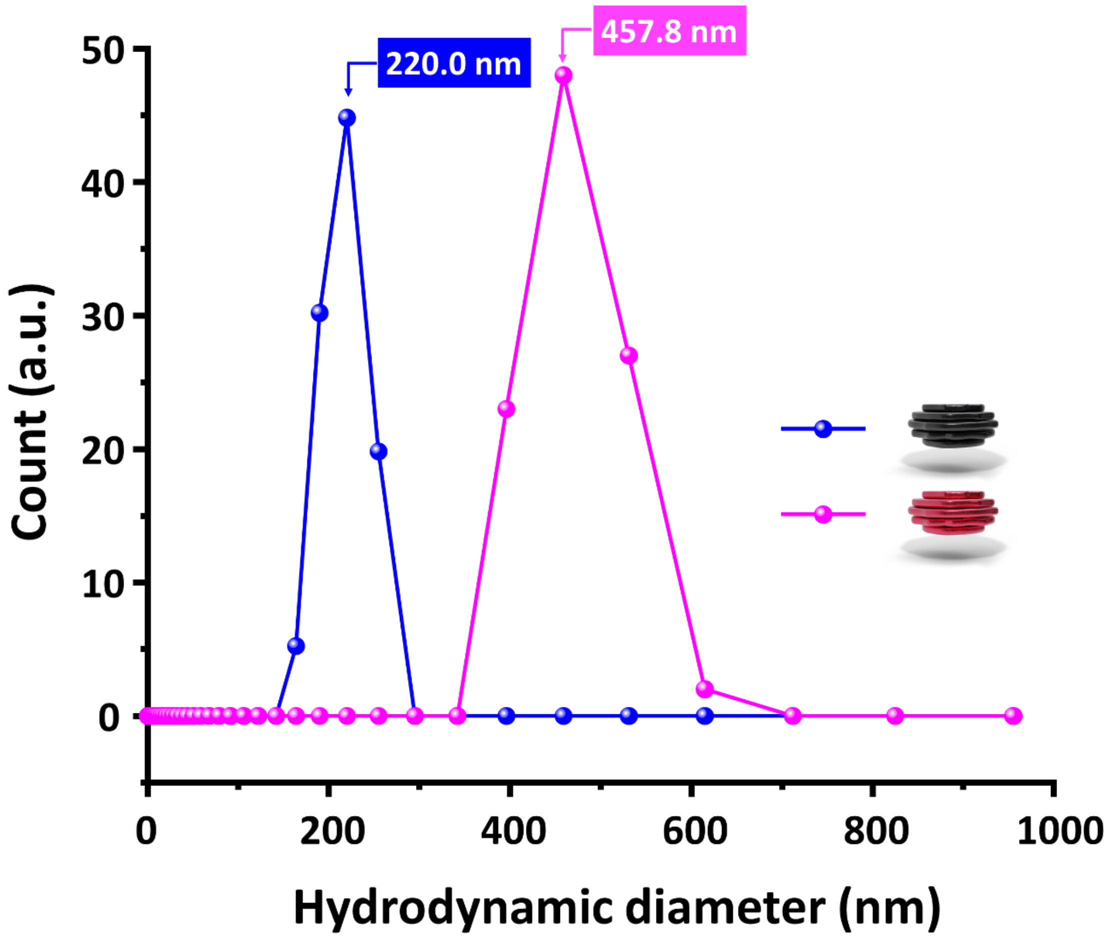

Figure S29. Hydrodynamic diameter of Alkyn-nCOF (blue) and Alkyn-nCOF@Dox (pink) at pH 7.4. The experiment was performed in triplicate.

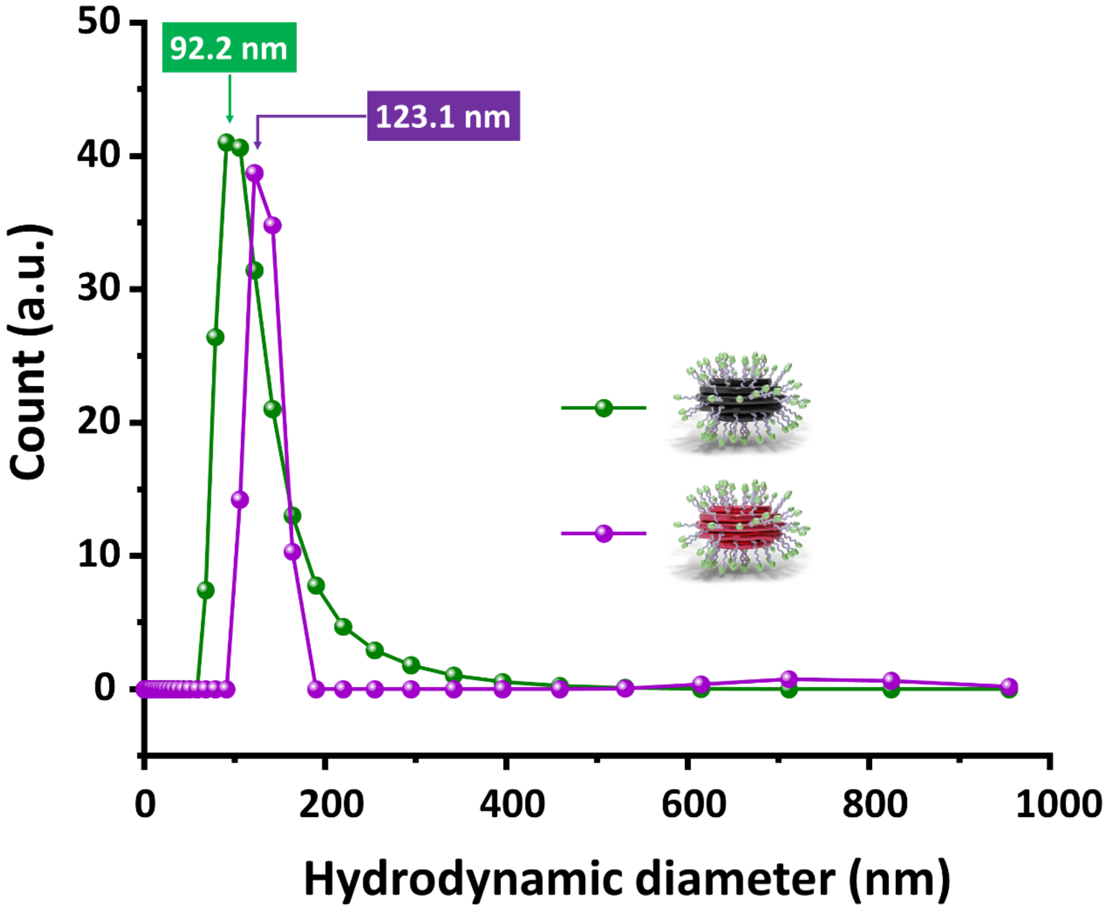

Figure S30. Hydrodynamic diameter of Alkyn-nCOF-cRGD (green) and Alkyn-nCOF-cRGD@Dox (purple) at pH 7.4. The experiment was performed in triplicate.

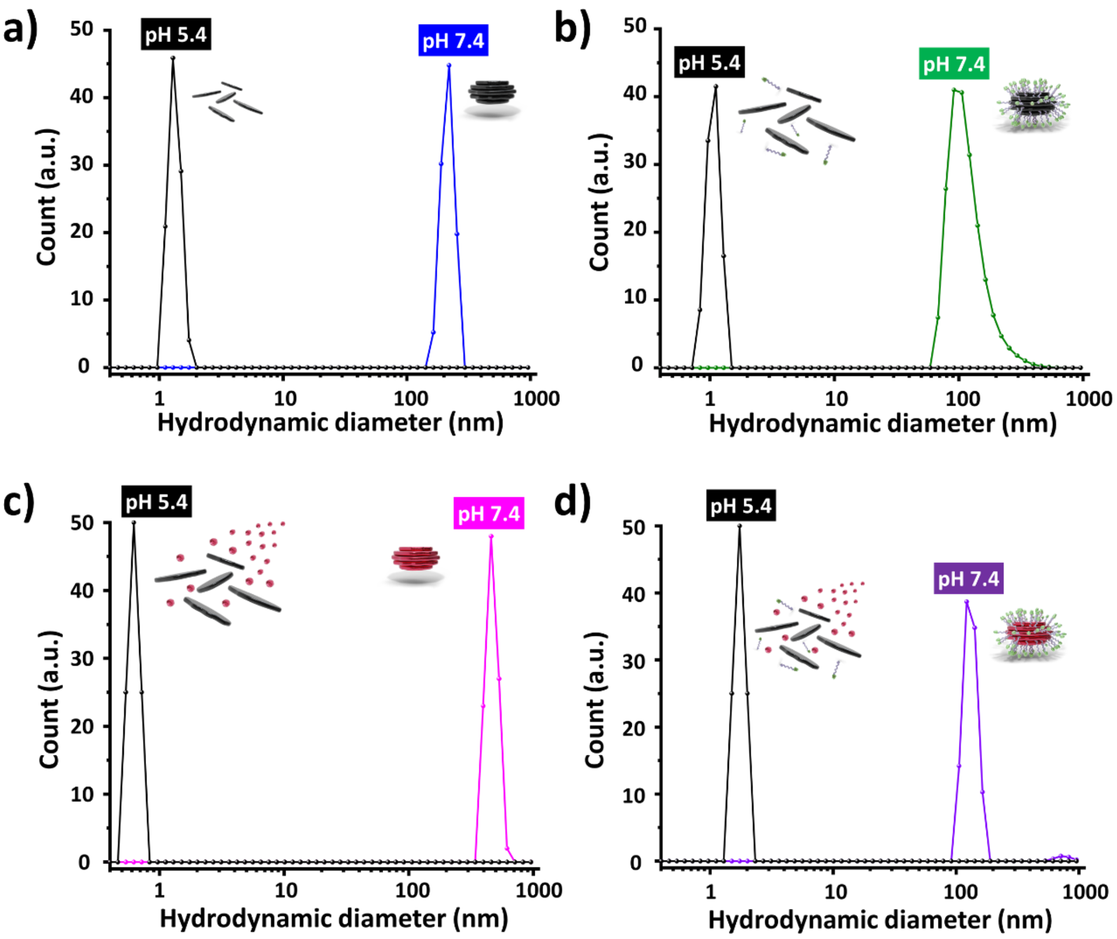

Figure S31. Hydrodynamic diameter of a) Alkyn-nCOF (blue), b) Alkyn-nCOF-cRGD (green), c) Alkyn-nCOF@Dox (pink), and d) Alkyn-nCOF-cRGD@Dox (purple) at pH 7.4 and pH 5.4 (black). The experiment was performed in triplicate.

Investigation of the biostability of Alkyn-nCOF-cRGD@Dox within various environments:

- **pH 4.0 and pH 5.4:** Both conditions resulted in a complete loss of nanoparticle integrity. The hydrodynamic size and TEM images of the nanoparticles decreased below 10 nm, indicating significant degradation in highly acidic environments (Figure S32).
- **pH 6.4:** The nanoparticles retained a size of approximately 60 nm, suggesting a significant but controlled release of Dox while maintaining some structural integrity in mildly acidic conditions (Figure S32).

• **pH 7.4 (Physiological conditions):** At pH 7.4, under physiological conditions such as in normal phosphate-buffered saline (PBS), cell culture medium, and fetal bovine serum (FBS), there were no significant morphological changes observed in Alkyn-nCOF-cRGD@Dox within 24 hours, as demonstrated in Figures S32 and S33. Although DLS measurements showed a slight increase in size compared to TEM data due to minor particle aggregation, the sizes consistently remained below 300 nm. This size range is optimal for ensuring effective drug release, indicating that the nanoparticles maintain their functional integrity and are suitable for drug delivery applications under physiological conditions.

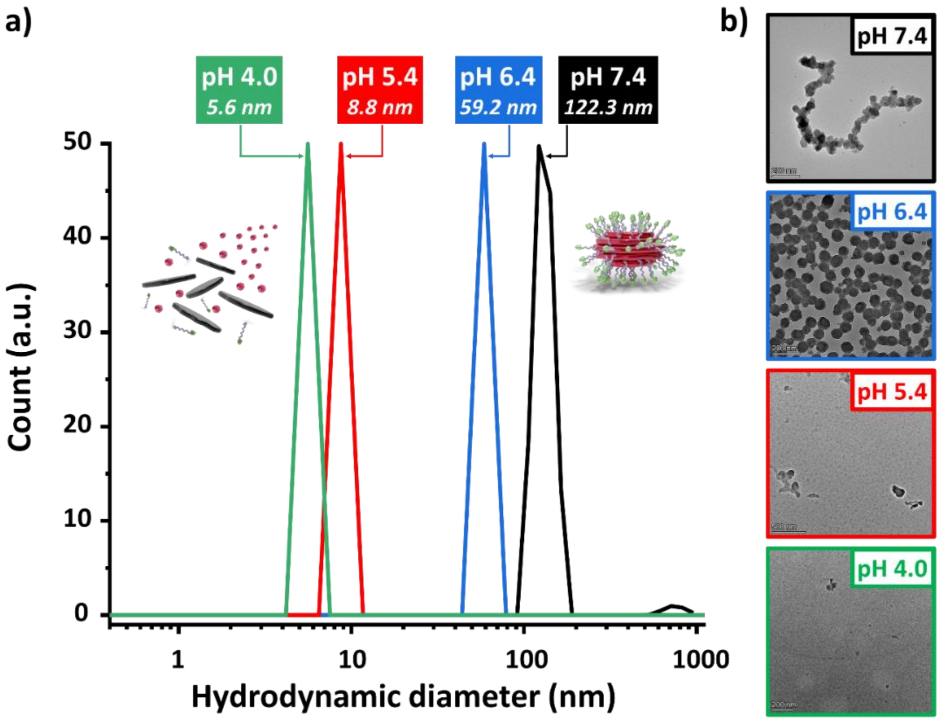

Figure S32. Dynamic Light Scattering (DLS) and Transmission Electron Microscopy (TEM) analysis of Alkyn-nCOF-cRGD@Dox under various pH conditions. This figure shows the response of Alkyn-nCOF-cRGD@Dox nanoparticles to different pH environments (pH 4.0 (green), pH 5.4 (red), pH 6.4 (blue), and pH 7.4 (black)). The DLS data show the change in hydrodynamic size from pH 4.0 to pH 7.4, demonstrating significant degradation at highly acidic conditions (pH 4.0 and 5.4) and stability at physiological pH (6.4 and 7.4). Corresponding TEM images (insets) confirm

the morphological integrity under these conditions, highlighting the controlled release mechanism and stability that are essential for targeted cancer therapy.

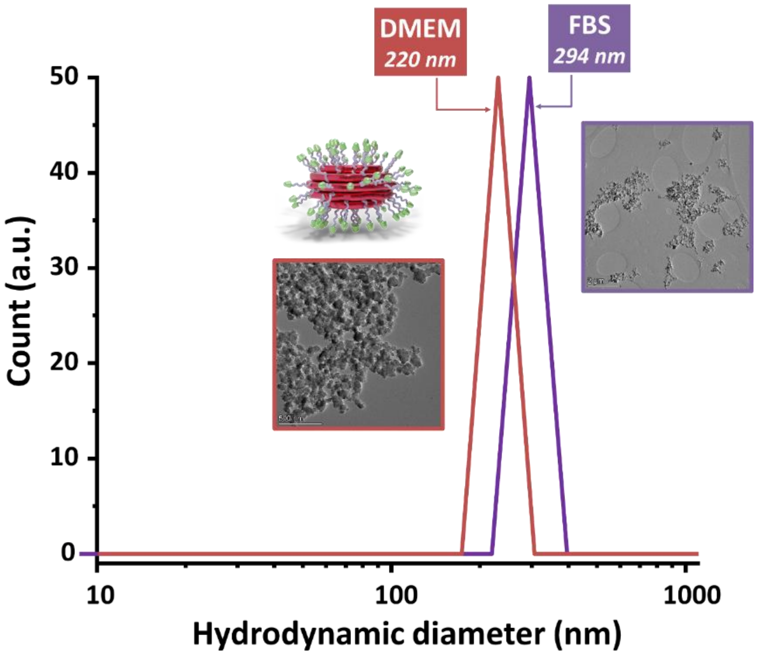

Figure S33. Stability of Alkyn-nCOF-cRGD@Dox in different biological media. This figure shows the DLS and TEM analysis of Alkyn-nCOF-cRGD@Dox nanoparticles in various biological media, including cell culture medium (DMEM, red) and fetal bovine serum (FBS, purple). The results demonstrate stable particle sizes and unchanged morphologies in both media over 24 hours, as shown in the TEM images (insets). These results confirm the biostability of the nanoparticles under biological conditions relevant to their therapeutic application, while maintaining their structural integrity, which is essential for effective drug delivery.

3.12. Quantification of cRGD-PEG-N_3 conjugated using the OPA method

The O-phthalaldehyde (OPA) method formed a fluorescent product after a reaction with a primary amine. OPA reacts to the primary amines and lysine side-chains of proteins in the presence of a thiol, usually 2-mercaptoethanol, under alkaline conditions and gives a fluorescence signal at 436–475 nm after excitation at 330–390 nm. The fluorescence signal is proportional to the amount of protein in the sample, allowing a sample to be quantified by

comparing it to a standard curve of known protein content.[10] 100 µL of the sample was added to 1 mL of OPA reagent, and fluorescence measurement was recorded precisely 2 minutes after addition.

To avoid contamination or overlap between species' signals, the amount of peptide conjugated was calculated using the supernatant containing the peptide not conjugated in the NP surface at t=0 and t=24 hours.

The efficiency of peptide conjugation on nanoparticle surfaces is expressed in weight % and represents the percentage of the total nanoparticle weight that corresponds to the weight of the conjugated peptides. It indicates how much of the nanoparticles' surface is covered with the desired peptides.

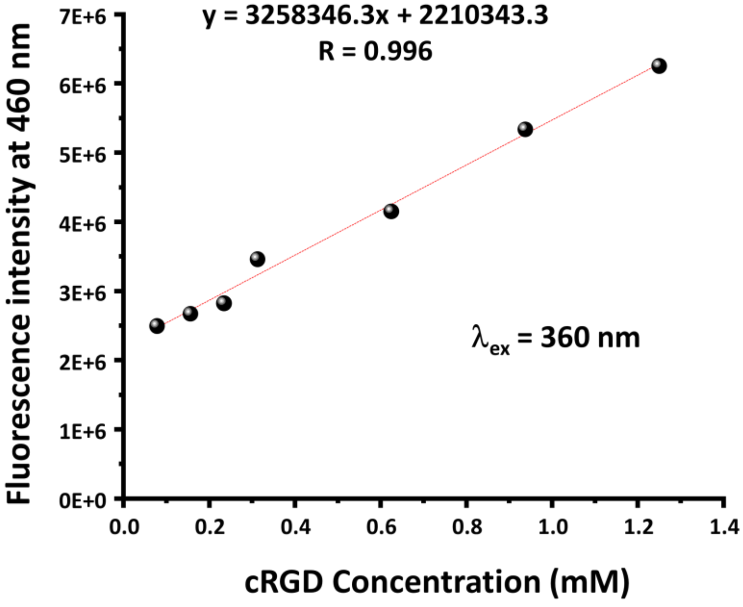

Figure S34. Calibration curve obtained by measuring the maximum fluorescence signal by OPA method at different cRGD-PEG-N_3 concentrations (λ_{max} = 460 nm, H_2O, pH 7.4, 298 K).

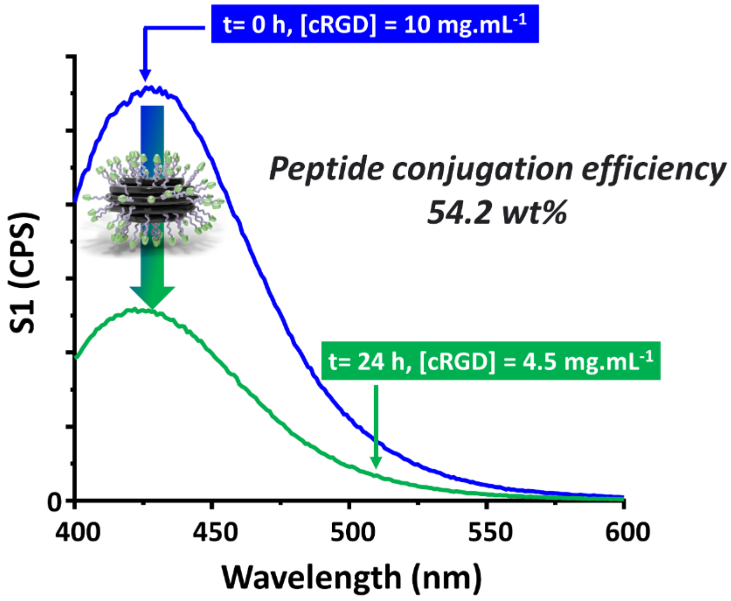

Figure S35. Fluorescence emission spectra of diluted supernatant solution before (blue curve) and 24 h after addition of Alkyn-nCOF (green curve) to determine the protein content covalently conjugated. Fluorescence intensity was used to deduce the conjugation efficiency (λ_{ex}= 360 nm, H_2O, pH 7.4, 298 K). The experiment was performed in triplicate.

3.13. Quantification of Dox loaded using fluorescence spectroscopy

To avoid contamination, the overlap between species' signals, and possible quenching of the Dox fluorescence due to its pore confinement, the Dox loading was calculated using the supernatant containing the Dox that was not loaded in the porous structure by fluorescence spectroscopy based on comparison to a calibration curve of the Dox standard solution (λ_{ex} = 488 nm).

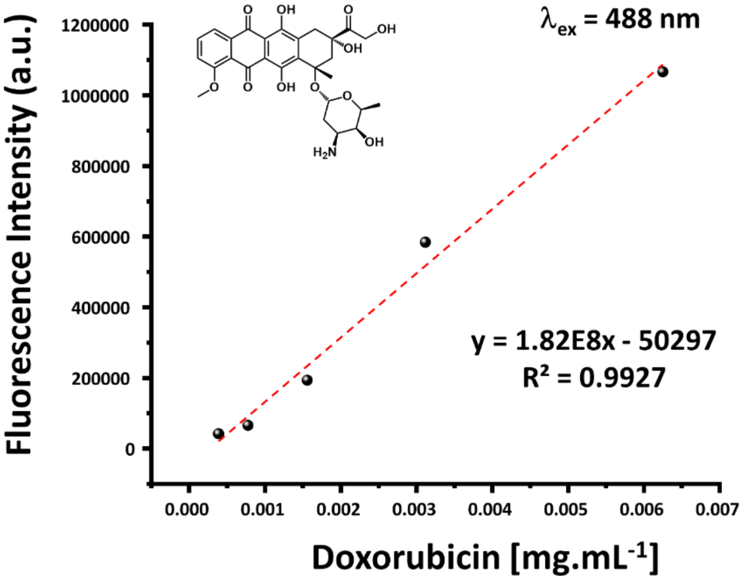

Figure S36. Calibration curve obtained by measuring the maximum fluorescence signal at different Dox concentrations (λ_{max} = 560 nm, H_2O, pH 7.4, 298 K).

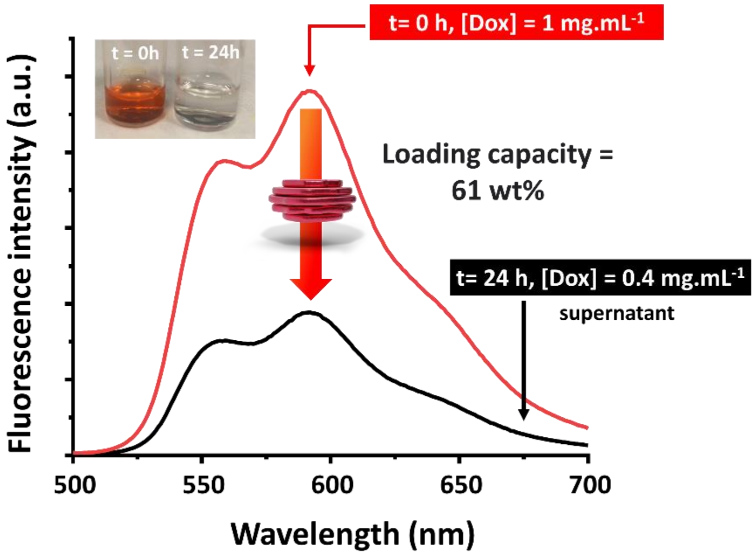

Figure S37. Fluorescence emission spectra of diluted supernatant solution before (red curve) and after Alkyn-nCOF (black curve) was added. Fluorescence intensity was used to deduce the loading efficiency (λ_{ex}= 488 nm, H_2O, pH 7.4, 298 K). Inset picture displays supernatant before (left) and after the addition of the NPs. The experiment was performed in triplicate.

Table S4. Reported COFs for Dox Delivery.

COF	Size (nm)	Loading Efficiency	Biological assessment	Ref
**TAPB-DMTP-COF**	100-150	32.1 wt%	*In vivo:* nude mice with H22 hepatoma cells xenograft tumors	Liu et *al.*[11]
**PEG2000-CCM/APTES-COF**	150-300	9.71 ± 0.13 wt%	*In vivo:* nude mice with HeLa cell xenograft tumors	Zhang et *al.*[12]
**COF-DhaTab**	100-500	35 wt%	-	Kandambeth et *al.*[13]
**COF**	200	35 wt%	*In vitro:* A549 cell line	Wang et *al.*[14]
**F68/SS-COF**	150	21 wt%	*In vitro:* HepG2 cells	Liu et *al.*[15]
**TAB-DFP-nCOF**	240	14.3 ± 0.15 wt%	*In vitro:* U251-MG glioblastoma cells<br>*In vivo*: zebrafish embryos	Benyettou et *al.*[16]
**HY/SS-CONs**	120 ± 20	18 wt%	*In vitro:* HepG2 cells	Wang et *al.*[17]
**F68@SS-COFs**	140 ± 15 nm	21%	*In vitro:* HepG2 cells	Shuai et *al.*[18]
**COF@IR783**	200 nm	low loading	*In vivo:* nude mice with 4T1 cells xenograft tumors	Wang et *al.*[19]
**DOX@COF(Fe)**	100 nm	1.6 mg mg^{-1}	*In vivo:* MCF-7/Adr tumor-bearing nude mice	Gao et *al.*[20]
**DOX@COF-PEG-T10.**	171.22 ± 1.8 nm	17.84 ±2.3%	*In vivo:* Glioma-bearing mice	Huo et *al.*[21]
**COF HP**	100 nm	29.1%	*In vitro:* L929 cells	Zhou et *al.*[22]
**TpDh NPs**	100 nm	0.330 mg/mg	*In vitro:* MCF-7 and MCF-10A cells	Gao et *al.*[23]
**TCOF-PEG**	50–100 nm	8%	*In vitro:* HeLa cell line	Anbazhagan et *al.*[24]
**hCOF nanocage**	200 nm	10.2%	*In vivo:* 4T1 breast cancer model in mice	Yao et *al.*[25]
**COF-LZU1**	186 nm	44.1%	-	Zou et *al.*[26]
**CuS@COFs-BSA-FA/DOX**	300 nm	4.35 %	*In vivo:* 4T1 breast cancer model in mice	Wang et *al.*[27]
**Alkyn-nCOF and Alkyn-nCOF-cRGD**	60 nm	61 and 42 wt%	*In vivo:* MDA-MB-231 tumor-bearing nude mice	This work

3.14. pH-dependent Dox release experiments

The effect of pH on the release of Dox from Alkyn-nCOF and Alkyn-nCOF-cRGD was monitored over time in water buffered with PBS (10 mM) at 37 °C and pH = 7.4 and 5.4. The pH of the solutions was adjusted using a 1 M HCl(aq) solution. At regular intervals, solutions were centrifuged, and supernatants were collected. The solution fluorescence intensity was measured compared to a calibration curve (Figure S34).

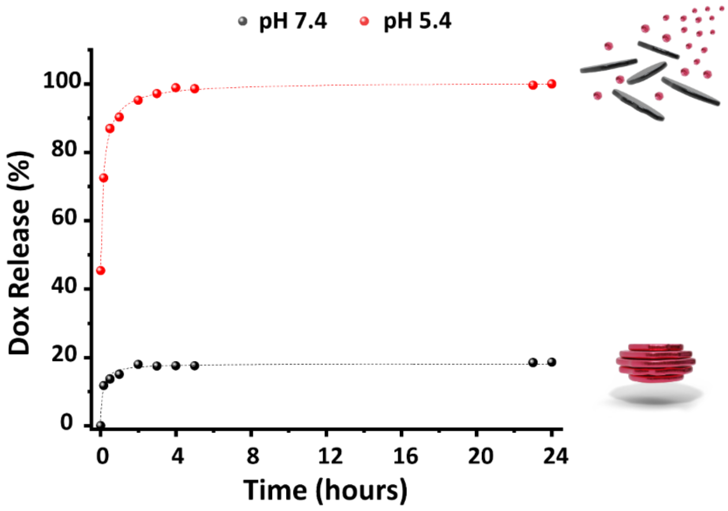

Figure S38. Dox release from Alkyn-nCOF in PBS at pH 7.4 (black) and at pH 5.4 (red). The % of drug release was measured using fluorescence emission.

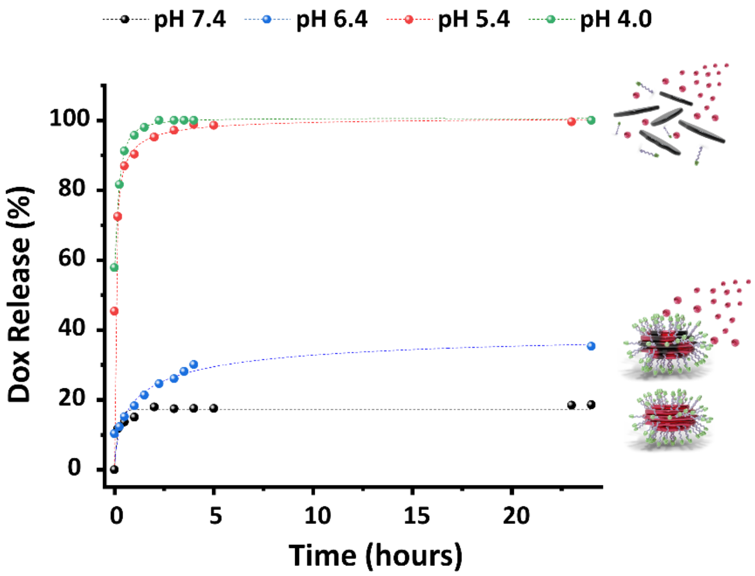

Figure S39. Cumulative release of Dox from Alkyn-nCOF-cRGD@Dox at various pH values. This graph illustrates the cumulative release profile of Dox from Alkyn-nCOF-cRGD@Dox over time at different pH conditions: pH 4.0 (green), pH 5.4 (red), pH 6.4 (blue), and pH 7.4 (black). The inset diagrams on the right show the structure and release mechanism of Alkyn-nCOF-cRGD@Dox. At lower pH values (4.0 and 5.4), Dox is fully released, indicating that the material is responsive to highly acidic environments, which is beneficial for targeting the acidic tumor microenvironment. At a pH of 6.4, which is mildly acidic, there is a significant but controlled release of Dox. In contrast, at a physiological pH (7.4), the release rate is minimal, ensuring negligible premature drug release during circulation. These results demonstrate the potential of Alkyn-nCOF-cRGD@Dox for effective and targeted drug delivery at different pH values, particularly in the acidic tumor microenvironment.

4. *In vitro* biological studies

4.1. Cell culture

Human breast cancer cell lines adenocarcinoma MCF-7 (ATCC HTB-22™) and MDA-MB-231 (ATCC HTB-26) cell lines were cultured in Dulbecco's Modified Eagle's medium (DMEM) supplemented with 10 % fetal bovine serum (FBS) and 1 % penicillin/streptomycin at 5 % CO_2 and 37 °C.

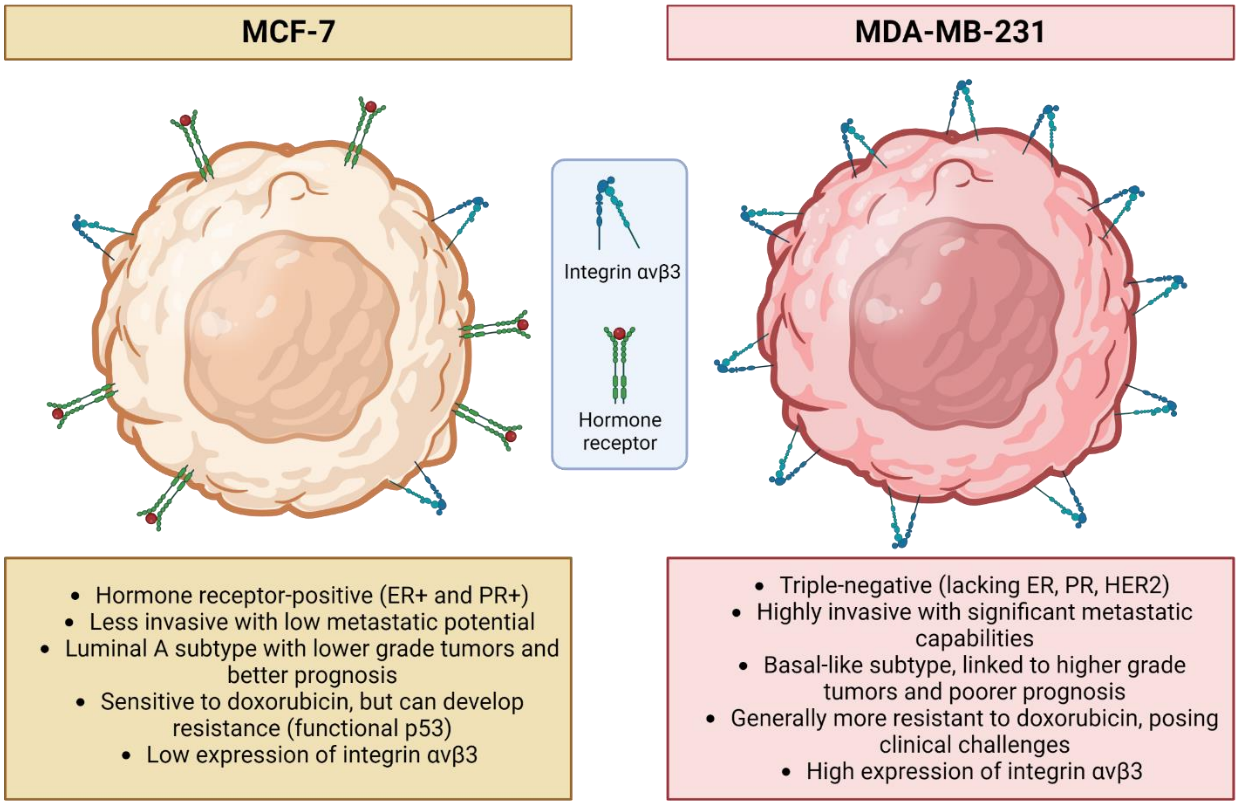

Figure S40. Comparative profile of MCF-7 vs. MDA-MB-231 breast cancer cells: The diagram contrasts the two cell lines, highlighting hormone receptor status, metastatic behavior, subtype, doxorubicin sensitivity, and integrin $\alpha_v\beta_3$ expression, reflecting their distinct prognostic and therapeutic challenges.

4.2. *In vitro* cell viability

Cell viability was assessed using CellTiter-Blue® Cell Viability assay (CTB, Promega). The assay measures the metabolic reduction of a non-fluorescent compound, resazurin, into a fluorescent

product, resofurin, in living cells. As non-viable cells rapidly lose their metabolic activity, the amount of the resofurin product can be used to estimate the number of viable cells following treatment. Once produced, resofurin is released from living cells into the surrounding medium. Thus, the fluorescence intensity of the medium is proportional to the number of viable cells present.

96-well plates were seeded with MCF-7 or MDA-MB-231 (~5,000 cells per well in 100 µL of DMEM) and incubated at 37 °C for 24 hours. The medium was removed and replaced with fresh medium (control) or various concentrations of test compounds and incubated at 37 °C for 48 hours. After that, cells were incubated with 80 µL DMEM and 20 µL of CTB per well for 6 hours at 37 °C. The fluorescence of the resofurin product ($\lambda_{ex/em}$ 560/620) was measured. Untreated wells were used as control.

The percentage of cell viability was calculated using the following formula:

$$\textbf{Viability (\%)} = [(F_{treated} - F_{blank}) / (F_{control} - F_{blank})] \times 100$$

All assays were conducted in triplicate, and the mean IC_{50} ± standard deviation was determined.

4.3. *In vitro* cell toxicity

The IC_{50} values were determined from a plot of percentage cell inhibition against drug concentration (in µM). All assays were conducted in triplicate, and the mean IC_{50} ± standard deviation was determined. The experiment was performed in triplicate.

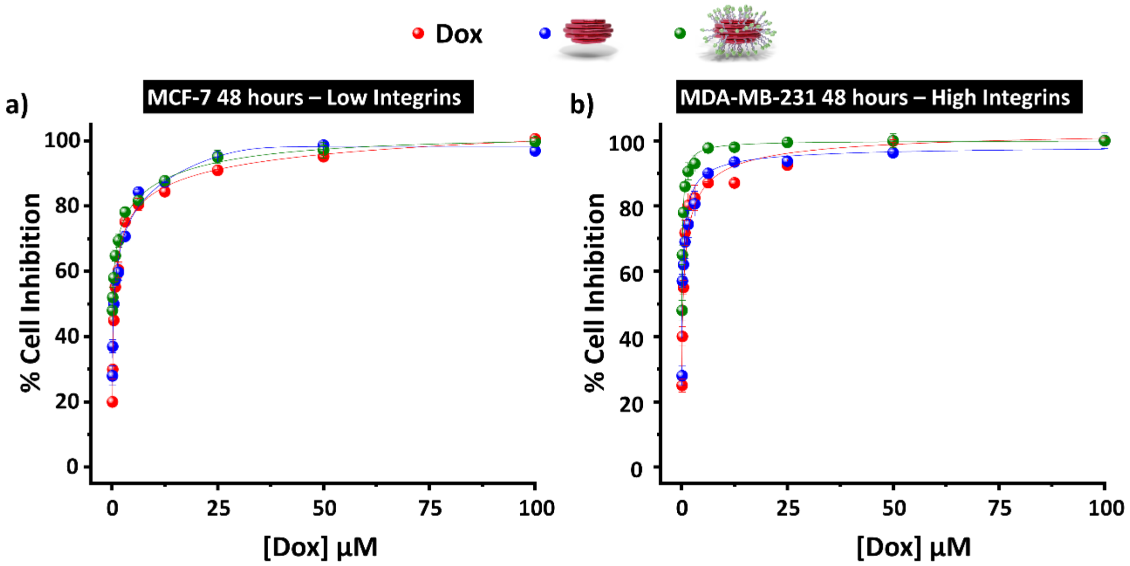

Figure S41. Inhibition of a) MCF-7 and b) MDA-MB-231 cells after 48-hour incubation with Dox alone (red), Alkyn-nCOF@Dox (blue), or Alkyn-nCOF-cRGD@Dox (green) up to [Dox] = 100 µM. Error bars represent standard deviations of triplicate measurements.

4.4. Western blotting

MCF-7 or MDA-MB-231 cells were seeded at 80 % confluency in 6-well plates in complete DMEM and incubated for 24 hours. Then, cells were incubated for 24 hours with no additives (control), Dox alone, Alkyn-nCOF@Dox, or Alkyn-nCOF-cRGD@Dox ([Dox] = 1 µM). For each experiment, the cells were collected and washed with PBS. Cells were lysed in 200 µl of NP40 lysis buffer (150 mM NaCl, 1.0 % NP-40, 50 mM Tris-HCl (pH 8.0)) containing protease inhibitor cocktail (Roche # 11697498001). We then separated 30 µg of protein lysate by 10 % Mini-PROTEAN® TGX™ precast polyacrylamide gels (Biorad # 4561033) and transferred the proteins to nitrocellulose membranes. The membranes were probed using the following antibodies: caspase-3 primary antibody (abcam #ab184787), GAPDH primary antibody (Santa Cruz # sc-32233), Anti-rabbit IgG (Cell Signaling #7074), and Goat Anti-Mouse IgG (Cell Signaling #91196). The protein signals were detected using Clarity Western ECL Substrate (Biorad # 1705060) and ChemiDoc imaging systems (Biorad). Western blot bands were quantified using Fiji-Image J software.

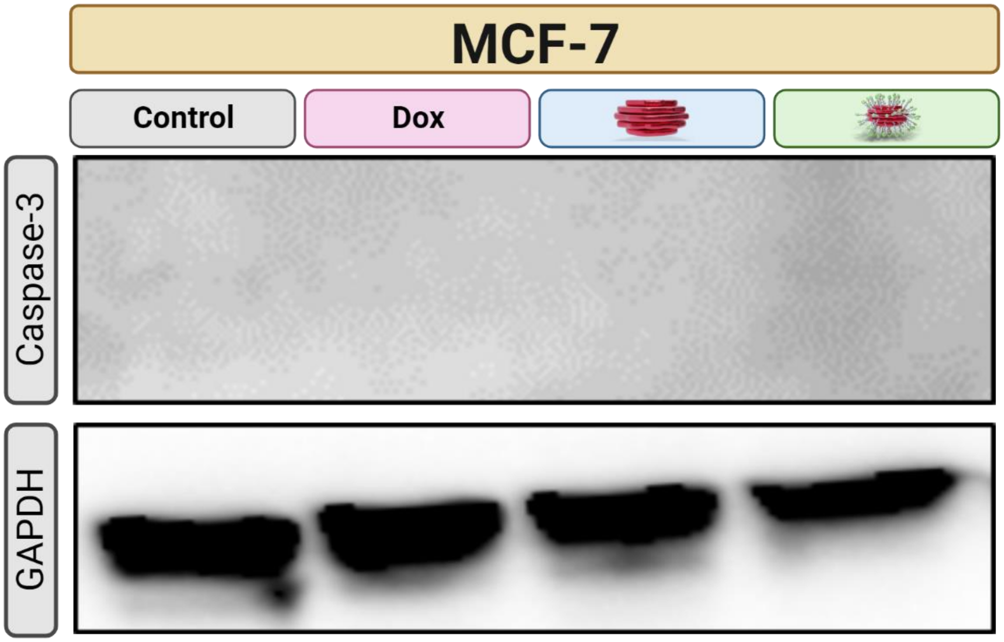

Figure S42. Western blot analysis illustrating Caspase-3 protein expression of MCF-7 cells treated for 24 hours with no additives (control, grey), free Dox (red), Alkyn-nCOF@Dox (blue), and Alkyn-nCOF-cRGD@Dox (green) at a Dox concentration of 1 µM.

4.5. Intracellular distribution study using TEM

For TEM analysis, MCF-7 and MDA-MB-231 cells were seeded in T75 flasks in complete DMEM and incubated for 4 hours with cell-medium alone (control), Alkyn-nCOF@Dox or Alkyn-nCOF-cRGD@Dox ([Dox] = 10 µM) in DMEM. After harvesting, cell pellets were washed twice with PBS. The cells were cryo-fixed within a few milliseconds at a pressure of 2000 bar under liquid nitrogen using a high-pressure freezer (Leica Microsystems, Germany). After freezing, the sample pod was released automatically into a liquid nitrogen bath. While still in liquid nitrogen, the sample carrier was separated from the specimen pod using precooled fine-tipped tweezers and transferred to the cryo-transfer storage box for the flat specimen carrier, where the samples were stored in preparation for freeze substitution. Freeze substitution was performed using an automatic freeze substitution (AFS) unit (Leica EM AFS2, Heerbrugg, Switzerland) in a 10 mL solution of cold, dry absolute acetone (v/v) containing 1 % osmium tetroxide (w/v), 0.5 % uranyl acetate (w/v) and 5 % distilled water (v/v). The AFS unit was slowly warmed from −90 °C to 0 °C (2 °C/hour), with the

temperature being held at both −60 °C and −30 °C for 8 hours. Samples were transferred to room temperature in a closed container to prevent condensation, rinsed with absolute acetone (3 × 5 minutes), and infiltrated with 30, 60, and 100 % Epon resin for 3 hours each. Epon was exchanged, and individual samples were embedded in 1 mL Eppendorf® lids for 24 hours at 60 ˚C. Finally, the samples were sectioned with an ultra-microtome at room temperature using a diamond knife, and the ultrathin sections were examined under TEM (Talos F200X STEM). The experiment was performed in triplicate.

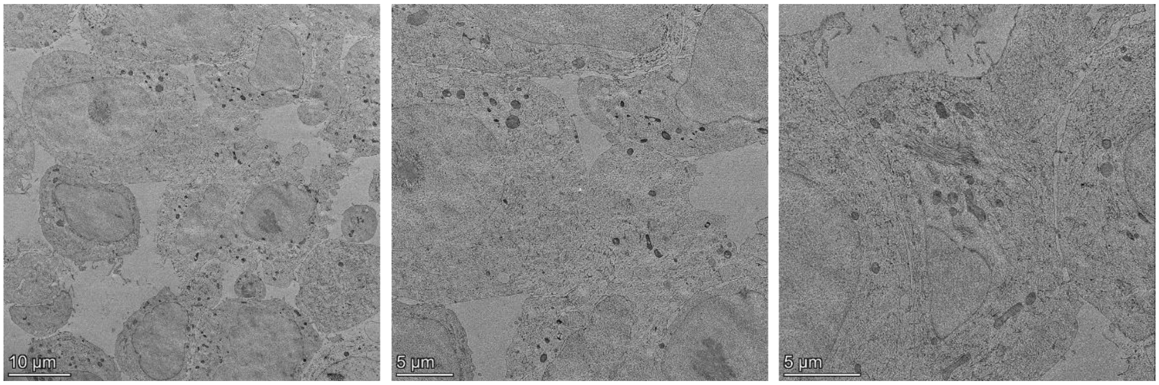

Figure S43. TEM images of MCF-7 cells.

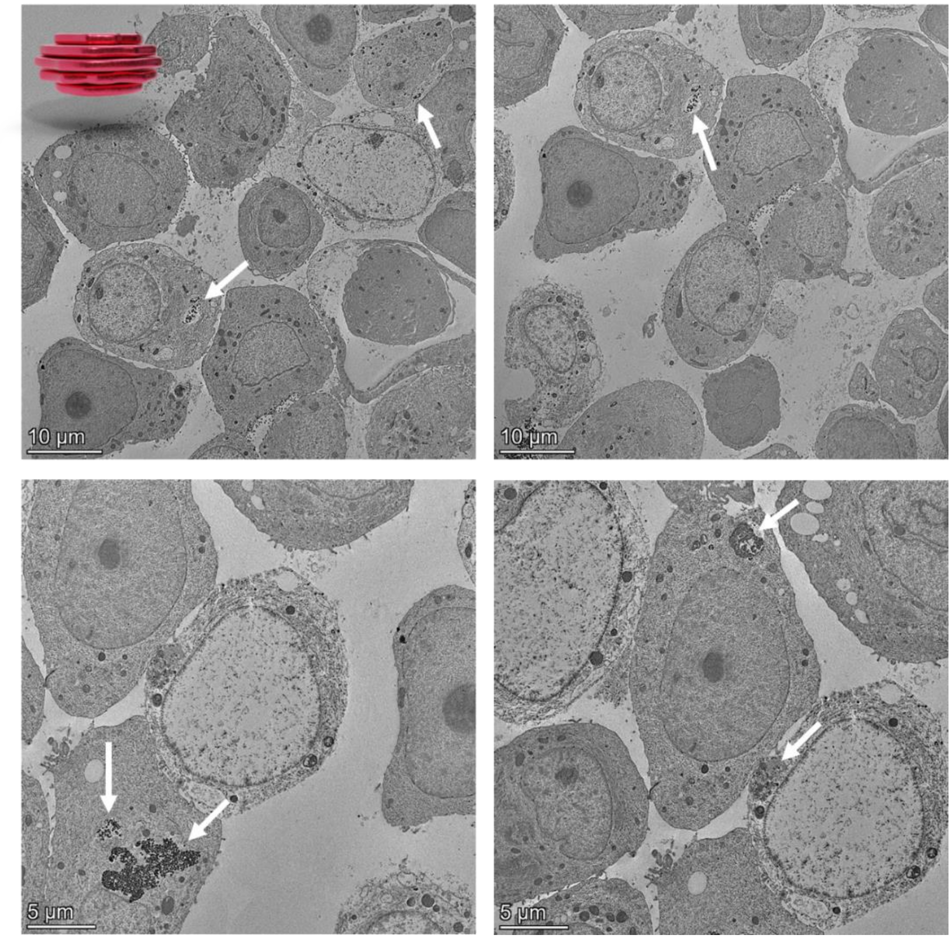

Figure S44. TEM images of MCF-7 cells treated with Alkyn-nCOF@Dox ([Dox] = 10 µM) for 4 h at various magnifications. White arrows show nanoparticle uptake in cells and their transit in the cytoplasm.

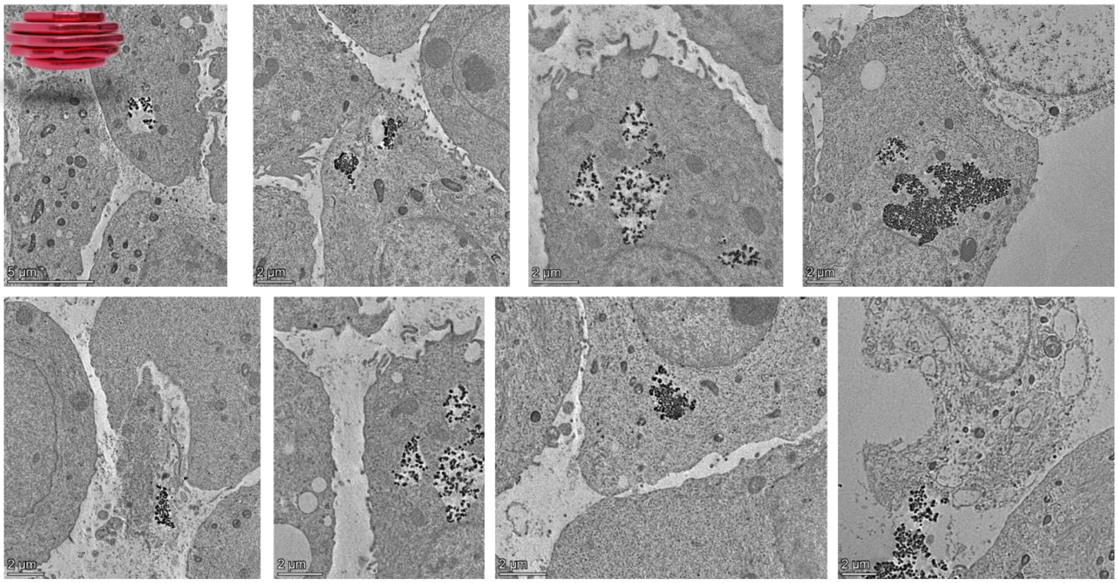

Figure S45. TEM images of MCF-7 cells treated with Alkyn-nCOF@Dox ([Dox] = 10 μM) for 4 h at higher magnifications.

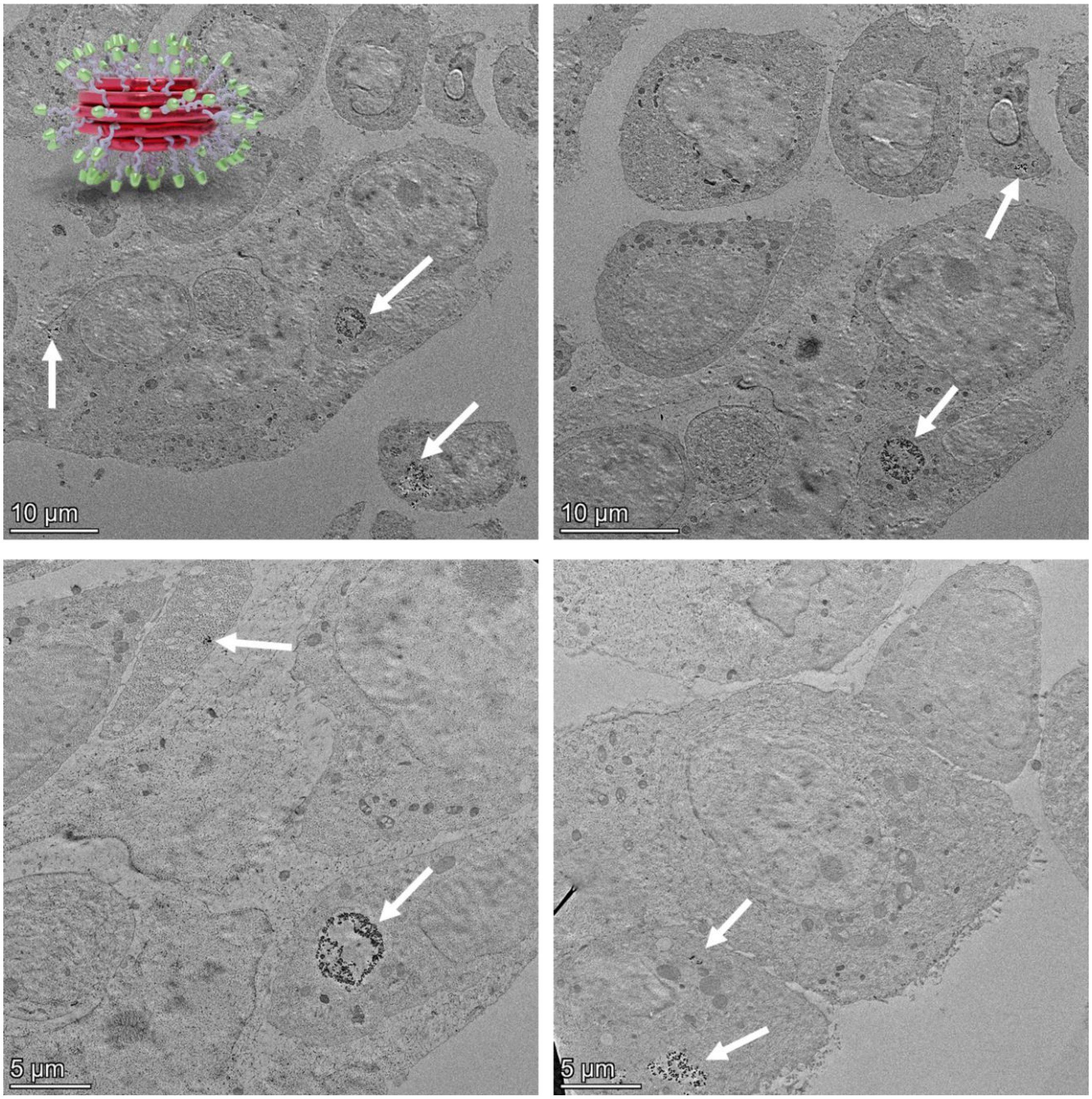

Figure S46. TEM images of MCF-7 cells treated with Alkyn-nCOF-cRGD@Dox ([Dox] = 10 µM) for 4 h at various magnifications. White arrows show nanoparticle uptake in cells and their transit in the cytoplasm.

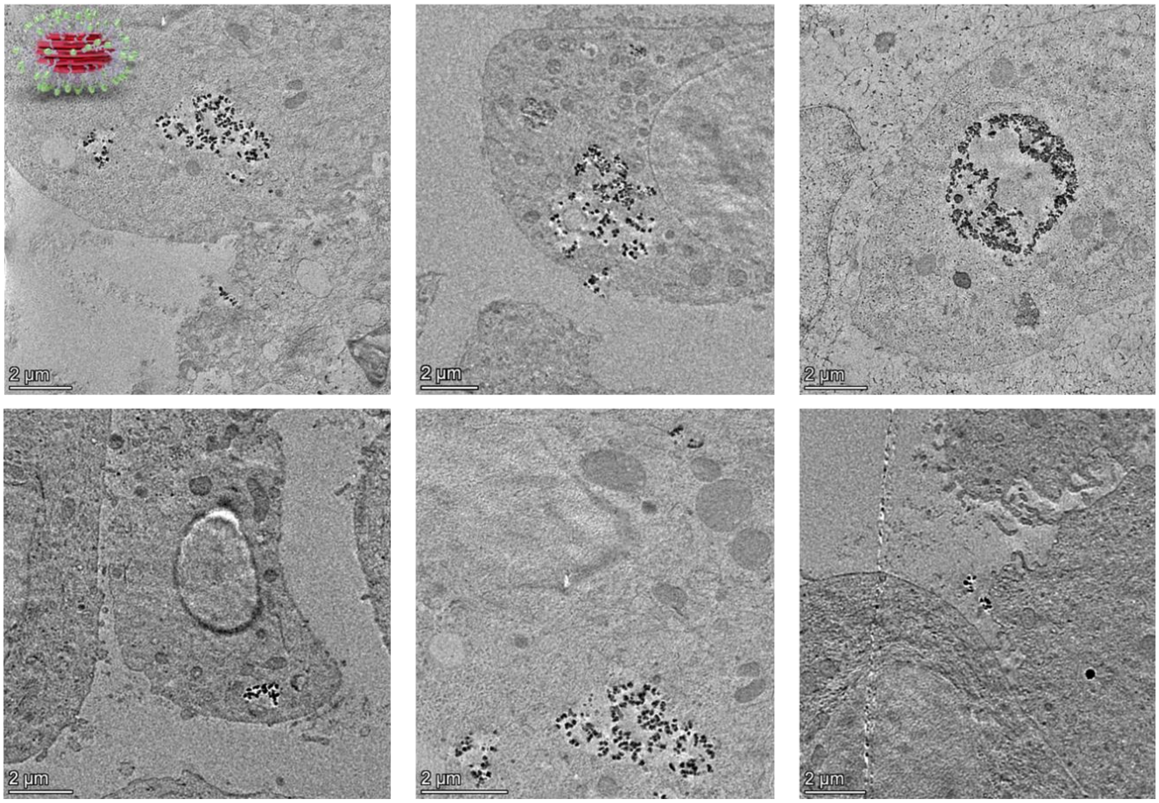

Figure S47. TEM images of MCF-7 cells treated with Alkyn-nCOF-cRGD@Dox ([Dox] = 10 μM) for 4 h at higher magnifications.

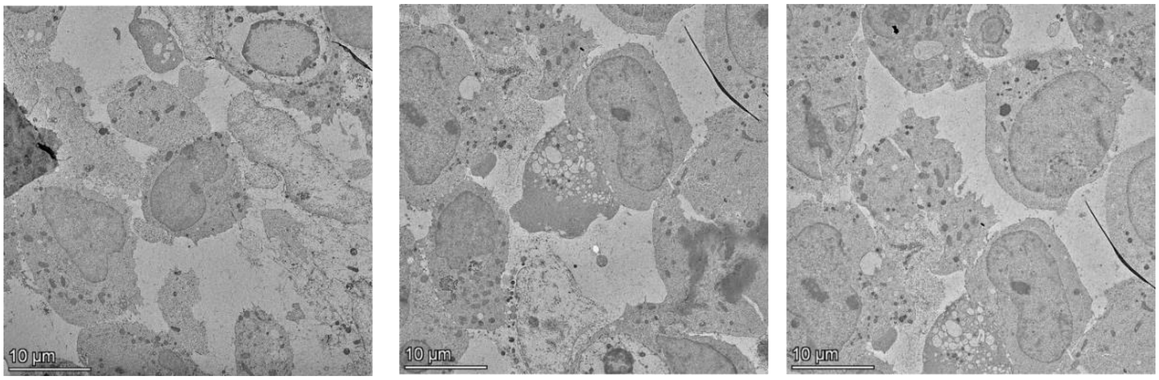

Figure S48. TEM images of MDA-MB-231 cells.

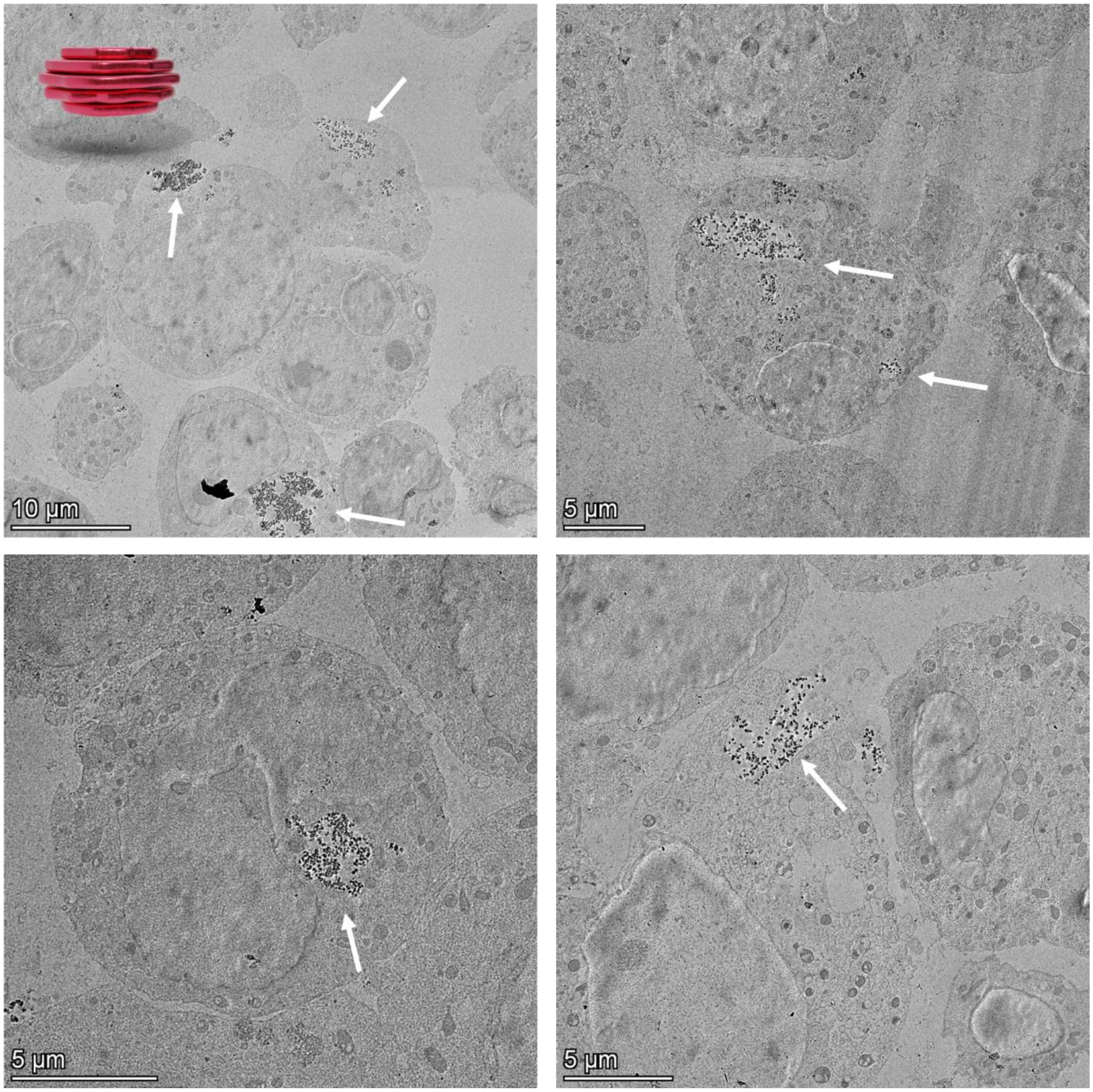

Figure S49. TEM images of MDA-MB-231 cells treated with Alkyn-nCOF@Dox ([Dox] = 10 µM) for 4 h at various magnifications. White arrows show nanoparticle uptake in cells and their transit in the cytoplasm.

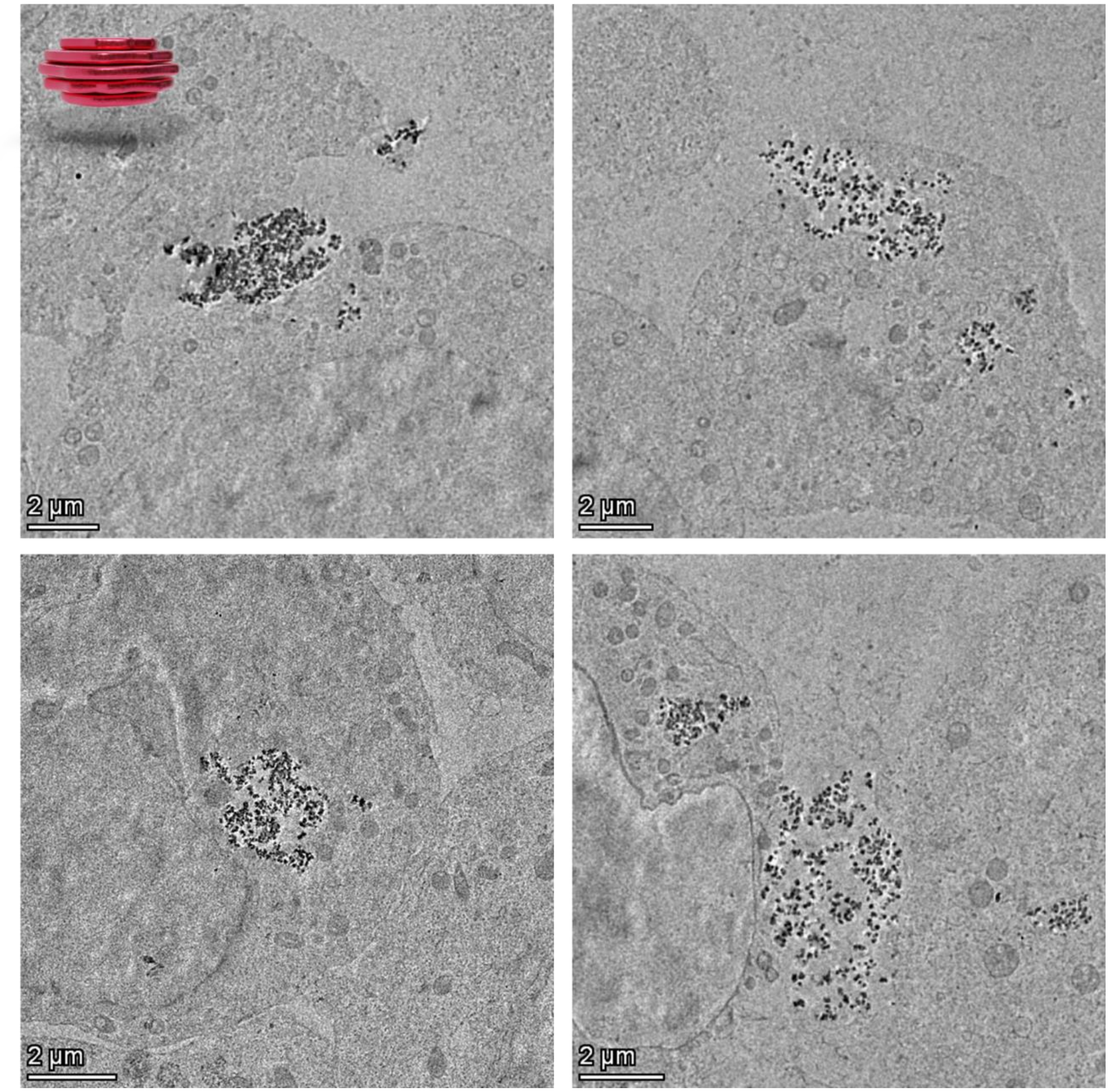

Figure S50. TEM images of MDA-MB-231 cells treated with Alkyn-nCOF@Dox ([Dox] = 10 µM) at higher magnifications.

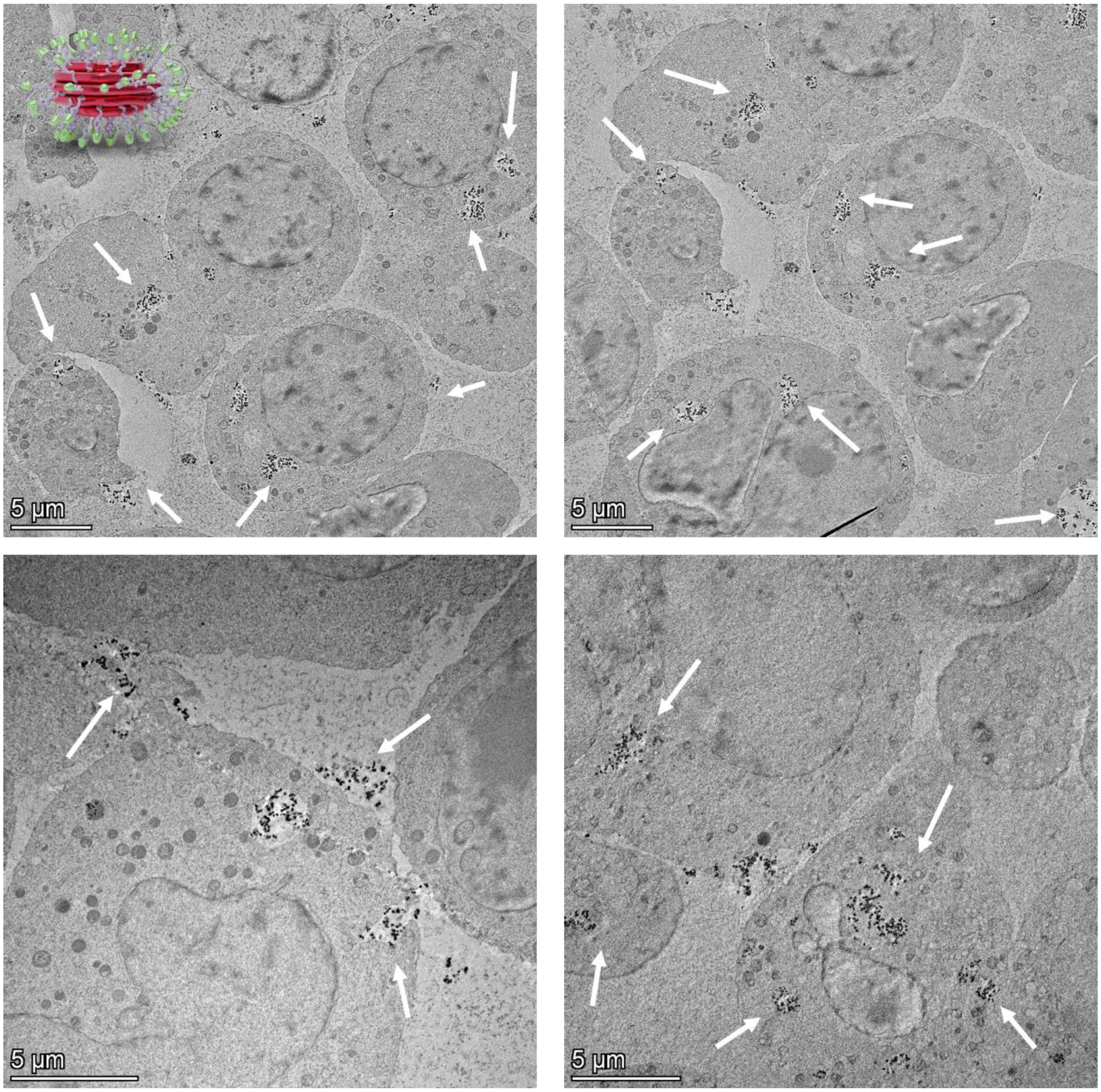

Figure S51. TEM images of MDA-MB-231 cells treated with Alkyn-nCOF-cRGD@Dox ([Dox] = 10 µM) for 4 h at various magnifications. White arrows show nanoparticle uptake in cells through integrin-mediated endocytosis and their transit in the cytoplasm.

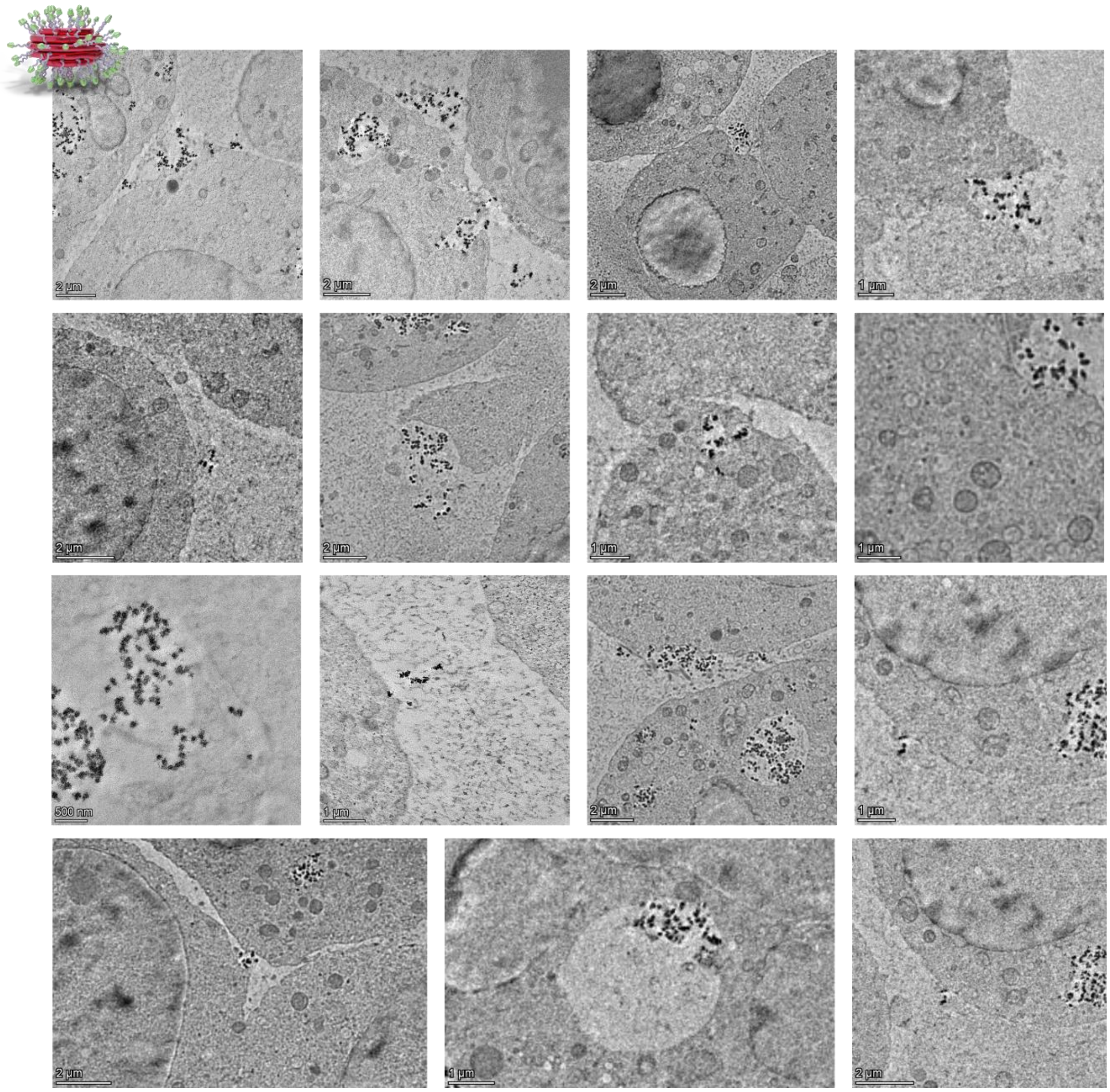

Figure S52. TEM images of MDA-MB-231 cells treated with Alkyn-nCOF-cRGD@Dox ([Dox] = 10 µM) for 4 h at higher magnification displaying uptake in cells through integrin-mediated endocytosis and their transit in the cytoplasm.

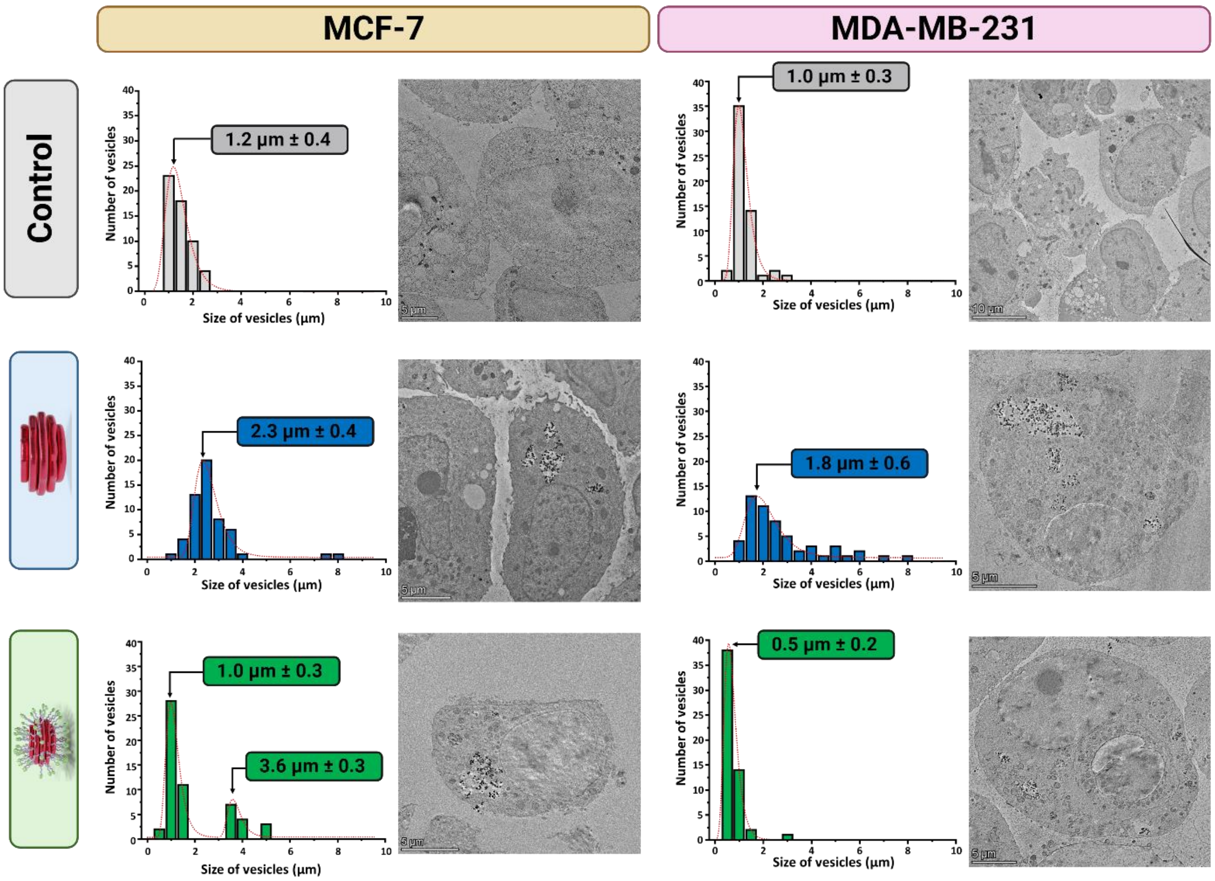

Figure S53. Transmission electron microscopy (TEM) images and corresponding size distribution histograms of endocytotic vesicles in MCF-7 and MDA-MB-231 breast cancer cells. Cells were treated with control (no treatment), Alkyn-nCOF@Dox, and Alkyn-nCOF-cRGD@Dox to assess nanoparticle internalization and vesicle formation. Control MCF-7 cells show an average vesicle size of 1.2 µm ± 0.4, while Alkyn-nCOF@Dox and Alkyn-nCOF-cRGD@Dox treatments result in average vesicle sizes of 2.3 µm ± 0.4 and 1.0 µm ± 0.3, respectively. For MDA-MB-231 cells, control vesicles measure 1.0 µm ± 0.3, with Alkyn-nCOF@Dox treatment increasing the average vesicle size to 1.8 µm ± 0.6 and Alkyn-nCOF-cRGD@Dox treatment decreasing it to 0.5 µm ± 0.2. The histograms reflect the distribution of vesicle sizes observed in the TEM images, indicating the effect of nanoparticle treatment on endocytic vesicle dynamics within these cell lines.

4.6. LDH release experiment

To assess the ability of Alkyn-nCOF@Dox, or Alkyn-nCOF-cRGD@Dox to disrupt the plasma membrane of the cancer cells the detection of lactate dehydrogenase (LDH) release from treated MCF-7 and MDA-MB-231 cells was performed using LDH-Glo™ Cytotoxicity Assay (Promega #J2380). cells were incubated for 24 hours with no additives (control), Dox alone, Alkyn-nCOF@Dox, or Alkyn-nCOF-cRGD@Dox ([Dox] = 1 µM). After 24-hours incubation, 5 µL of the medium was diluted in 50 µL of LDH storage buffer and transferred into a new 96 well plates, and then, 50 µL of LDH detection reagent was added. Luminescence was recorded After 30 min of incubation using a Cytation 5 multimode reader (Biotek).

4.7. *In vitro* internalization study by confocal microscopy

MCF-7 or MDA-MB-231 cells were seeded on sterile coverslips in complete DMEM and incubated for 24 hours. Then, cells were incubated for 24 hours with no additives (control), Dox alone, Alkyn-nCOF@Dox, or Alkyn-nCOF-cRGD@Dox ([Dox] = 10 µM). Then, for each experiment, the cells were washed with PBS, fixed with paraformaldehyde solution (3.7 %) for 10 minutes, and washed thrice with PBS. The coverslips were then fixed onto a microscope slide with mounting media. Samples were analyzed using confocal microscopy. Each sample was assayed in duplicate, and the experiment was repeated 3 times.

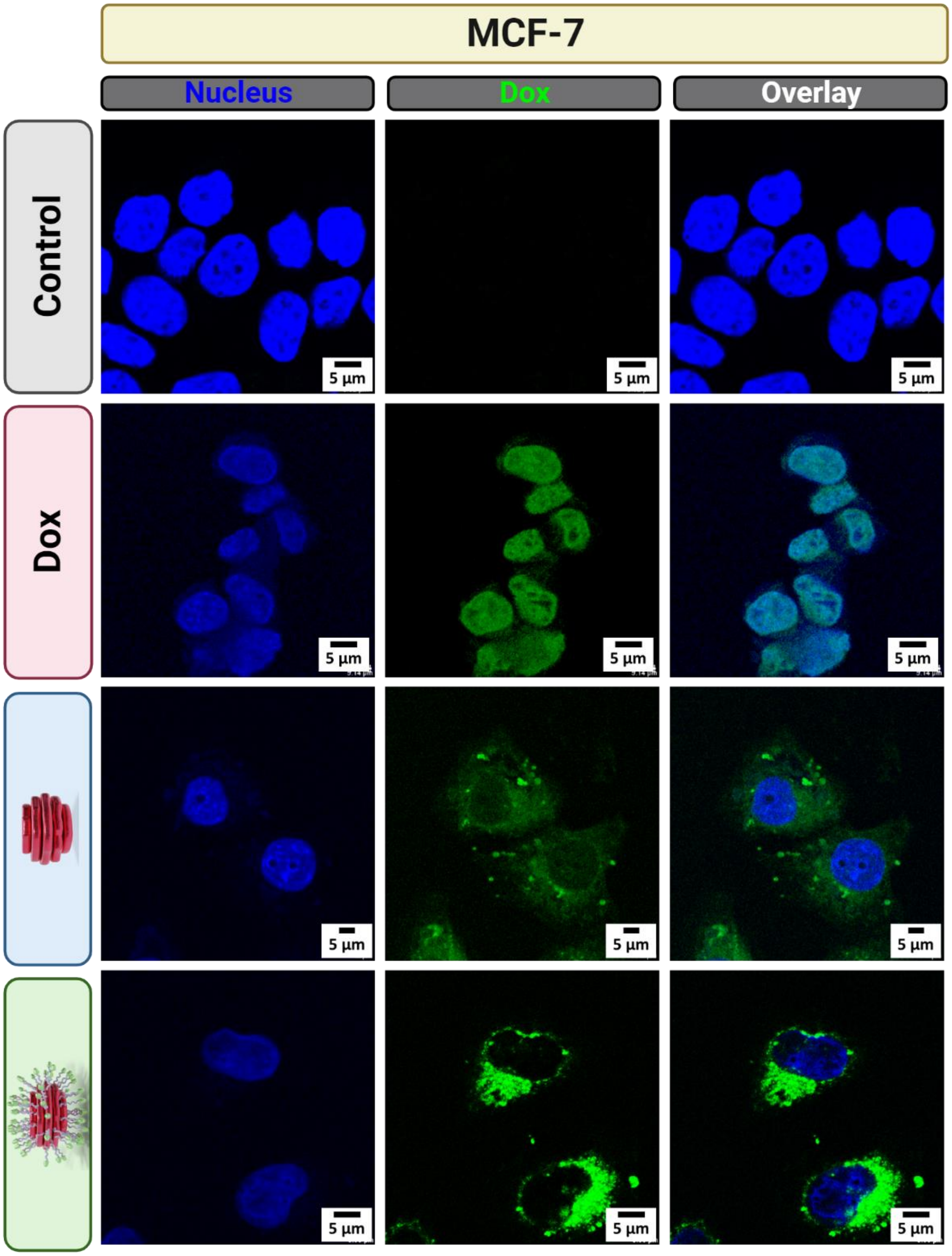

Figure S54. Confocal images of MCF-7 cells incubated for 24 hours with no additives (control), Dox alone, Alkyn-nCOF@Dox or Alkyn-nCOF-cRGD@Dox ([Dox] = 10 µM). Nucleus were labeled using DAPI on all images. DAPI channel (λ_{ex} = 400 nm), Dox channel (λ_{ex} = 488 nm).

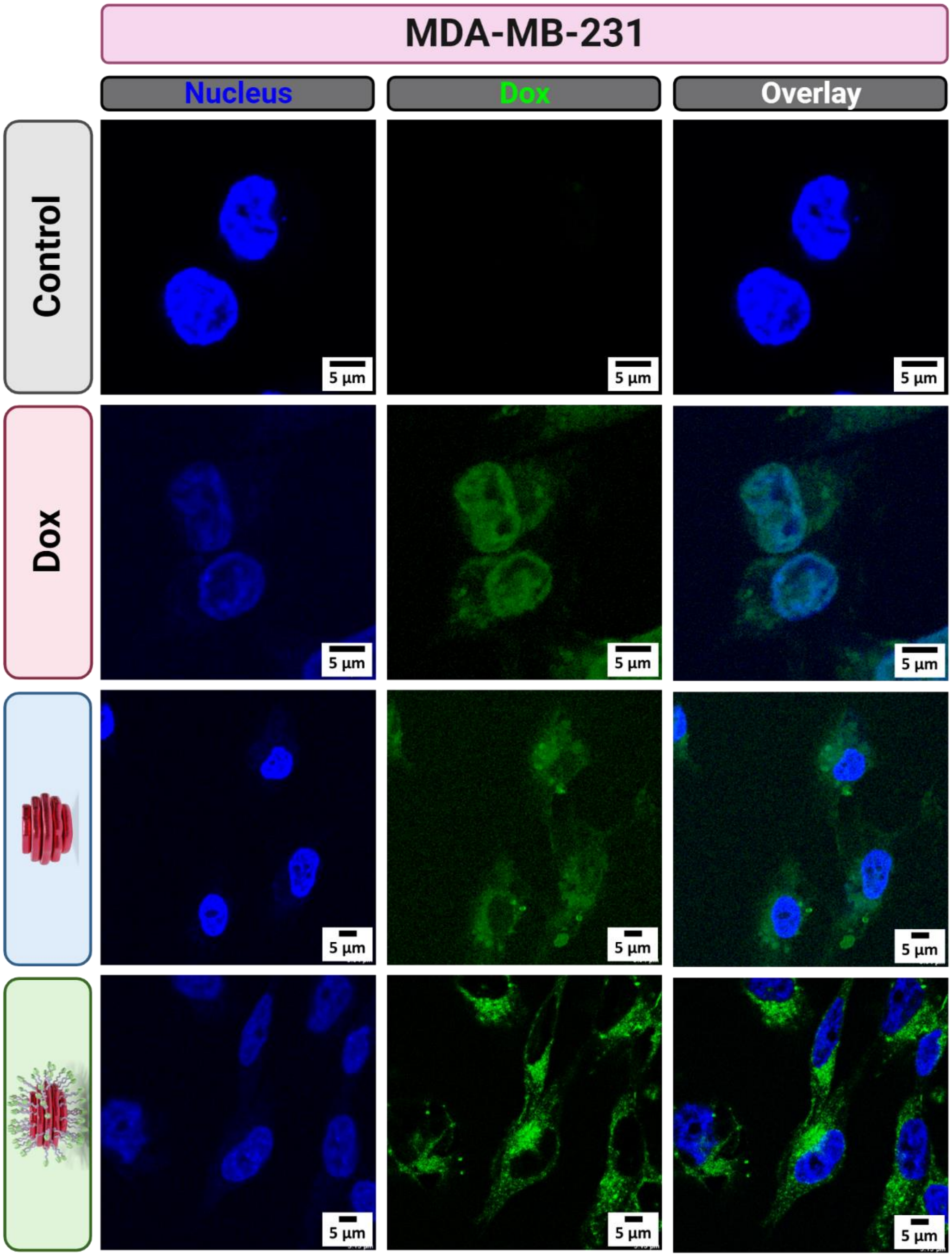

Figure S55. Confocal images of MDA-MB-231 cells incubated for 24 hours with no additives (control), Dox alone, Alkyn-nCOF@Dox or Alkyn-nCOF-cRGD@Dox ([Dox] = 10 µM). Nucleus were labeled using DAPI on all images. DAPI channel (λ_{ex} = 400 nm), Dox channel (λ_{ex} = 488 nm).

4.8. Flow cytometry analysis

MCF-7 or MDA-MB-231 cells were grown in petri dishes to a density of 100,000 cells per mL in DMEM for 24 hours. Cells were incubated for 24 hours with no additives (control), Alkyn-nCOF@Dox or Alkyn-nCOF-cRGD@Dox ([Dox] = 1 µM). Dox uptake was measured with an Attune Nxt flow cytometer (Thermo Fisher) using BL1 channel (Blue (488nm) laser and 530/30nm filter). The experiment was performed in triplicate.

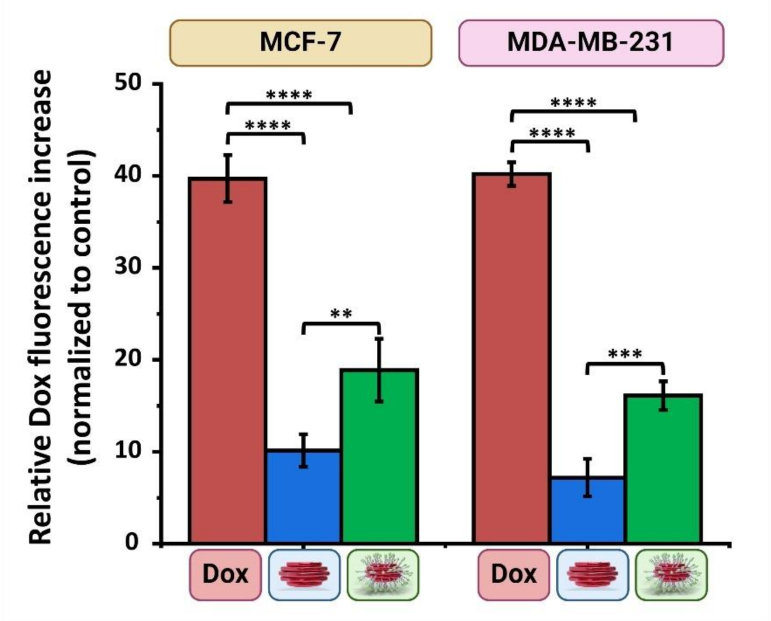

Figure S56. Differential uptake of doxorubicin (Dox) in MCF-7 and MDA-MB-231 breast cancer cell lines. This bar graph compares the relative fluorescence intensity, indicative of Dox accumulation, between the two cell lines treated with free Dox (red), Alkyn-nCOF@Dox (blue), and Alkyn-nCOF-cRGD@Dox (green). Statistical significance is denoted as ** $p < 0.05$, ** $p < 0.01$, *** $p < 0.001$* and ***** $p < 0.0001$*.

4.9. *In vitro* organelle co-localization study by confocal microscopy

MDA-MB-231 cells were seeded on sterile coverslips in complete DMEM and incubated for 24 h. Cells were incubated for 4 hours with no additives (control), Dox alone, Alkyn-nCOF@Dox, or Alkyn-nCOF-cRGD@Dox ([Dox] = 10 µM). The cells were stained with organelles markers to understand the internalization of the different Dox compositions. Cells were incubated for 30 min either with LysoTracker™ 647 Deep Red (labeling lysosomes and endosomes), MitoTracker™ Deep Red FM (labeling mitochondria), and CellMask™ 647 Deep Red (labeling membrane), followed by three cycles of PBS washing. Then, for each experiment, the cells were fixed with formaldehyde solution (3.7 %) for 10 min, followed by washing thrice with PBS. The cells were kept for 5 min in the PBS during washing cycles. ProLong Live Antifade Reagent containing DAPI to label the nucleus was added to suppress photo-bleaching and preserve the fluorescent signals. The coverslips were then fixed onto a microscope slide. The intracellular internalization of the different Dox compositions was observed using confocal microscopy (Olympus FV1000MPE) measuring the fluorescence signal of the Dox (with excitation/emission of 488/560 nm) in cells as well as the fluorescence emission from the 4 organelle markers labeling the plasmic membrane, the lysosomes, the mitochondria (with excitation/emission of 647/668 nm) as well as the nucleus (with excitation/emission of 400/461 nm).

5. *In vivo* biological studies

All animal experiments were conducted in accordance with the policies of the New York University Institutional Animal Care and Use Committee (IACUC). Athymic NU/J nude mice (4-6 weeks old, about 20 g) were fed with normal conditions of 12 h light and dark cycles and given access to food and water *ad libitum*. All animal experimentation was approved by the Institutional Animal Care and Use Committee of NYUAD.

5.1. Biodistribution study on healthy animals

The mice were randomized into 3 groups (n=3) and treated with 0.2 mL of saline (control), Alkyn-nCOF, and Alkyn-nCOF-cRGD at a [NP] = 20 mg/kg dose (200 µL) via intraperitoneal injection. Mice were sacrificed 24 hours post-injection, and major organs, including liver, kidney, heart, and spleen, were harvested. Each organ was rinsed with PBS and immersed in lysing buffer, and the tissues were homogenized using a PowerLyzer 24 Homogenizer. 100 µl of the soupy solution was loaded in 12 wells in 96-well plate with clear bottoms, and the UV-vis intensity was measured using a microplate reader and compared to untreated (control) animals.

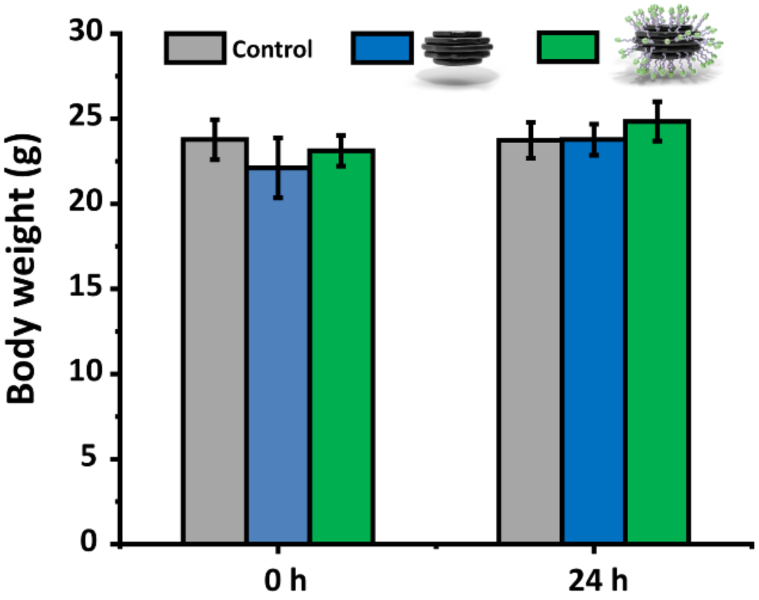

Figure S57. Body weight of healthy animals at baseline (t=0) and after 24 hours. The control group is represented in grey, while the groups treated with intraperitoneal injections of Alkyn-nCOF and Alkyn-nCOF-cRGD nanoparticles are shown in blue and green, respectively. Each bar corresponds to the average weight of three subjects (n=3), indicating no significant change in body weight post-treatment within the 24-hour observation period.

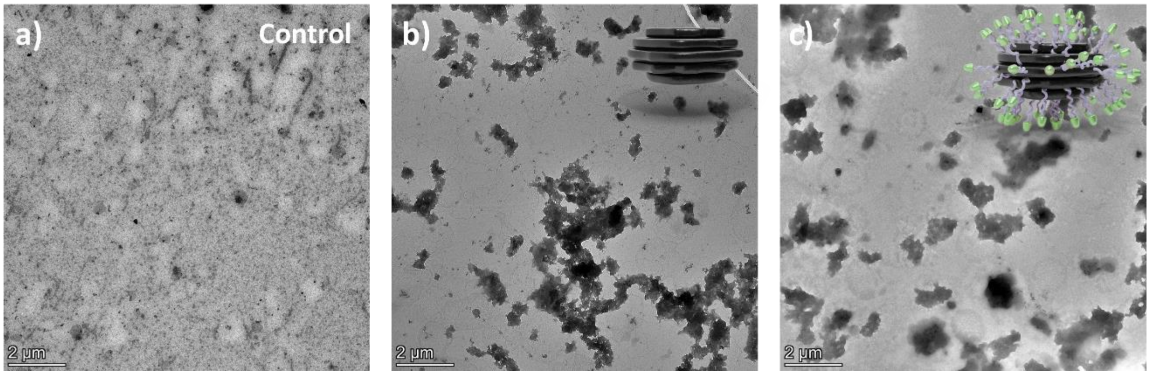

Figure S58. TEM images of the soupy solution of livers treated with a) no additives (control), b) Alkyn-nCOF, and c) Alkyn-nCOF-cRGD after homogenization. Livers of animals treated with Alkyn-nCOF and Alkyn-nCOF-cRGD display the presence of nanoparticles as dark materials.

5.2. Tumor model establishment

The MDA-MB-231 cancer model was employed as an example of aggressive triple-negative breast cancer to evaluate the therapeutic effect of the different Dox formulations. 5×10^6 MDA-MB-231 cells in 200 µL of DMEM medium were injected subcutaneously into the right axillary region of nude mice. The mice were utilized in subsequent experiments after the tumor size had reached approximately 75-100 mm^3.

5.3. Biodistribution study on tumor-bearing mice

To quantify the amount of Dox in tissues, tumor-bearing mice were divided into three groups at random (n = 3) and injected with 0.2 mL of saline (control), Alkyn-nCOF@Dox, and Alkyn-nCOF-cRGD@Dox ([Dox] = 5 $mg.kg^{-1}$, [NP] = 10 $mg.kg^{-1}$, 200 µL) via intraperitoneal injection.

The Dox signal was tracked in live animals using an IVIS Spectrum optical imaging system (PerkinElmer/Revvity). *In vivo,* real-time fluorescence imaging analysis was used to qualitatively evaluate the tissue distribution of Dox generated by analyzing fluorescent, whole-body dorsal images of the mice using IVIS at the end of the treatment. The anesthetized mice were positioned on the IVIS heated stage (37°C) in a left lateral decubitus orientation. The fluorescent scans were performed 24h post i.p. injection using IVIS Spectrum imaging system *in vivo* imaging system and employing a set of excitation and emission filters centered around excitation and emission peaks of Dox (470nm/560nm). Images were analyzed using Living Image software employing a spectral unmixing approach to distinguish the specific fluorescent signal emitted by Dox from the inherent fluorescence background. To accomplish this, the autofluorescence background was quantified from tumors in untreated control mice.

After 24 post-injection, animals were sacrificed, and the tumors and major organs, including the heart, liver, spleen, and kidney, were harvested for *ex vivo* imaging on the IVIS system. Each organ was rinsed with PBS, and the fluorescence intensity was detected. Dox fluorescent images were taken for all organs and analyzed with Living Image software. Fluorescence values are represented as a percentage of total fluorescence. Treatment groups were compared for

differences in Dox accumulation via one-way ANOVA with post hoc Tukey HSD using GraphPad PRISM software.

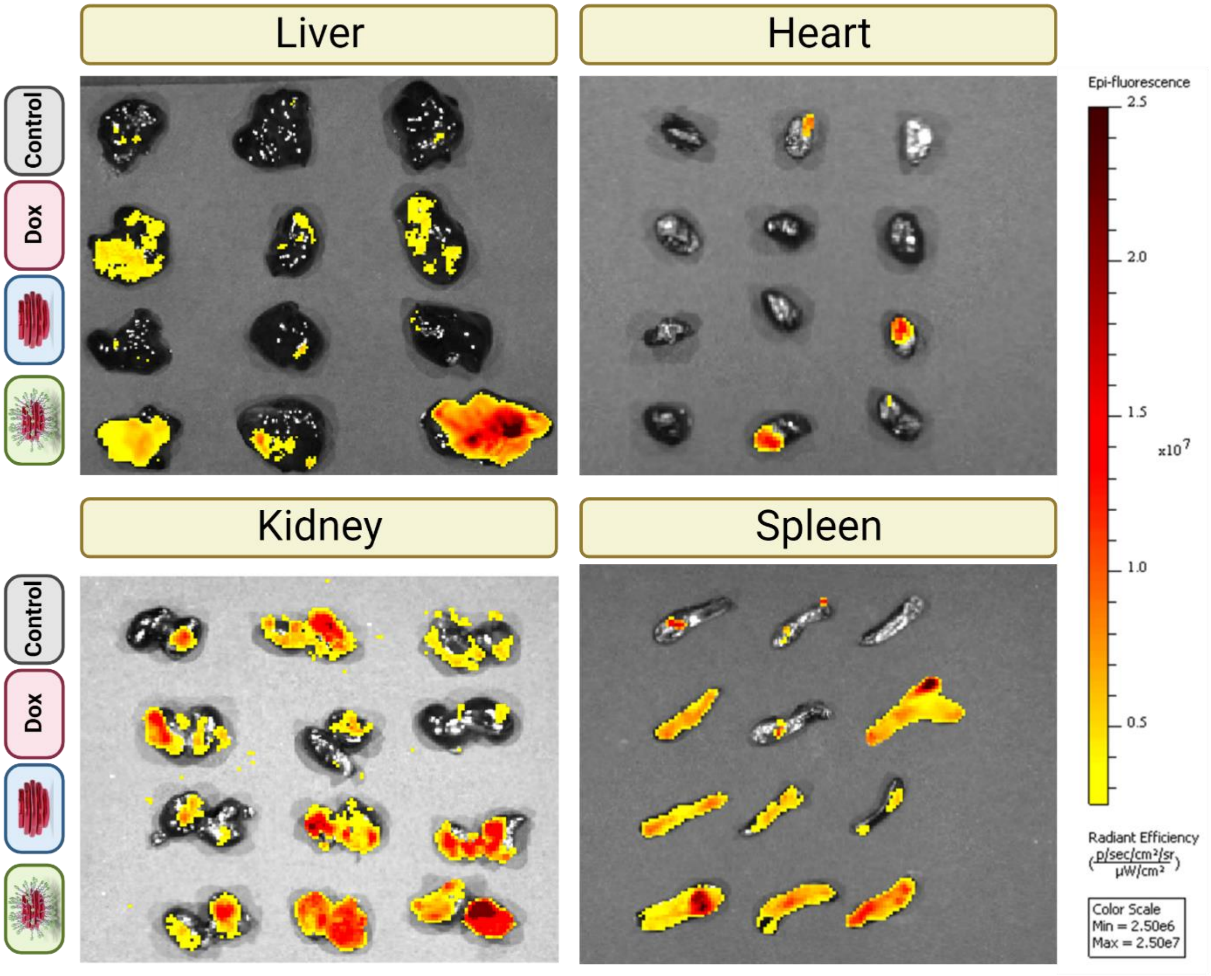

Figure S59. *Ex vivo* IVIS images of vital organs (liver, heart, kidney, spleen) depicting the distribution of Dox fluorescence 24 hours after treatment with PBS (control), free Dox, Alkyn-nCOF@Dox, or Alkyn-nCOF-cRGD@Dox. Dose: [Dox] = 5 mg/kg, [NP] = 10 mg/kg, 200 µL.

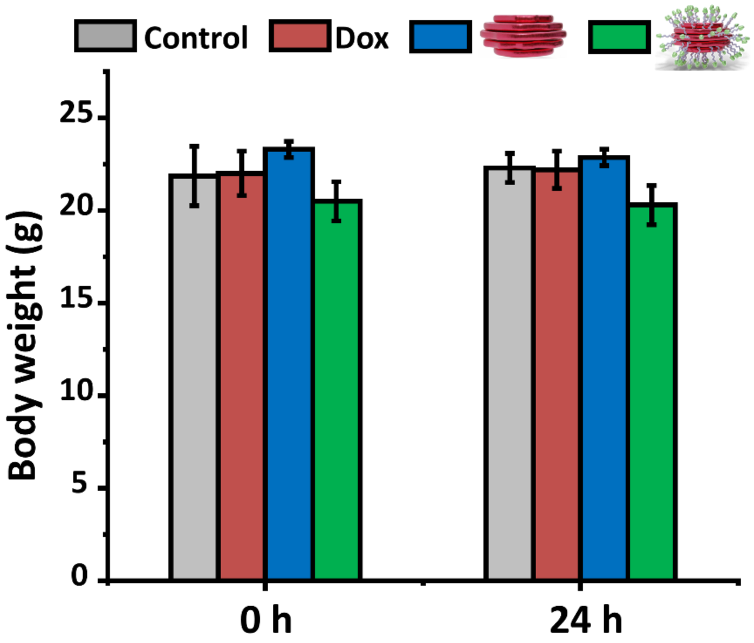

Figure S60. Body weight of tumor-bearing mice at baseline (t=0) and after 24 hours. The control group is represented in grey, while the groups treated with intraperitoneal injections of free Dox, Alkyn-nCOF and Alkyn-nCOF-cRGD nanoparticles are shown in red, blue and green, respectively. Each bar corresponds to the average weight of three subjects (n=3), indicating no significant change in body weight post-treatment within the 24-hour observation period.

5.4. *In vivo* antitumor efficacy

To assess the antitumor effect, tumor-bearing mice were randomly divided into four groups (Control n=11, Doxorubicin n=15, Alkyn-nCOF@Dox and Alkyn-nCOF-cRGD@Dox n=14) and injected with 0.2 mL of saline, Dox alone ([Dox] = 5 mg.kg^{-1}), Alkyn-nCOF@Dox and Alkyn-nCOF-cRGD@Dox ([Dox] = 5 mg.kg^{-1}, [NP] = 10 mg.kg^{-1}, 200 µL) via intraperitoneal injection every 2 days for 20 days. Animal weight and tumor volume was measured before each injection. Tumor size was monitored via caliper measurement every 2 days, and the tumor volume was estimated using the Eq (1):

$$V=0.5\times length\times(width)^2 \quad (1)$$

On day 20, the mice were sacrificed, and the tumor mass and main organs were harvested, weighed, and photographed.

Rationale behind our chosen doses:

1. **Selection of Doxorubicin Dose ([Dox] = 5 mg/kg)**: The dose of doxorubicin was chosen based on established therapeutic doses used in preclinical studies for triple-negative breast cancer models, which typically range from 4 to 6 mg/kg. This dosage effectively balances anticancer efficacy with the minimization of systemic toxicity, as higher doses are associated with increased cardiotoxicity and other adverse effects. Our dose is aligned with those reported in the literature to facilitate comparisons and ensure that our results are relevant within the existing research framework.[28]
2. **Selection of Nanoparticle Dose ([NP] = 10 mg/kg)**: The dose for Alkyn-nCOF@Dox and Alkyn-nCOF-cRGD@Dox was determined to ensure an adequate concentration of nCOFs for effective tumor targeting while maintaining safety. This dose was selected based on preliminary toxicity studies, which showed that this concentration does not produce significant systemic toxicity in healthy mice. Additionally, the 10 mg/kg dose was effective in achieving a therapeutic concentration of the nCOFs in the tumor tissue, as confirmed by biodistribution studies.
3. **Volume of Administration (200 µL)**: The administration volume was chosen to ensure that the dosing could be delivered in a consistent, controlled manner while minimizing discomfort and

stress to the animals. A volume of 200 µL is within the recommended range for intravenous and intraperitoneal injections in mice and is small enough to avoid causing undue pressure or volume overload, which can lead to complications or variability in drug absorption.

4. **Justification for Combined Dosing**: The combined administration of free Dox, Alkyn-nCOF@Dox, and Alkyn-nCOF-cRGD@Dox at these specified doses allows for a direct comparison of efficacy and safety profiles across different delivery methods. This approach is intended to demonstrate the enhanced targeting and therapeutic benefits of our COF-based systems compared to free drug administration.

These doses were chosen to maximize the therapeutic potential while ensuring the safety of the animal subjects. By employing these specific dosing parameters, we aim to provide a clear, controlled assessment of the comparative efficacy and safety of our novel drug delivery systems against a well-established benchmark of free doxorubicin.

5.5. *In vivo* biodistribution study using IVIS Spectrum

Following the methodology described in section 5.3, the quantification of Dox signal on day 20 was conducted utilizing the IVIS Spectrum optical imaging system (PerkinElmer/Revvity). Subsequently, *in vivo* fluorescence imaging was employed to qualitatively assess the tissue distribution of Dox at the conclusion of the 20-day treatment period, involving intraperitoneal injections every 2 days. Fluorescent imaging took place 24 hours after the last intraperitoneal injection utilizing the IVIS Spectrum *in vivo* imaging system and the same combination of filters. Anesthetized mice were positioned on a heated plate (37°C) in a ventral decubitus orientation within the IVIS Spectrum chamber, allowing for fluorescence imaging with a dorsal view. The fluorescent signals were then analyzed using a similar spectral unmixing approach.

After 20 days of treatment, all the tumor-bearing mice were sacrificed, and the tumor and major organs, including the heart, liver, spleen, lung, and kidney, were harvested for *ex vivo* imaging on the IVIS Spectrum system. Each organ was rinsed with PBS, and the fluorescence intensity was detected. Dox fluorescent images were taken for all organs and analyzed with Living Image software. Fluorescence values are represented as a percentage of total fluorescence. Treatment

groups were compared for differences in Dox accumulation via one-way ANOVA with post hoc Tukey HSD using GraphPad PRISM software.

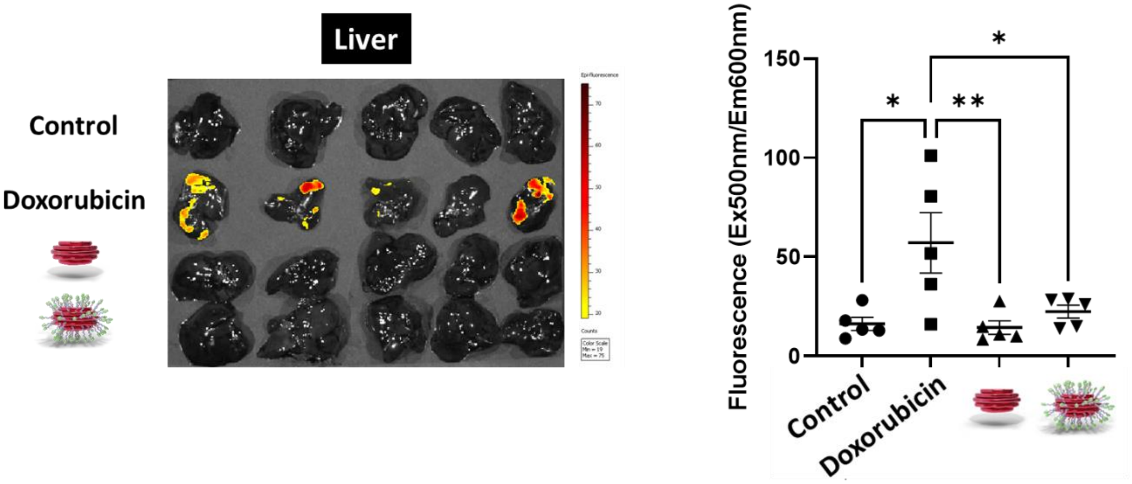

Figure S61. *Ex vivo* biodistribution and corresponding average ROI of Dox in tumor-bearing nude mice treated with PBS (control), Dox alone, Alkyn-nCOF@Dox, and Alkyn-nCOF-cRGD@Dox at the end of the treatment (20th day) of the liver. All data are presented as the mean ±SEM (n=5). ** p <0.05; ** p <0.01; *** p <0.001.*

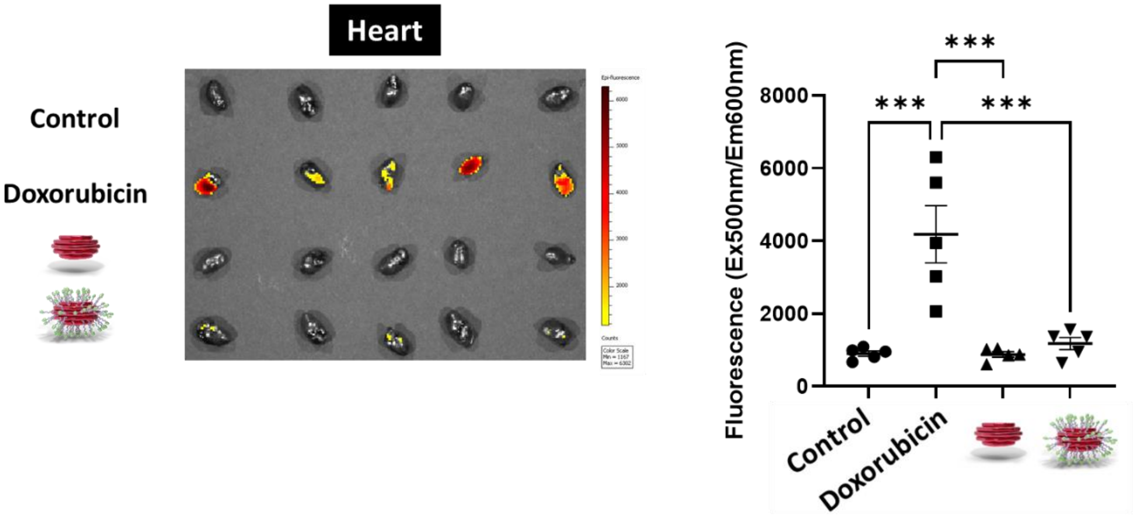

Figure S62. *Ex vivo* biodistribution and corresponding average ROI of Dox in tumor-bearing nude mice treated with PBS (control), Dox alone, Alkyn-nCOF@Dox, and Alkyn-nCOF-cRGD@Dox at the

end of the treatment (20th day) of heart. All data are presented as the mean ±SEM (n=5). ** p <0.05; ** p <0.01; *** p <0.001.*

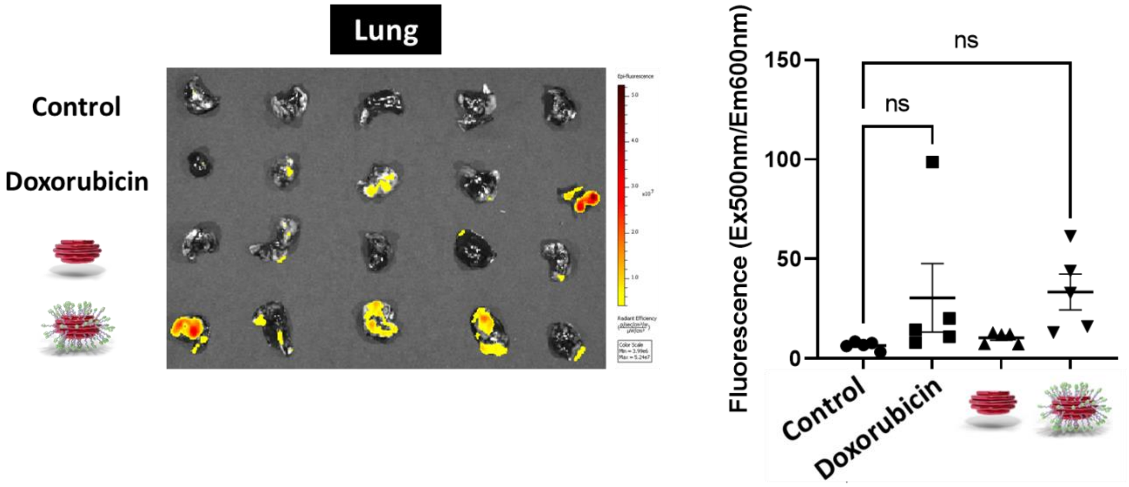

Figure S63. *Ex vivo* biodistribution and corresponding average ROI of Dox in tumor-bearing nude mice treated with PBS (control), Dox alone, Alkyn-nCOF@Dox, and Alkyn-nCOF-cRGD@Dox at the end of the treatment (20th day) of the lung. All data are presented as the mean ±SEM (n=5). ** p <0.05; ** p <0.01; *** p <0.001.*

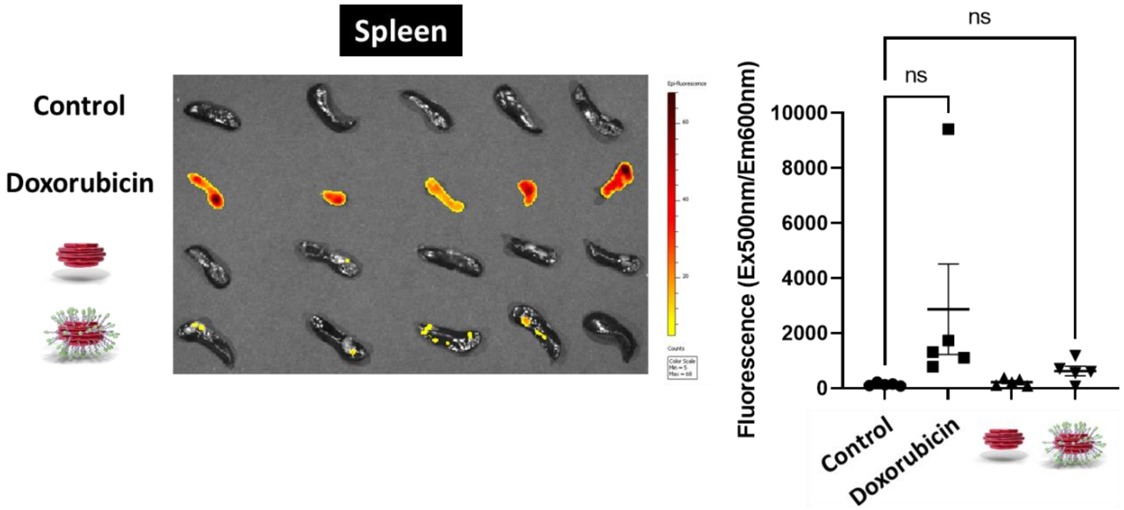

Figure S64. *Ex vivo* biodistribution and corresponding average ROI of Dox in tumor-bearing nude mice treated with PBS (control), Dox alone, Alkyn-nCOF@Dox, and Alkyn-nCOF-cRGD@Dox at the

end of the treatment (20th day) of the spleen. All data are presented as the mean ±SEM (n=5). *
*p <0.05; ** p <0.01; *** p <0.001.*

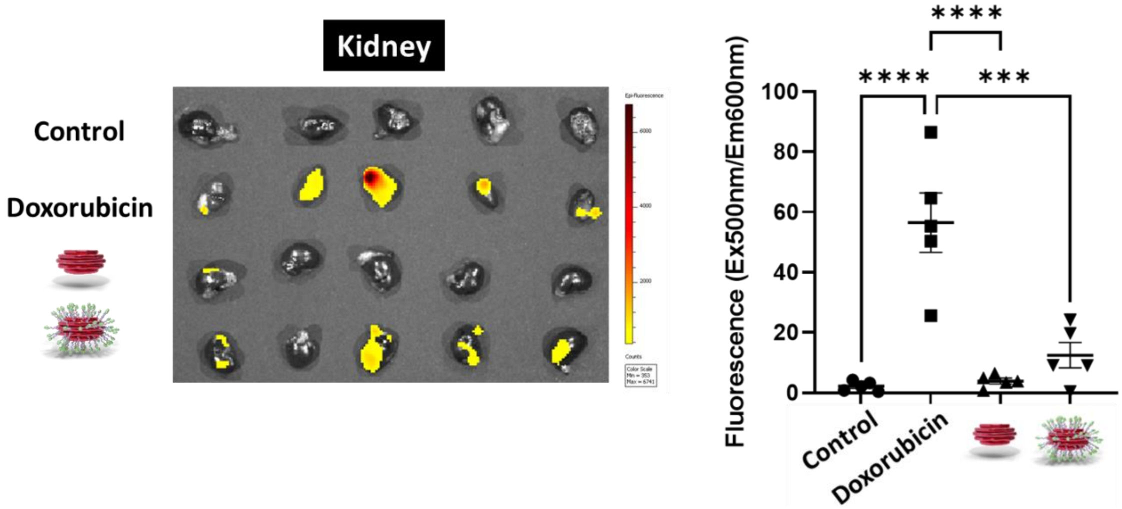

Figure S65. *Ex vivo* biodistribution and corresponding average ROI of Dox in tumor-bearing nude mice treated with PBS (control), Dox alone, Alkyn-nCOF@Dox, and Alkyn-nCOF-cRGD@Dox at the end of the treatment (20th day) of the kidney. All data are presented as the mean ±SEM (n=5). * *p <0.05; ** p <0.01; *** p <0.001.*

5.6. Statistical Analysis.

All statistical analysis was performed with GraphPad PRISM 8. All data are expressed as mean ±SD. Data were analyzed using one-way ANOVA with post hoc Tukey tests SPSS (IBM, SPSS Statistics, version 23, USA). * *p <0.05; ** p <0.01; *** p <0.001.*

References

1. Sheikh, A.; Alhakamy, N. A.; Md, S.; Kesharwani, P., Recent Progress of RGD Modified Liposomes as Multistage Rocket Against Cancer. *Front Pharmacol* **2021,** *12*, 803304.

2. Yin, L.; Li, X.; Wang, R.; Zeng, Y.; Zeng, Z.; Xie, T., Recent Research Progress of RGD Peptide–Modified Nanodrug Delivery Systems in Tumor Therapy. *International Journal of Peptide Research and Therapeutics* **2023,** *29* (4), 53.

3. Yadav, B.; Chauhan, M.; Shekhar, S.; Kumar, A.; Mehata, A. K.; Nayak, A. K.; Dutt, R.; Garg, V.; Kailashiya, V.; Muthu, M. S.; Sonali; Singh, R. P., RGD-decorated PLGA nanoparticles improved effectiveness and safety of cisplatin for lung cancer therapy. *International journal of pharmaceutics* **2023,** *633*, 122587.

4. Sun, Y.; Kang, C.; Liu, F.; Zhou, Y.; Luo, L.; Qiao, H., RGD Peptide-Based Target Drug Delivery of Doxorubicin Nanomedicine. *Drug development research* **2017,** *78* (6), 283-291.

5. Qin, W.; Chandra, J.; Abourehab, M. A. S.; Gupta, N.; Chen, Z. S.; Kesharwani, P.; Cao, H. L., New opportunities for RGD-engineered metal nanoparticles in cancer. *Molecular cancer* **2023,** *22* (1), 87.

6. Xiao, Y.; Hong, H.; Matson, V. Z.; Javadi, A.; Xu, W.; Yang, Y.; Zhang, Y.; Engle, J. W.; Nickles, R. J.; Cai, W.; Steeber, D. A.; Gong, S., Gold Nanorods Conjugated with Doxorubicin and cRGD for Combined Anticancer Drug Delivery and PET Imaging. *Theranostics* **2012,** *2* (8), 757-768.

7. Yanes, R. E.; Tamanoi, F., Development of mesoporous silica nanomaterials as a vehicle for anticancer drug delivery. *Ther Deliv* **2012,** *3* (3), 389-404.

8. Hazeri, Y.; Samie, A.; Ramezani, M.; Alibolandi, M.; Yaghoobi, E.; Dehghani, S.; Zolfaghari, R.; Khatami, F.; Zavvar, T.; Nameghi, M. A.; Abnous, K.; Taghdisi, S. M., Dual-targeted delivery of doxorubicin by mesoporous silica nanoparticle coated with AS1411 aptamer and RGDK-R peptide to breast cancer in vitro and in vivo. *Journal of Drug Delivery Science and Technology* **2022,** *71*, 103285.

9. Timmer, B. J. J.; Flos, M. A.; Jørgensen, L. M.; Proverbio, D.; Altun, S.; Ramström, O.; Aastrup, T.; Vincent, S. P., Spatially well-defined carbohydrate nanoplatforms: synthesis, characterization and lectin interaction study. *Chemical Communications* **2016,** *52* (83), 12326-12329.

10. Bolley, J.; Guenin, E.; Lievre, N.; Lecouvey, M.; Soussan, M.; Lalatonne, Y.; Motte, L., Carbodiimide versus Click Chemistry for Nanoparticle Surface Functionalization: A Comparative Study for the Elaboration of Multimodal Superparamagnetic Nanoparticles Targeting αvβ3 Integrins. *Langmuir* **2013,** *29* (47), 14639-14647.

11. Liu, S.; Hu, C.; Liu, Y.; Zhao, X.; Pang, M.; Lin, J., One-Pot Synthesis of DOX@Covalent Organic Framework with Enhanced Chemotherapeutic Efficacy. *Chemistry – A European Journal* **2019,** *25* (17), 4315-4319.

12. Zhang, G.; Li, X.; Liao, Q.; Liu, Y.; Xi, K.; Huang, W.; Jia, X., Water-dispersible PEG-curcumin/amine-functionalized covalent organic framework nanocomposites as smart carriers for in vivo drug delivery. *Nature Communications* **2018,** *9* (1), 2785.

13. Kandambeth, S.; Venkatesh, V.; Shinde, D. B.; Kumari, S.; Halder, A.; Verma, S.; Banerjee, R., Self-templated chemically stable hollow spherical covalent organic framework. *Nat. Commun.* **2015,** *6*, 6786.

14. Wang, B.; Liu, X.; Gong, P.; Ge, X.; Liu, Z.; You, J., Fluorescent COFs with a highly conjugated structure for visual drug loading and responsive release. *Chemical Communications* **2020,** *56* (4), 519-522.

15. Liu, S.; Yang, J.; Guo, R.; Deng, L.; Dong, A.; Zhang, J., Facile Fabrication of Redox-Responsive Covalent Organic Framework Nanocarriers for Efficiently Loading and Delivering Doxorubicin. *Macromol. Rapid Commun. 41* (41), 1900570.

16. Benyettou, F.; Das, G.; Nair, A. R.; Prakasam, T.; Shinde, D. B.; Sharma, S. K.; Whelan, J.; Lalatonne, Y.; Traboulsi, H.; Pasricha, R.; Abdullah, O.; Jagannathan, R.; Lai, Z.; Motte, L.; Gándara, F.; Sadler, K. C.; Trabolsi, A., Covalent Organic Framework Embedded with Magnetic Nanoparticles for MRI and Chemo-Thermotherapy. *Journal of the American Chemical Society* **2020,** *142* (44), 18782-18794.

17. Wang, C.; Liu, H.; Liu, S.; Wang, Z.; Zhang, J., pH and Redox Dual-Sensitive Covalent Organic Framework Nanocarriers to Resolve the Dilemma Between Extracellular Drug Loading and Intracellular Drug Release. *Frontiers in chemistry* **2020,** *8*.

18. Liu, S.; Yang, J.; Guo, R.; Deng, L.; Dong, A.; Zhang, J., Facile Fabrication of Redox-Responsive Covalent Organic Framework Nanocarriers for Efficiently Loading and Delivering Doxorubicin. *Macromolecular Rapid Communications* **2020,** *41* (4), 1900570.

19. Wang, K.; Zhang, Z.; Lin, L.; Hao, K.; Chen, J.; Tian, H.; Chen, X., Cyanine-Assisted Exfoliation of Covalent Organic Frameworks in Nanocomposites for Highly Efficient Chemo-Photothermal Tumor Therapy. *ACS Applied Materials & Interfaces* **2019,** *11* (43), 39503-39512.

20. Gao, P.; Zheng, T.; Cui, B.; Liu, X.; Pan, W.; Li, N.; Tang, B., Reversing tumor multidrug resistance with a catalytically active covalent organic framework. *Chemical Communications* **2021,** *57* (98), 13309-13312.

21. Huo, T.; Yang, Y.; Qian, M.; Jiang, H.; Du, Y.; Zhang, X.; Xie, Y.; Huang, R., Versatile hollow COF nanospheres via manipulating transferrin corona for precise glioma-targeted drug delivery. *Biomaterials* **2020,** *260*, 120305.

22. Zhou, F.; Fang, Y.; Deng, C.; Zhang, Q.; Wu, M.; Shen, H.-H.; Tang, Y.; Wang, Y., Templated Assembly of pH-Labile Covalent Organic Framework Hierarchical Particles for Intracellular Drug Delivery. *Nanomaterials* **2022,** *12* (17), 3055.

23. Gao, P.; Shen, X.; Liu, X.; Chen, Y.; Pan, W.; Li, N.; Tang, B., Nucleic Acid-Gated Covalent Organic Frameworks for Cancer-Specific Imaging and Drug Release. *Analytical Chemistry* **2021,** *93* (34), 11751-11757.

24. Anbazhagan, R.; Krishnamoorthi, R.; Kumaresan, S.; Tsai, H.-C., Thioether-terminated triazole-bridged covalent organic framework for dual-sensitive drug delivery application. *Materials Science and Engineering: C* **2021,** *120*, 111704.

25. Yao, S.; Zheng, M.; Wang, S.; Huang, T.; Wang, Z.; Zhao, Y.; Yuan, W.; Li, Z.; Wang, Z. L.; Li, L., Self-driven Electrical Stimulation Promotes Cancer Catalytic Therapy Based on Fully Conjugated Covalent Organic Framework Nanocages. *Advanced Functional Materials* **2022,** *32* (47), 2209142.

26. Zou, J.; Ren, X.; Tan, L.; Huang, Z.; Gou, L.; Meng, X., Preparation and properties of covalent organic framework nanoparticles with high drug loading. *Frontiers of Materials Science* **2021,** *15* (3), 465-470.

27. Wang, S.; Pang, Y.; Hu, S.; Lv, J.; Lin, Y.; Li, M., Copper sulfide engineered covalent organic frameworks for pH-responsive chemo/photothermal/chemodynamic synergistic therapy against cancer. *Chemical Engineering Journal* **2023,** *451*, 138864.
28. Fleisher, B.; Lezeau, J.; Werkman, C.; Jacobs, B.; Ait-Oudhia, S., In vitro to Clinical Translation of Combinatorial Effects of Doxorubicin and Abemaciclib in Rb-Positive Triple Negative Breast Cancer: A Systems-Based Pharmacokinetic/Pharmacodynamic Modeling Approach. *Breast cancer (Dove Medical Press)* **2021,** *13*, 87-105.
